# MdMYB8 is associated with flavonol biosynthesis via the activation of the *MdFLS* promoter in the fruits of *Malus* crabapple

**DOI:** 10.1038/s41438-020-0238-z

**Published:** 2020-02-01

**Authors:** Hua Li, Yu Li, Jiaxuan Yu, Ting Wu, Jie Zhang, Ji Tian, Yuncong Yao

**Affiliations:** 1 Beijing Advanced Innovation Center for Tree Breeding by Molecular Design, Beijing University of Agriculture, Beijing, China; 2 Department of Plant Science and Technology, Beijing University of Agriculture, Beijing, China; 3 College of Horticulture, China Agricultural University, Beijing, China

**Keywords:** Secondary metabolism, DNA sequencing

## Abstract

Flavonols are polyphenolic compounds that play important roles in plant stress resistance and development. They are also valuable components of the human diet. The *Malus* crabapple cultivar ‘Flame’ provides an excellent model for studying flavonol biosynthesis due to the high flavonol content of its fruit peel. To obtain a more detailed understanding of the flavonol regulatory network involved in fruit development, the transcriptomes of the fruit of the *Malus* cv. ‘Flame’ from five continuous developmental stages were analyzed using RNA sequencing. A flavonol-related gene module was identified through weighted gene coexpression network analysis (WGCNA), and Kyoto Encyclopedia of Genes and Genomes (KEGG) analysis indicated that phytohormones are involved in regulating flavonol biosynthesis during fruit development. A putative transcription factor, *MdMYB8*, was selected for further study through hub gene correlation network analysis and yeast one-hybrid assays. Stable overexpression or RNAi knockdown of *MdMYB8* in transgenic ‘Orin’ apple calli resulted in a higher or lower flavonol content, respectively, suggesting that *MdMYB8* is a regulator of flavonol biosynthesis. This transcriptome analysis provides valuable data for future studies of flavonol synthesis and regulation.

## Introduction

Flavonoids are secondary metabolites that are synthesized via the phenylpropanoid pathway in plants, which gives rise to three main kinds of compounds: flavonols, proanthocyanidins (PAs), and anthocyanins^1^. They play roles in various processes, such as protection against oxidation and ultraviolet-B radiation, auxin distribution and transport regulation, pollen recognition control, the modulation of leaf and flower color, and signaling to symbiotic organisms in plants^2,3^. In addition, dietary flavonols have been indicated to act as protective molecules in mammals^4^.

Flavonol biosynthesis is located on the downstream branch of the general flavonoid pathway supplying the common flavonoid dihydroflavonol precursors, which serve as biosynthetic substrates for dihydroflavonol 4-reductase (DFR) or flavonol synthase (FLS). There is competition between these two enzymes, leading to either flavonol or anthocyanin biosynthesis, respectively^5^. The *FLS* gene was first cloned from Petunia (*Petunia hybrida*)^6^, and since then, *FLS* genes have been cloned and functionally analyzed in various plants, such as Arabidopsis (*Arabidopsis thaliana*)^7^, grapevine (*Vitis vinifera*)^8^, and *Vaccinium corymbosum*^9^.

The R2R3-MYB TF family, which is the largest MYB subfamily, has been demonstrated to act as the main flavonoid biosynthesis regulator in many plant species^10–17^. In *A. thaliana*, AtMYB11, AtMYB12, and AtMYB111 have been shown to interact with the promoters of chalcone synthase (CHS), flavanone 3-hydroxylase (F3H) and FLS^14^. In citrus (*Citrus sinensis*), CsMYBF1 was proven to activate the expression of several flavonoid biosynthetic pathway genes and to control flavonol and hydroxycinnamic acid biosynthesis^11^, while in pear (*Pyrus bretschneideri*), PbMYB9 not only acts as an activator of the PA biosynthetic pathway by activating the *PbANR* (anthocyanidin reductase) promoter but also induces the accumulation of anthocyanins and flavonols by binding to the promoter of *PbUFGT1* (UDP-glucose flavonoid 3-*O*-glucosyltransferase)^15^. Finally, in apples (*Malus sieversii* f. *niedzwetzkyana*), MdMYB22 was found to promote flavonol accumulation by directly binding the *MdFLS* promoter^17^. These findings highlight the diversity of flavonol regulation in different fruit types and species.

*Malus* crabapples belong to family Rosaceae, genus *Malus* Mill., and are ornamental plants with important economic value^18^. The high levels of flavonol compounds in the fruits of the *Malus* crabapple variety ‘Flame’ provide a valuable model for studying the molecular mechanisms of flavonol biosynthesis^18^. To obtain insights into the transcriptional regulation of the flavonol biosynthetic pathways in *Malus* crabapple, we performed RNA-seq analysis of five different ‘Flame’ fruit developmental stages. We used this information to identify hub genes through WGCNA and functionally verified them using yeast one-hybrid (Y1H) assays and transgenic apple (*Malus domestica* cv. ‘Orin’) calli. The goal was to obtain a more detailed understanding of the flavonol regulatory network during crabapple fruit development and to provide a theoretical basis for the future breeding of apples with high nutritional value in the form of a high flavonol content.

## Results

### Transcriptome analysis of fruit developmental stages

**Fig. 1:**
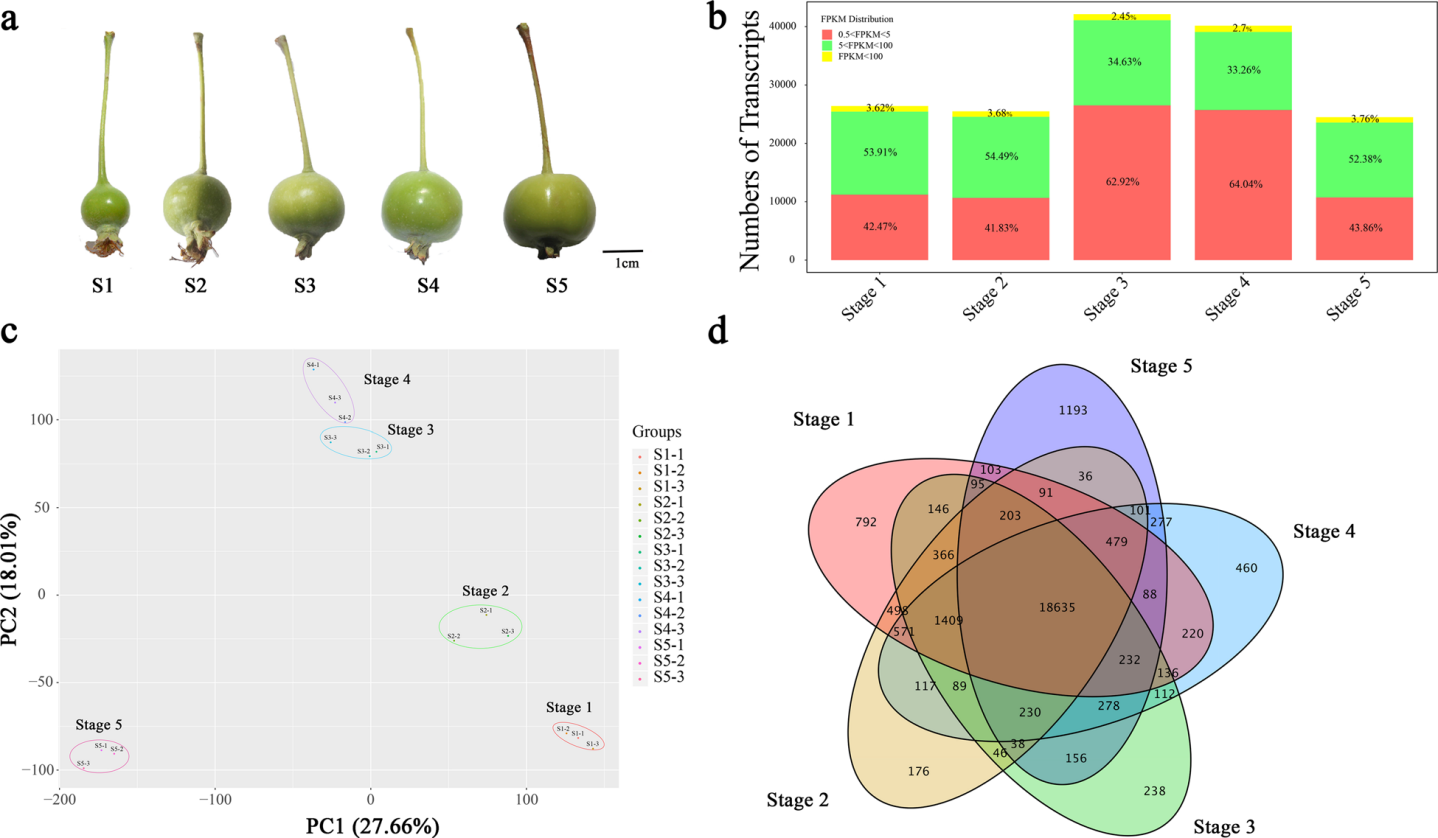
RNA-seq data expression profiles during fruit development in *Malus* ‘Flame’ fruits. **a** Fruit phenotypes of ‘Flame’ fruit at five typical development stages. **b** Numbers of transcripts in five fruit developmental stages. **c** Principal component analysis of the RNA-seq data. **d** Venn diagram of the RNA-seq data from five fruit developmental stages

The evergreen fruit cultivar ‘Flame’ provides an excellent model for studying flavonol biosynthesis due to the high flavonol content of its fruit peel^18^. To obtain a more detailed understanding of the flavonol regulatory network during fruit development, fruit peels from five different developmental stages of the *Malus* cv. ‘Flame’ (35, 60, 95, 120, and 150 days after full bloom) was used as a source of material for an RNA-seq study (Fig. 1a).

In total, three biological replicates from each of the five stages were analyzed, and the total lengths of the clean reads ranged from 11,130,344 to 13,182,947. Between 82.96% and 88.64% of the sequenced reads could be aligned to the apple reference genome. The Q30 percentages of all 15 libraries were >90% (Table 1). Subsequently, reads with a fragments per kilobase of transcript per million mapped reads (FPKM) value <0.5 were removed, and 26,399, 25,492, 42,133, 40,160, and 24,490 transcripts were found to be expressed in Stage 1-Stage 5, respectively. Approximately 51% of the expressed genes were in the 0.5-5 FPKM range, and 46% were in the 5-100 FPKM range (Fig. 1b). A principal component analysis and Pearson correlation analysis showed that there were highly correlated transcriptome characteristics (*r*^2^ = 0.858–0.986) between the biological replicates of each developmental stage (Fig. 1c; Supplementary Fig. S1). The proportion of exon sequences ranged from 43 to 57%, and the proportion of intron sequences ranged from 2 to 5% (Supplementary Fig. S2). A total of 18,635 transcripts were shared between Stage 1 and Stage 5 (Fig. 1d).

**Table 1:** RNA sequencing data and corresponding quality control

Sample name	Clean reads	GC Content (%)	%≧Q30 (%)	Total reads	Mapped reads
S1-1	11,471,744	47.77	94.06	22,943,488	19,629,749 (85.56%)
S1-2	11,217,270	47.79	94.28	22,434,540	19,071,727 (85.01%)
S1-3	11,683,772	47.59	94.25	23,367,544	19,728,654 (84.43%)
S2-1	13,583,562	47.94	95.52	27,167,124	23,669,464 (87.13%)
S2-2	12,276,781	47.94	95.34	24,553,562	21,249,955 (86.55%)
S2-3	12,767,096	47.79	95.26	25,534,192	22,123,993 (86.64%)
S3-1	11,976,085	47.30	92.67	23,952,170	20,263,666 (84.60%)
S3-2	11,037,203	47.44	92.68	22,047,406	18,499,514 (83.81%)
S3-3	11,524,221	47.10	92.67	23,048,442	19,510,415 (84.65%)
S4-1	12,151,986	47.79	91.73	24,303,972	20,334,006 (83.67%)
S4-2	11,130,344	47.95	90.97	22,260,688	18,466,928 (82.96%)
S4-3	10,430,201	47.82	92.32	20,860,402	17,728,619 (84.99%)
S5-1	10,994,666	48.15	95.34	21,989,332	19,275,480 (87.66%)
S5-2	13,182,947	47.78	95.36	26,365,894	23,240,421 (88.15%)
S5-3	11,064,319	47.55	95.33	22,128,638	19,615,696 (88.64%)

### Comparisons of differentially expressed genes between different developmental stages

To identify differentially expressed genes (DEGs) during fruit development, we conducted comparisons between five developmental stages. The DEGs were filtered according to an expression level |log_2_(fold-change)| > 1 and FDR < 0.05 in each pairwise comparison. The results showed DEG enrichment in Stage 2 vs. Stage 3 and Stage 4 vs. Stage 5, and indicated that the number of downregulated DEGs was significantly higher than the number of upregulated DEGs throughout fruit development (Supplementary Fig. S3).

**Fig. 2:**
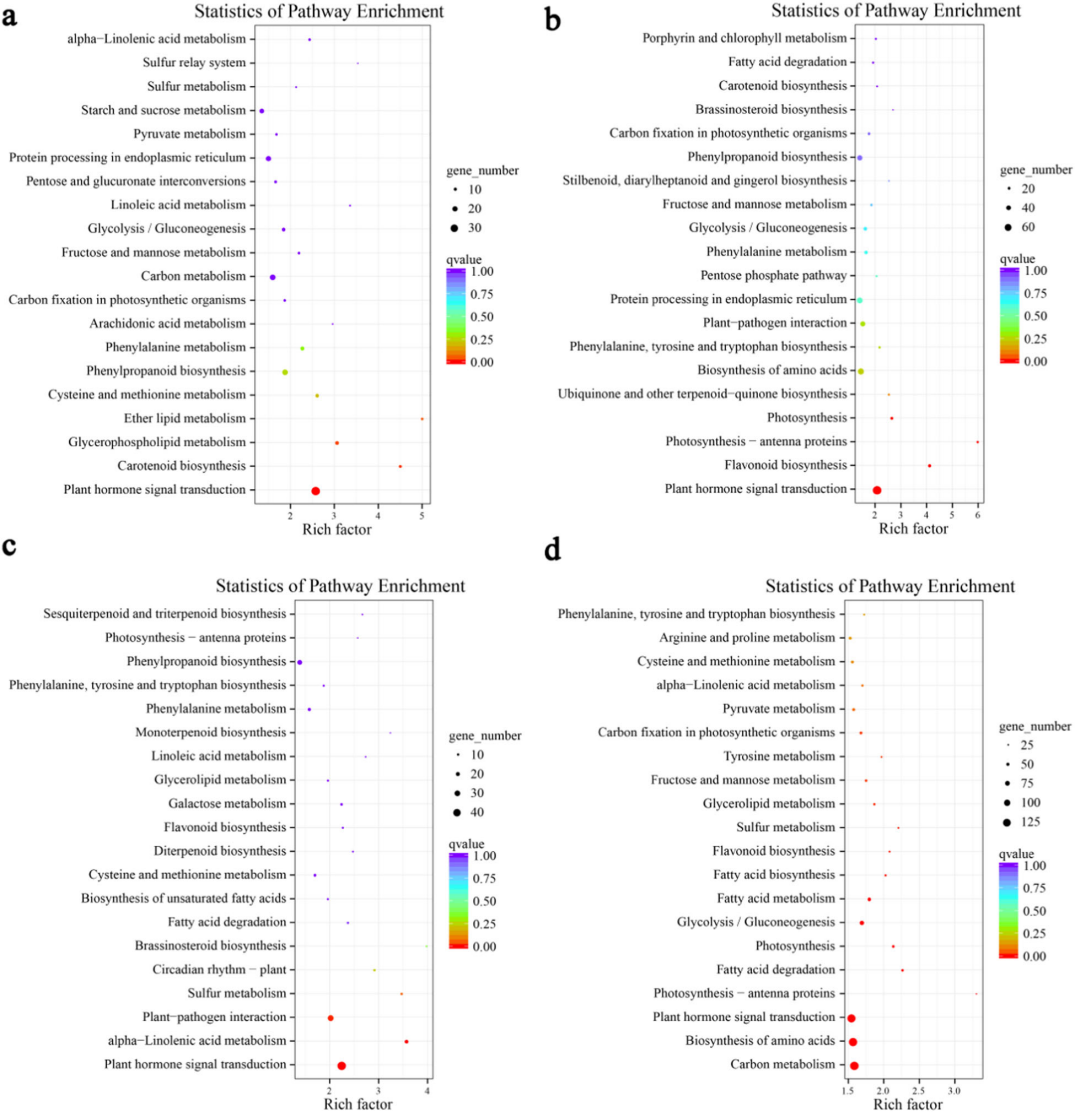
KEGG analysis of DEGs identified from pairwise comparisons between developmental stages during fruit development. **a–d** Stage 1 vs. Stage 2 (**a**), Stage 2 vs. Stage 3 (**b**), Stage 3 vs. Stage 4 (**c**), and Stage 4 vs. Stage 5 (**d**). The *Q* value is the multiple hypothesis test-corrected *P* value. The *q* value ranges from [0-1]. The closer that number is to 0, the more significant the enrichment is. The rich factor refers to the ratio of the number of genes among the DEGs located in a number of pathways to the total number of genes in the pathway entries in all of the annotated genes. The greater the rich factor, the greater the degree of enrichment is^49^

A Kyoto Encyclopedia of Genes and Genomes (KEGG) analysis of the pairwise comparison results provided additional suggestions about the biological functions of the identified DEGs, with enrichment in the following pathways: ‘plant hormone signal transduction’, ‘phenylpropanoid biosynthesis’, ‘phenylalanine metabolism’, ‘glycolysis/gluconeogenesis’, ‘phenylalanine, tyrosine and tryptophan biosynthesis’, ‘fructose and mannose metabolism’, ‘flavonoid biosynthesis’ and ‘starch and sucrose metabolism’ (Fig. 2). These biological processes are all closely related to flavonoid biosynthesis^19^. We noted that among the DEGs associated with phytohormones, those involved in auxin and abscisic acid (ABA) signal transduction or encoding proteins responsive to these hormones were enriched throughout fruit development compared to stage 1, while those involved in gibberellin (GA), ethylene (Eth), and jasmonic acid (JA) signal transduction or encoding proteins responsive to these hormones were enriched in middle and late developmental stages compared to stage 1 (Supplementary Table 1).

### Identification of WGCNA modules related to flavonol biosynthesis

**Fig. 3:**
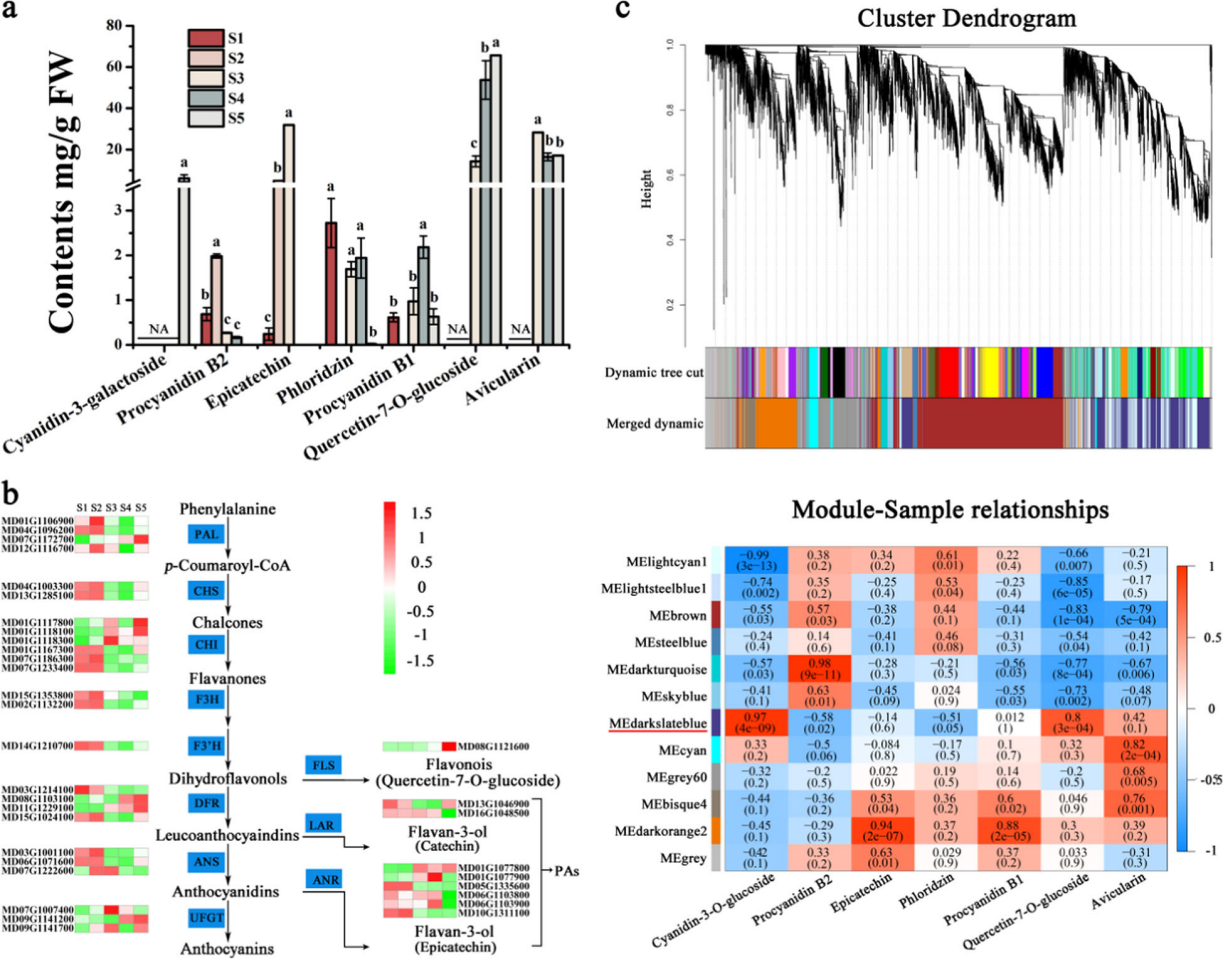
DEGs related to different flavonoid compounds were identified by WGCNA. **a** HPLC analysis of the flavonoid compounds in five ‘Flame’ fruit developmental stages. **b** Expression analysis of flavonoid pathway genes by RNA-seq in five fruit developmental stages. **c** Hierarchical clustering tree and module-flavonoid associations. The module with the highest correlation between flavonol biosynthesis and gene expression is indicated by red underlining of the module name

To characterize the flavonoid contents of the fruit peel during development, we used high-performance liquid chromatography (HPLC) to analyze extracts from five developmental stages (35, 60, 95, 120, and 150 days after full bloom). Quercetin-7-*O*-glucoside was identified as the main flavonoid compound, and its abundance gradually increased during development, with a rapid increase at stage 3 and a peak at stage 5 (Fig. 3a). In contrast, the contents of procyanidin B2, phloridzin, and avicularin decreased, and the levels of epicatechin and procyanidin B1 increased with development, while anthocyanin (cyanidin-3-galactoside) was only detected in stage 5 (Fig. 3a).

We observed that the expression of *FLS* (MD08G1121600) increased with fruit development, while the expression of the proanthocyanidin (PA) biosynthesis genes *LAR* (leucoanthocyanidin reductase) (MD16G1048500) and *ANR* (anthocyanidin reductase) (MD05G1335600, MD06G1103800, MD06G1103900, and MD10G1311100) decreased, and that of *LAR* (MD13G1046900) and *ANR* (MD01G1077800, MD01G1077900) increased. The expression levels of *PAL* (MD01G1106900, MD04G1096200, MD07G1172700, and MD12G1116700), *CHS* (MD04G1003300, MD13G1285100), *CHI* (MD01G1117800, MD01G1118100, MD01G1118300, MD01G1167300, MD07G1186300, and MD07G1233400), *DFR* (MD03G1214100, MD08G1103100, MD11G1229100, and MD15G1024100), *ANS* (MD03G1001100, MD06G1071600, and MD07G1222600) and *UFGT* (MD07G1007400, MD09G1141200, and MD09G1141700), which are related to anthocyanin biosynthesis, did not change, whereas that of the *F3H* (MD02G1132200, MD15G1353800) and *F3’H* (MD14G1210700) genes decreased (Fig. 3b). In all cases, there was a correlation between compound abundance and the expression levels of the associated biosynthetic genes (Fig. 3a). The qRT-PCR results showed that the expression levels of flavonoid biosynthesis genes were consistent with the RNA-seq data (Fig. S4).

Weighted gene coexpression network analysis (WGCNA) is a method for identifying candidate hub genes associated with certain functions or traits^20^. WGCNA was used to identify putative transcription factors (TFs) that regulate flavonoid metabolism during fruit development. A total of 8681 DEGs were included in the analysis, and 12 distinct modules were revealed, which are labeled in different colors (Fig. 3c, Supplementary Table S2). The MEdarkslateblue module (1662 genes) presented the highest correlation with flavonol (quercetin-7-*O*-glucoside) accumulation, with a correlation coefficient of 0.8.

### KEGG analysis of genes in the MEdarkslateblue module

**Fig. 4:**
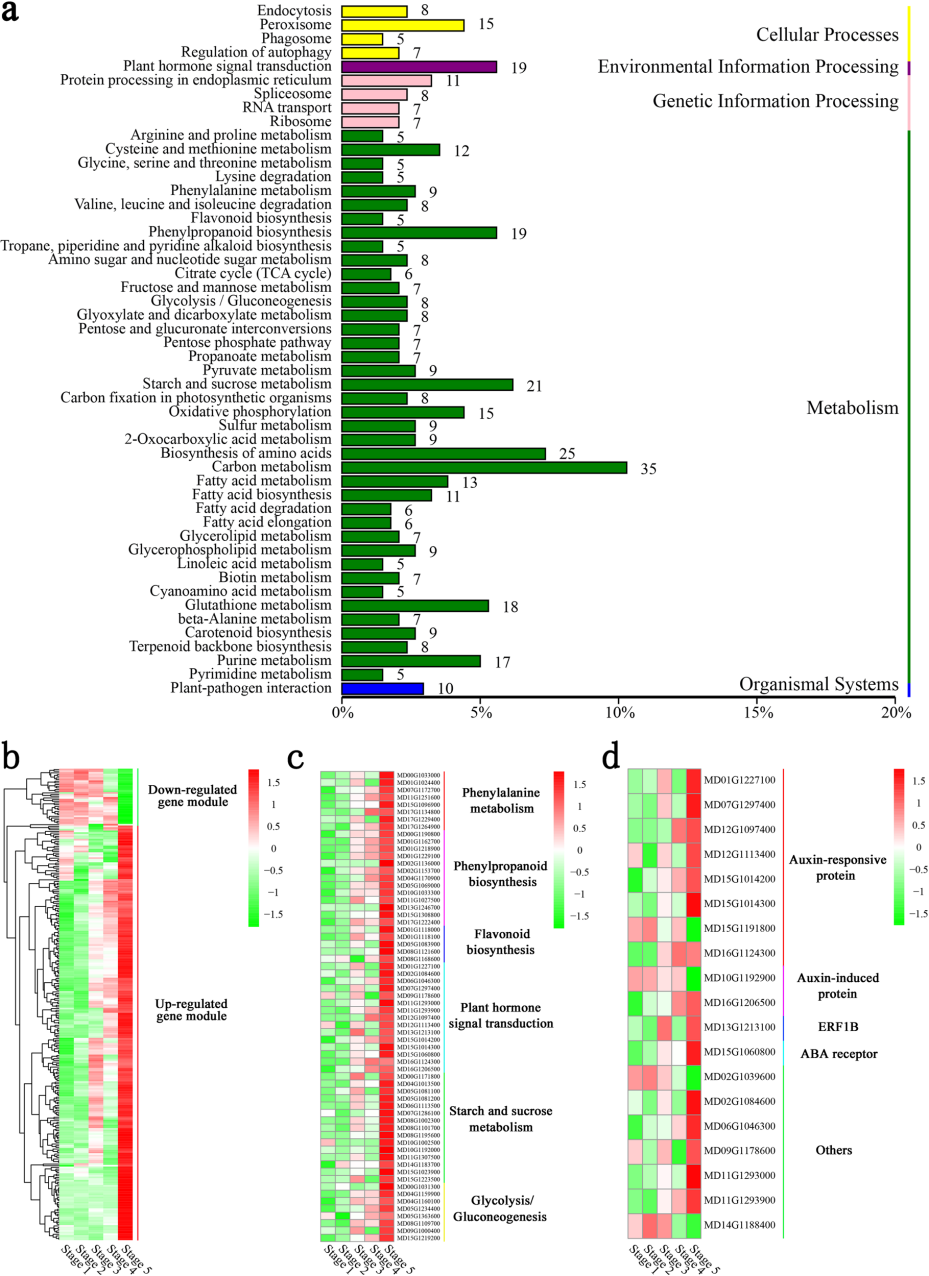
Functional categories and comparisons of DEGs in the MEdarkslateblue module. **a** KEGG pathway enrichment analysis of DEGs in the MEdarkslateblue module. **b** Clustering heat maps of significantly enriched MEdarkslateblue module-specific genes. **c** Heat map comparison of DEGs associated with flavonoid biosynthesis in the upregulated gene module. **d** DEG categories related to plant hormone signal transduction in the MEdarkslateblue module

**Fig. 5:**
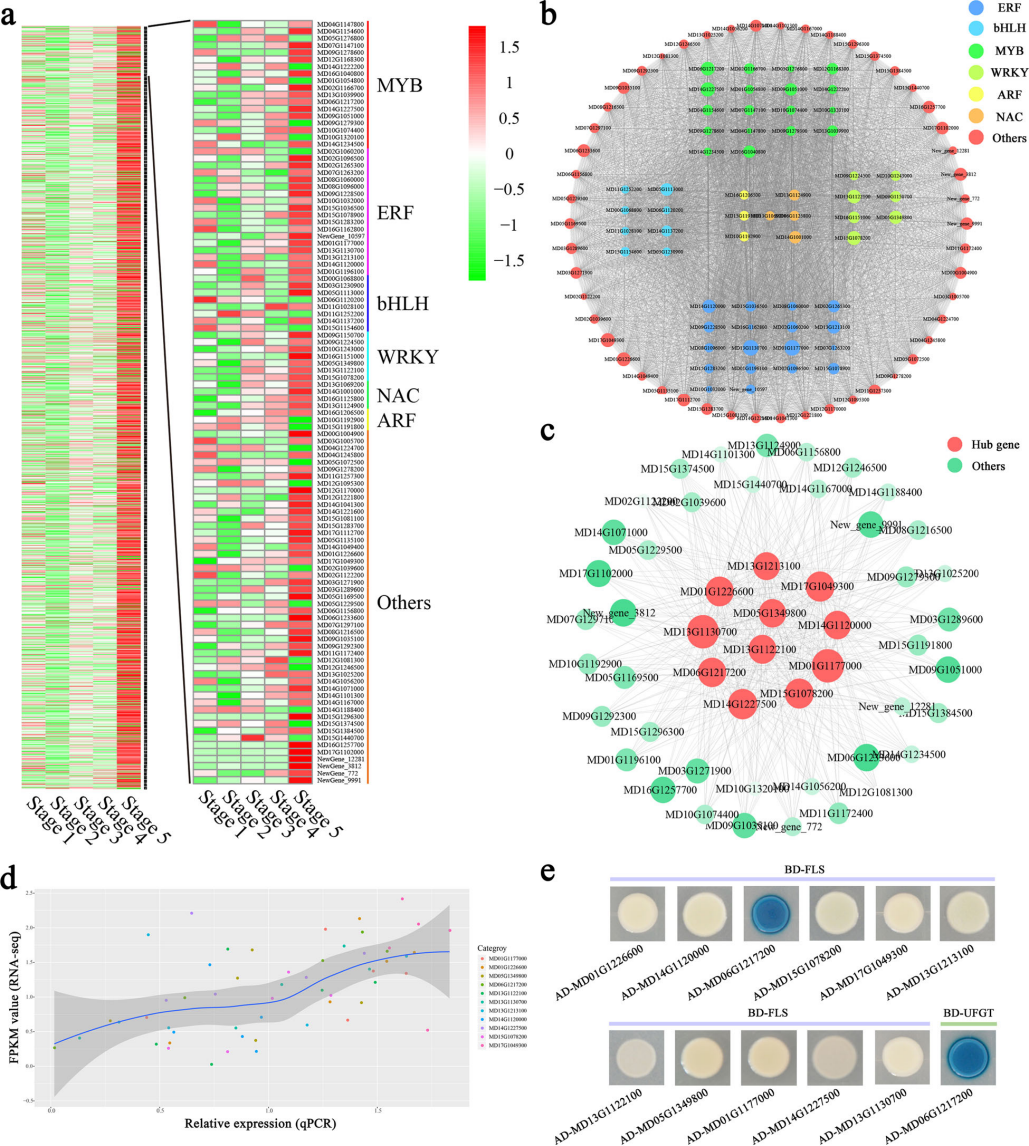
Identification and selection of hub genes associated with flavonoid biosynthesis in the MEdarkslateblue module. **a** Heat map comparison and transcription factor (TF) categories of differentially expressed genes (DEGs) in the MEdarkslateblue module. **b** Network analysis of the 108 TFs in the MEdarkslateblue module. **c** Network of the top 11 hub genes and related genes from the MEdarkslateblue module. **d** Scatter plot of gene expression data obtained through RNA-seq and qRT-PCR. **e** Yeast one-hybrid analysis of interactions between selected TFs and the flavonol biosynthesis gene *FLS* or the anthocyanin biosynthesis gene *UFGT*

The KEGG database was used to determine the main biological pathways associated with the MEdarkslateblue module (Fig. 5a). Heat maps of the clustering analysis results suggested that the upregulated expressed genes were obviously enriched in the MEdarkslateblue module. Genes related to the ‘starch and sucrose metabolism’, ‘glycolysis/gluconeogenesis’, ‘plant hormone signal transduction’, ‘phenylalanine metabolism’, ‘phenylpropanoid biosynthesis’, and ‘flavonoid biosynthesis’ pathways were significantly enriched in the upregulated gene module (Fig. 4b, c).

Further analysis of the DEGs related to ‘hormone signal transduction’ revealed that auxin-responsive and auxin-induced proteins were significantly enriched and showed a continuous increase in expression during fruit development (Fig. 4d). The qRT-PCR results showed that the expression patterns of auxin-responsive proteins (MD12G1097400, MD15G1014200, and MD16G1124300) and auxin-induced proteins (MD16G1206500) were positively correlated with the accumulation of querectin-3-O-glucoside during fruit development (Fig. S5a). The reliability of the RNA-seq data for these hormone signal transduction-related genes was confirmed through qRT-PCR (Fig. S5b). In comparison, ethylene and ABA signal transduction or responsive proteins were upregulated in a more stage-specific manner (Fig. 4d).

### Identification of hub genes associated with flavonoid biosynthesis

Highly connected nodes in expression networks were defined as hub genes, which are often associated with biological processes and interactions^21^. Many DEGs identified in the MEdarkslateblue module were annotated as TFs, including several MYB, ERF, bHLH, and WRKY TFs. Their relative expression levels are shown in Fig. 5a. Eleven of these TFs were selected as being of particular interest based on the hub gene correlation network and their expression patterns: ethylene-responsive transcription factor 2 (MD01G1177000), homeobox-leucine zipper protein HOX6 (MD01G1226600), WRKY transcription factor 6 (MD05G1349800), MYB transcription factor 8 (MD06G1217200), WRKY transcription factor 75 (MD13G1122100), ethylene-responsive transcription factor ERF115 (MD13G1130700), ethylene-responsive transcription factor 1B (MD13G1213100), ethylene-responsive transcription factor CRF2 (MD14G1120000), 40S ribosomal protein S7 (MD14G1227500), WRKY transcription factor 7 (MD15G1078200), and the bHLH135 transcription factor (MD17G1049300) (Fig. 5b, c). The reliability of the RNA-seq data confirmed the expression of these 11 genes. The correlation between qRT-PCR and RNA-seq data supported the reliability of candidate hub gene selection (Fig. 5d).

We used Y1H analysis to detect the binding ability of the 11 proteins encoded by the 11 TF genes to the *FLS* promoter. An interaction between MD06G1217200 (MYB8) and the promoter of *MdFLS* was observed, while no interactions were observed for any other candidate. In addition, we observed an interaction between MYB8 and a construct derived from the *UFGT* promoter, BD-UFGT.

### Functional analysis of the role of *MdMYB8* in the flavonoid biosynthetic pathway

The 879-bp *MdMYB8* cDNA sequence is predicted to encode 292 amino acids and to possess 93%, 92% and 87% sequence identity to the MYB TFs MdWER-like1 (Accession: XP_018500861), MdWER-like2 (Accession: XP_009347244), and MdMYB62-like (Accession: XP_008367580), respectively (Fig. S6a). A multiple sequence alignment and phylogenetic tree of MdMYB8 (MD06G1217200) and other MYB TFs that have been reported to be associated with anthocyanin, PA, or flavonol biosynthesis were generated (Fig. S6b), revealing clades associated with these compound classes. MdMYB8 (MD06G1217200) shared higher sequence similarity with anthocyanin regulators than other proteins (Fig. S6b). We also cloned the full-length MYB8 cDNA from crabapple cDNA libraries to identify possible sequence differences, and sequence alignment showed that the MYB8 sequences were 100% similar in different crabapple cultivars, including ‘Flame’, ‘Royalty’ and ‘India magic’ (Fig. S6c).

To further assess the role of *MdMYB8* (MD06G1217200) in flavonoid biosynthesis, it was overexpressed or knocked down by RNA interference using the vector pBI101-*MdMYB8* (for overexpression) or pBI101-RNAi-*MdMYB8* (for RNAi), respectively, under the control of the 35S promoter in apple (*Malus domestica* cv. ‘Orin’) calli. The calli transformed with pBI101-*MdMYB8* or pBI101-RNAi-*MdMYB8* showed no significant color difference compared to the control calli. The accumulation patterns of flavonols in the transgenic calli were visualized in situ by diphenylboric acid-2-aminoethylester (DPBA) staining. The *MdMYB8*-overexpressing calli showed intense orange fluorescence after 1 week of light treatment, indicating substantial accumulation of quercetin, and the fluorescence of *MdMYB8*-RNAi calli was weaker than that of the control.

**Fig. 6:**
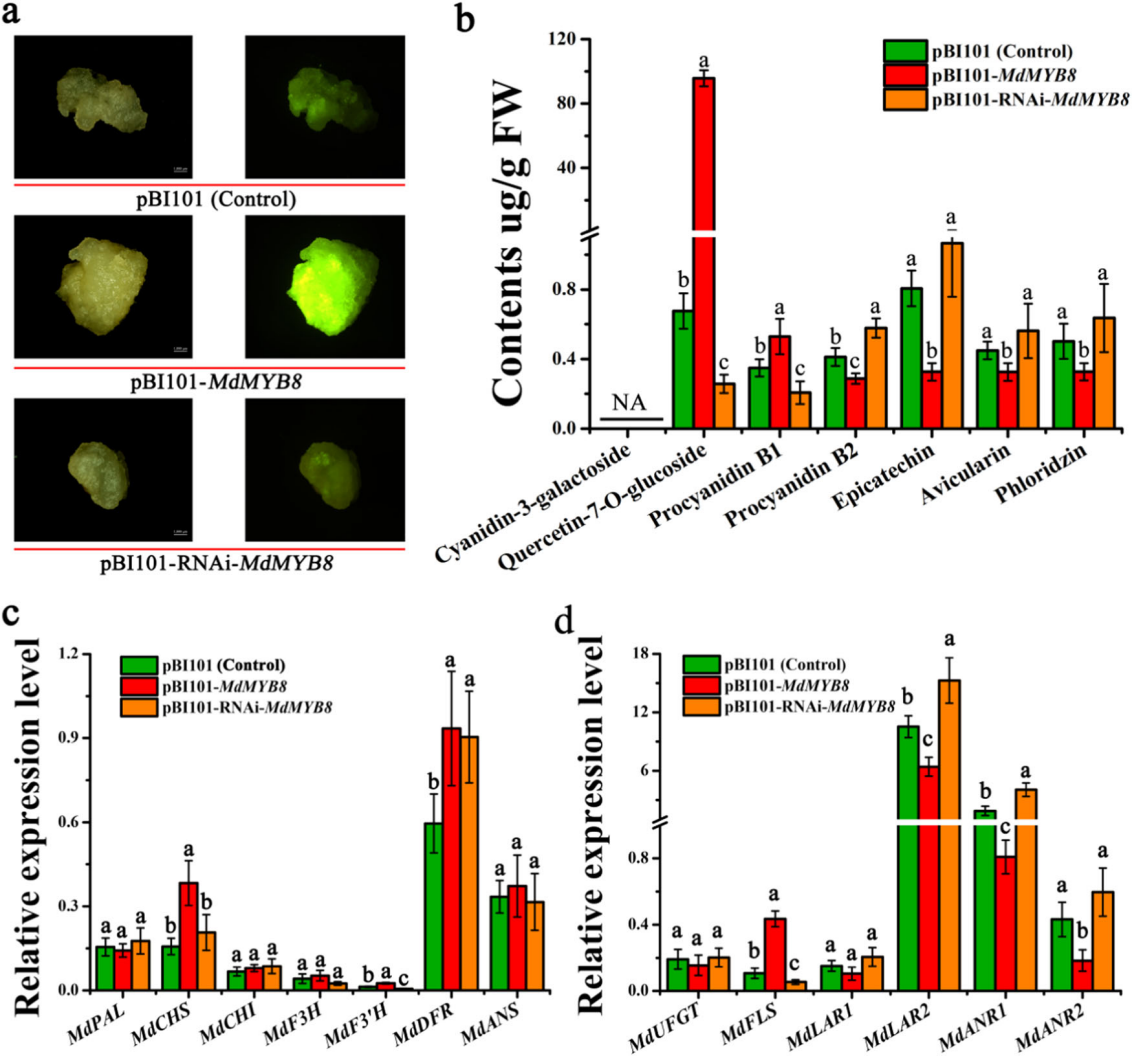
Overexpression or RNAi knockdown of *MdMYB8* (MD06G1217200) in apple calli (*Malus domestica* cv. ‘Orin’). **a** The control (pBI101), *MdMYB8*-overexpressing (pBI101-*MdMYB8*), and *MdMYB8*-knockdown (pBI101-RNAi-*MdMYB8*) apple calli were observed in daylight and under UV light. Calli were stained with the flavonol-specific dye DPBA. **b** Contents of flavonoid compounds in *MdMYB8* (MD06G1217200)-overexpressing ‘Orin’ apple calli and those subjected to RNAi. **c**, **d** qRT-PCR analysis of flavonoid biosynthetic genes in the *MdMYB8* (MD06G1217200)-overexpressing ‘Orin’ apple calli and those subjected to RNAi. qRT-PCR and HPLC analyses were performed on three biological replicates. Error bars indicate the standard error of the mean ± SE of three replicate measurements. Different letters above the bars indicate significantly different values (*P* *<* 0.05), calculated using one-way analysis of variance (ANOVA) followed by Tukey’s multiple range test

HPLC analysis also indicated significantly higher accumulation of flavonol (quercetin-7-O-glucoside) and procyanidin B1 in *MdMYB8*-overexpressing calli, while the contents of procyanidin B2, epicatechin, avicularin, and phloridzin were significantly decreased (Fig. 6b). Opposite results were observed in *MdMYB8*-RNAi calli. The qRT-PCR results suggested that the expression levels of the flavonol-related *MdF3*′*H* and *MdFLS* genes and the PA-related *MdLAR1*, *MdLAR2*, *MdANR1*, and *MdANR2* genes were significantly higher or lower in the *MdMYB8*-overexpressing or *MdMYB8*-RNAi calli, respectively, compared to the control (Fig. 6c). The expression of the anthocyanin-related *MdPAL*, *MdCHI*, *MdF3H*, *MdANS*, and *MdUFGT* genes showed no significant change compared with the control calli (Fig. 6c).

## Discussion

### Characterization of *Malus* ‘Flame’ fruit development

Flavonoid content plays a role in determining the quality of many fruits and is often a major factor affecting coloration, astringency, and bitterness, and enhancing ruminant nutrition^19,22^. The overarching goal of this research was to select genes associated with the regulation of flavonol biosynthesis in ‘Flame’ crabapple. A KEGG analysis of DEGs revealed that flavonoid biosynthesis-related biological processes were significantly enriched during fruit development (Fig. 2, Supplementary Table S1). Quercetin-7-*O*-glucoside was identified as the major flavonoid, and flavonol levels were observed to increase during fruit development (Fig. 3).

### The role of hormones in fruit development

Plant hormones are considered to show a close relationship with fruit development, including fruit set, growth, maturation, and ripening^23,24^. Previous studies have indicated that auxin, GA, and cytokinin are major regulators of fruit set, auxin and cytokinin are key factors in fruit growth, auxin and ethylene are the primary regulators of fruit maturation, and ethylene and ABA are central to fruit ripening^23,24^. Current evidence suggests that various hormones play important roles in flavonoid accumulation. In *A. thaliana*, GA, JA, and ABA modulate the expression of anthocyanin biosynthetic genes induced by sucrose^25^. In strawberry (*Fragaria* × *ananassa*), the auxin compound indole-3-acetic acid (IAA) is involved in fruit color development through the regulation of anthocyanin biosynthesis, while anthocyanin accumulation is promoted by ABA and methyl jasmonate (MJ) in strawberry fruits^26,27^. In grapevine, exogenous ethylene application to grape berries stimulates the transcription of *CHS*, *F3H*, *LDOX*, and *UFGT* and promotes anthocyanin accumulation ^28^ and in litchi (*Litchi chinensis Sonn*.), the exogenous application of ABA to fruit can increase *UFGT* expression and promote anthocyanin accumulation^29^. Finally, in apple (*Malus domestica*), IAA, ethylene and ABA increase anthocyanin accumulation, while GA has the opposite effect^30,31^.

In our studies, a large number of DEGs relevant to plant hormones, including those encoding proteins related to auxin, ethylene, ABA, JA, and GA signal transduction or responsiveness, were significantly enriched with fruit development, consistent with the importance of these pathways in ‘Flame’ fruit development (Fig. 2; Supplementary Table S1). DEGs associated with auxin were mainly enriched during early developmental stages, upregulated DEGs associated with ethylene and ABA were characterized by stage-specific expression, JA-related DEGs were always downregulated in middle and late developmental stages, and GA-related DEGs were upregulated during late developmental stages (Fig. 2, Supplementary Table S1). Largely consistent results were previously observed in strawberry fruit, in which IAA contents were reported to be highest at Stage 1 and to gradually decrease with development, while the ABA content was low from Stage 1 to Stage 3 and peaked at S6^32^. These results are consistent with auxin being important early in development, while ethylene and ABA contribute to fruit enlargement and ripening, and GA plays a role during later developmental stages in ‘Flame’ fruit.

The analysis of the DEGs related to ‘hormone signal transduction’ in the MEdarkslateblue module further suggested that auxin, ethylene, and ABA may be important for flavonol and anthocyanin biosynthesis regulation (Fig. 4d). In addition, the expression of auxin-responsive and auxin-induced proteins increased overall during fruit development, while proteins related to ethylene and ABA signal transduction or responsiveness were characterized by stage-specific upregulation (Fig. 4d). Notably, proteins related to JA signal transduction or responsiveness were not significantly enriched in the MEdarkslateblue module, while JA-related DEGs were significantly enriched and consistently downregulated during middle and late developmental stages (Fig. 2, Supplementary Table 1). Previous studies have suggested that TIR1 and EIN2/ETR1 regulate flavonol biosynthesis by auxin and ethylene signaling networks in *A. thaliana*^33^. In addition, JA participates in the control of flavonol biosynthesis by stimulating *PAL5*, *CHS*, and *FLS* transcription in tomato^34^. Therefore, we propose that auxin may mainly play a primary role in flavonol biosynthesis and that ethylene, ABA, and JA may also contribute to this process.

### *MdMYB8* regulates flavonol biosynthesis

*MdMYB8* (MD06G1217200) was selected from the DEGs on the basis of WGCNA as a putative regulator of flavonol biosynthesis. Through amino acid sequence alignments and a phylogenetic tree analysis, we found that it shared high homology with PbWER-like1, PbWER-like2, MdMYB62-like, AtMYB75, AtMYB90, AtMYB113, AtMYB114, and MdMYB10 (Fig. S6). AtMYB75, AtMYB90, AtMYB113, and AtMYB114 regulate the anthocyanin pathway and activate *UFGT*^35^ in *A. thaliana*. In apple, MdMYB10 has been reported to promote anthocyanin accumulation through the activation of two bHLH proteins, MdbHLH3 and MdbHLH33^10^. Our results suggest that MdMYB8 is involved in flavonoid biosynthesis (Figs. 3, 4, and 5) and that it binds to the *MdFLS* promoter to regulate flavonol biosynthesis (Figs. 5 and 6). Moreover, the overexpression of *MdMYB8* promoted flavonol biosynthesis, while RNAi knockdown of *MdMYB8* in transgenic ‘Orin’ apple calli resulted in a lower flavonol content. This phenomenon may be unique to the green fruit variety ‘Flame’, which lacks the anthocyanin regulatory network and therefore exhibits little or no anthocyanin accumulation.

In conclusion, we generated evidence that *MdMYB8* regulates flavonol biosynthesis and that auxin, ethylene, ABA, and JA may also be involved in this process. These studies provide a more detailed understanding of the flavonol regulatory network during crabapple fruit development and a new perspective for studying flavonol biosynthesis in anthocyanin-deficient plants.

## Materials and methods

### Plant materials and growth

*Malus* cv. ‘Flame’ fruit were harvested at five continuous developmental stages (35, 60, 95, 120, and 150 days after full bloom). Fruit peels with <1 mm of cortical tissue were frozen in liquid nitrogen for further HPLC analysis or RNA extraction.

### RNA-seq library preparation and sequencing

RNA library preparation and sequencing were performed as described previously^12^. Then, 1% agarose gels were used to visualize RNA degradation and contamination. A Nano Photometer^®^ spectrophotometer (IMPLEN, CA, USA) was used to detect the purity of RNA. The Qubit^®^ RNA Assay Kit and a Qubit^®^ 2.0 Fluorometer (Life Technologies, CA, USA) were used to detect the concentration of RNA. The RNA Nano 6000 Assay Kit was used to assess RNA integrity.

### Mapping to the apple genome and gene expression quantification

TopHat v2.0.12 was used to align clean reads to the apple genome^36,37^. HTSeq v0.6.1 was used to map read numbers to each gene^38^, and FPKM values were calculated from the gene length and read counts mapped to the genes^39^.

### Differential expression analysis

The DESeq R package (http://www.bioconductor.org/ packages/ release/ bioc/ html/ DESeq.html) was used to conduct differential expression analysis of three groups (three biological replicates per group)^40^. The false discovery rate was controlled by using the Benjamini and Hochberg approach to adjust the *P*-values^41^. Genes were considered to be differentially expressed according to a *P*-value < 0.05^42^.

### KEGG enrichment analysis of DEGs and identification of coexpression modules

The statistical enrichment of the DEGs in KEGG pathways was tested by KOBAS^43^. The R package WGCNA was used to identify modules of highly correlated genes based on the FPKM data^44^^,45^. We filtered the genes based on gene expression and variations by using the R package DCGL. The TOMsimilarity algorithm was employed to convert the adjacency matrix to a topological overlap (TO) matrix^46^. Modules whose eigengenes were highly correlated (correlation > 0.8) were merged^20^.

### Visualization of hub genes

The top 100 hub genes were calculated by eigengene-based connectivity, ranked by k (k_cor,i_^(q)^ = cor(xi, ^E(q)^) and ME (module eigengene). KOBAS 2.0 was used for gene annotation^44^.

### HPLC analysis and qRT-PCR analysis

HPLC analysis was performed as described previously^47^. Frozen apple peel samples (~0.8–1.0 g fresh weight) were incubated in 10 mL of extraction solution (methanol: water: formic acid: trifluoroacetic acid = 70: 27: 2: 1) at 4 °C in the dark for 72 h with shaking every 6 h. All samples were analyzed in biological triplicates (extracted from three different batches of fruit peels). qRT-PCR was performed as described previously^18,48^. An RNA Extraction Kit (Aidlab, Beijing, China) was used to extract total RNA from the apple peels according to the manufacturer’s instructions. SYBR Green qPCR Mix (TaKaRa, Ohtsu, Japan) and the Bio-Rad CFX96 Real-Time PCR System (BIO-RAD, USA) were used to detect the expression levels of flavonoid-related genes according to the manufacturers’ instructions.

### Y1H assay and transformation of apple calli

The Y1H assay was performed as described previously^44^. The coding DNA sequences of the 11 candidate hub genes were cloned into the *EcoR*I and *Xho*I sites of the pJG4-5 vector. The cells were selected on media lacking tryptophan and uracil, and positive colonies were spotted onto glucose plates (2%) containing X-gal at 28 °C for 2 days to confirm blue color generation. The transformation of apple calli was performed as described previously^17^. The full-length sequence of *MdMYB8* (MD06G1217200) was cloned into the pRI101-AN vector containing a green fluorescent protein (GFP) tag, followed by transformation into *A. tumefaciens* LBA4404^17^. All primers are listed in Supplementary Table S3.

### DPBA staining of flavonols

Flavonol contents in apple calli were observed by staining with DPBA^14^. Apple calli were stained with 0.25% (w/v) DPBA immediately postinfection and after 2 weeks of light treatment.

### Data analysis

The experimental data were analyzed using one-way ANOVA followed by Tukey’s multiple range test to compare differences between the experimental sites at *P* *<* 0.05. Origin 95, Microsoft Excel 2016 and IBM SPSS Statistics 22 were used for analysis.

### Accession numbers

Sequence data according to accession numbers from this article can be found in the apple genome database (http://www.rosaceae.org) or the National Center for Biotechnology Information (NCBI, https://www.ncbi.nlm.nih.gov), under the following accession numbers: *PAL* (MD01G1106900, MD04G1096200, MD07G1172700, MD12G1116700), *CHS* (MD04G1003300, MD13G1285100), *CHI* (MD01G1117800, MD01G1118100, and MD01G1118300, MD01G1167300, MD07G1186300, and MD07G1233400), *F3H* (MD02G1132200, MD15G1353800), *F3*′*H* (MD14G1210700), *DFR* (MD03G1214100, MD15G1024100, MD08G1103100, and MD11G1229100), *ANS* (MD03G1001100, MD06G1071600, and MD07G1222600), *UFGT* (MD09G1141700, MD07G1007400, and MD09G1141200), *FLS* (MD08G1121600), *LAR* (MD16G1048500, MD13G1046900), *ANR* (MD05G1335600, MD10G1311100, and MD01G1077800, MD01G1077900, and MD06G1103800, MD06G1103900), the auxin-responsive proteins (MD12G1097400, MD15G1014200, MD01G1227100, MD07G1297400, MD12G1113400, MD15G1014300, MD15G1060800, and MD16G1124300), auxin-induced proteins (MD16G1124300, MD10G1192900), ethylene-responsive transcription factor 2 (MD01G1177000), homeobox-leucine zipper protein HOX6 (MD01G1226600), WRKY transcription factor 6 (MD05G1349800), MYB transcription factor 8 (MD06G1217200), WRKY transcription factor 75 (MD13G1122100), ethylene-responsive transcription factor ERF115 (MD13G1130700), ethylene-responsive transcription factor 1B (MD13G1213100), ethylene-responsive transcription factor CRF2 (MD14G1120000), 40S ribosomal protein S7 (MD14G1227500), WRKY transcription factor 7 (MD15G1078200), and transcription factor bHLH135 (MD17G1049300).

## Supplementary information

Supplemental Figures and Tables
