# Supplementary material for: MdMYB8 is associated with flavonol biosynthesis via the activation of the MdFLS promoter in the fruits of Malus crabapple
Source: Hortic Res. 2020 Feb 1;7:19. doi: 10.1038/s41438-020-0238-z (PMC6994661; doi:10.1038/s41438-020-0238-z)
Supplement: Supplementary file 1 — Supplemental Figures and Tables [file 41438_2020_238_MOESM1_ESM.docx]

Supplemental Materials

**Fig. S1**


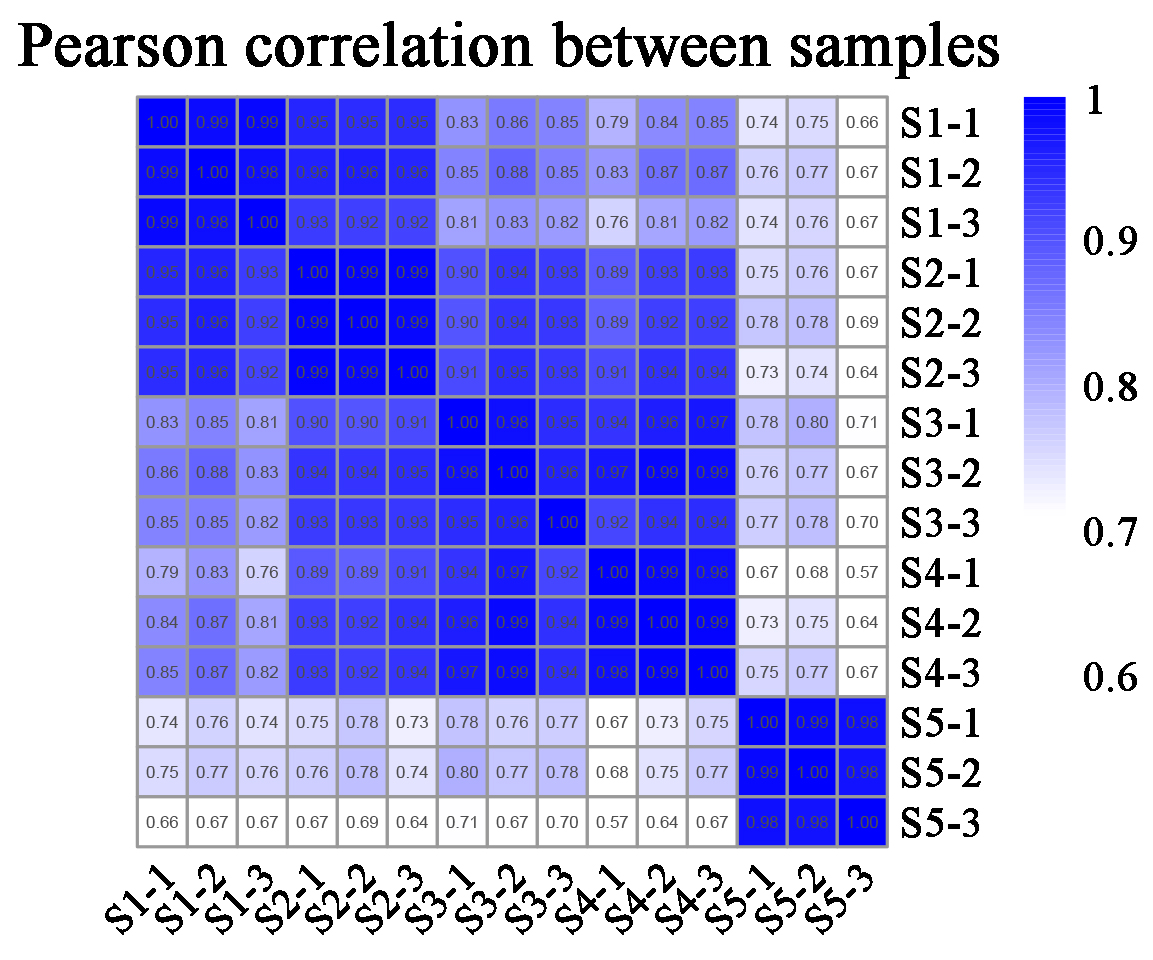


**Fig. S1.** Heat map showing the correlations between biological replicates. The PCC (Pearson correlation coefficient) values are quantitative indicators of relative expression levels of all genes in each sample.

**Fig. S2**





**Fig. S2.** The distribution of cleaned RNA-Seq reads mapped to the apple reference genome. ‘Exon’, ‘intron’ and ‘intergenic’ refer to the percentages of cleaned reads mapped to an exon intron or intergenic region, respectively, in the reference genome.

**Fig. S3**


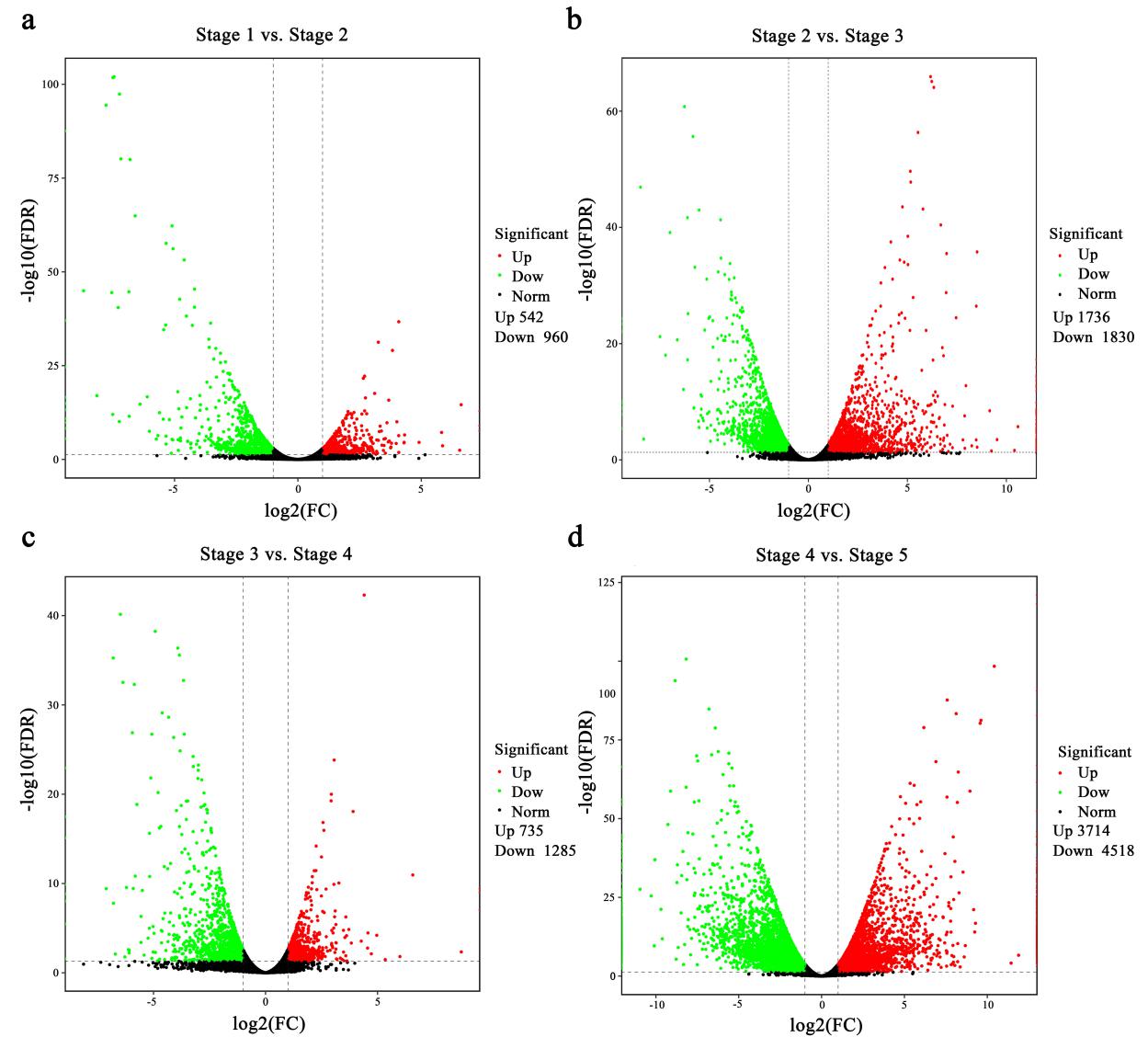


**Fig. S3.** Volcano plot visualizing the DEGs between two contiguous developmental stage. (a-d) Volcano plot of DEGs in Stage 1 vs. Stage 2 (a), Stage 2 vs. Stage 3 (b), Stage 3 vs. Stage 4 (c), Stage 4 vs. Stage 5 (d), respectively.

**Fig. S4**

**
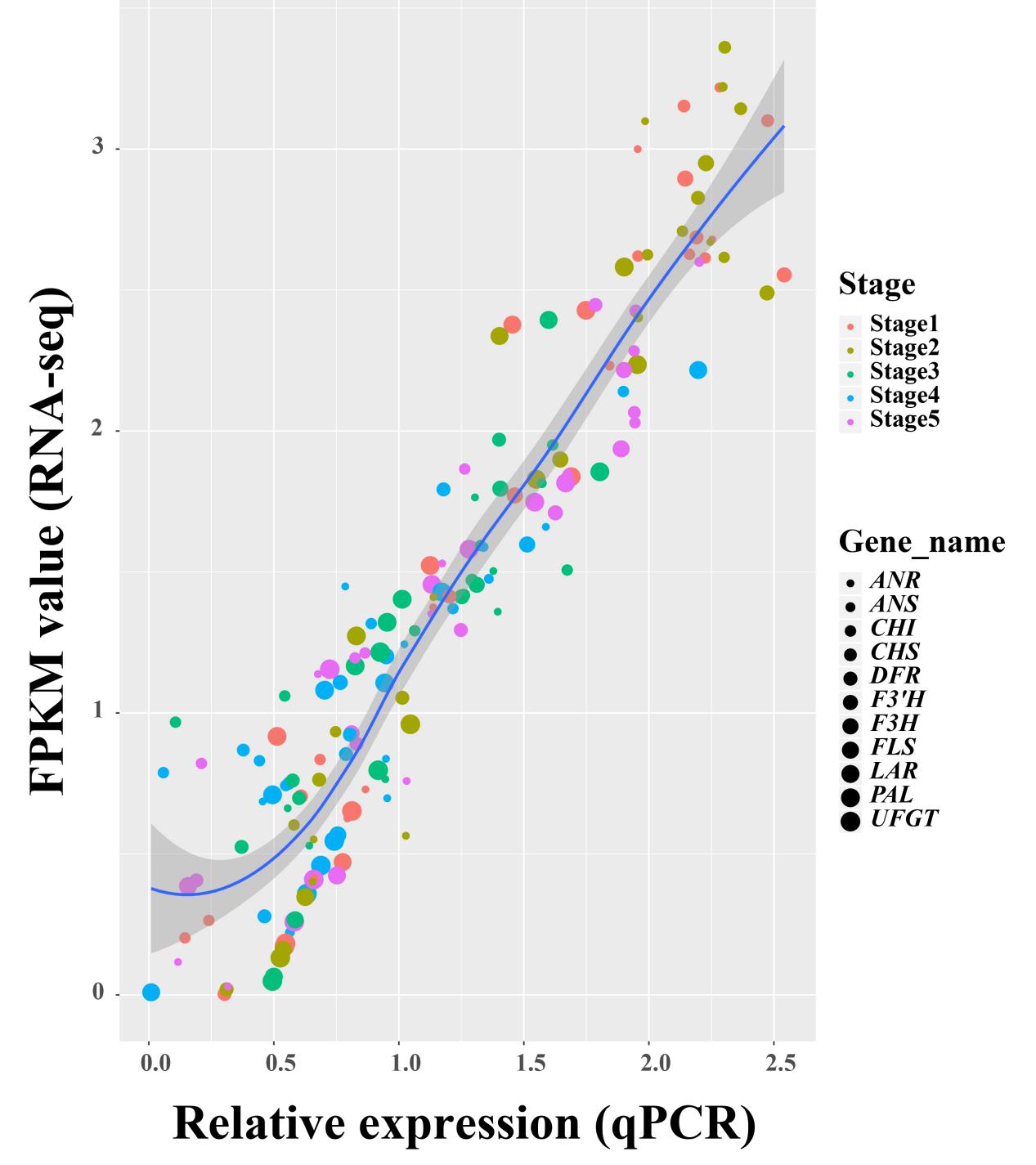
**

**Supplementary Fig. S4.** Scatter plot of gene expression data obtained through RNA-seq and qRT-PCR of flavonoid pathway genes in five fruit developmental stages.

**Fig. S5**


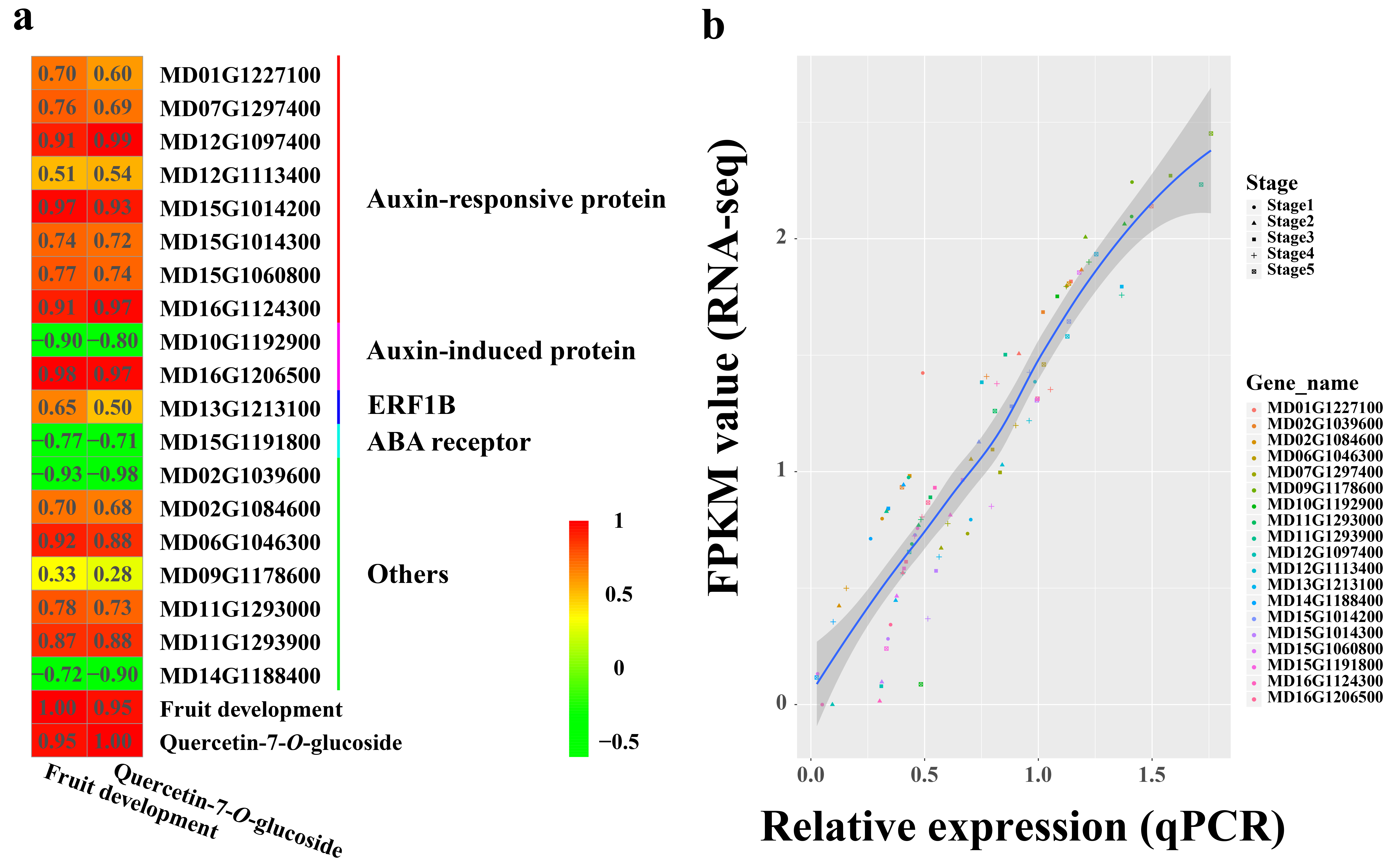


**Supplementary Fig. S5.** The expression analysis of ‘hormone signal transduction’ related genes during fruit development. (a) The correlation analysis between the expression level of ‘hormone signal transduction’ related genes with the accumulation of querectin-3-*O*-glucoside and the trend of fruit development. The expression trend of fruit development is set as the ideal analog data 1, 2, 3, 4, 5 in the S1, S2, S3, S4, S5, respectively. (b) Scatter plot of gene expression data obtained through RNA-seq and qRT-PCR of ‘hormone signal transduction’ related genes in five fruit developmental stages.

**Fig. S6**

**
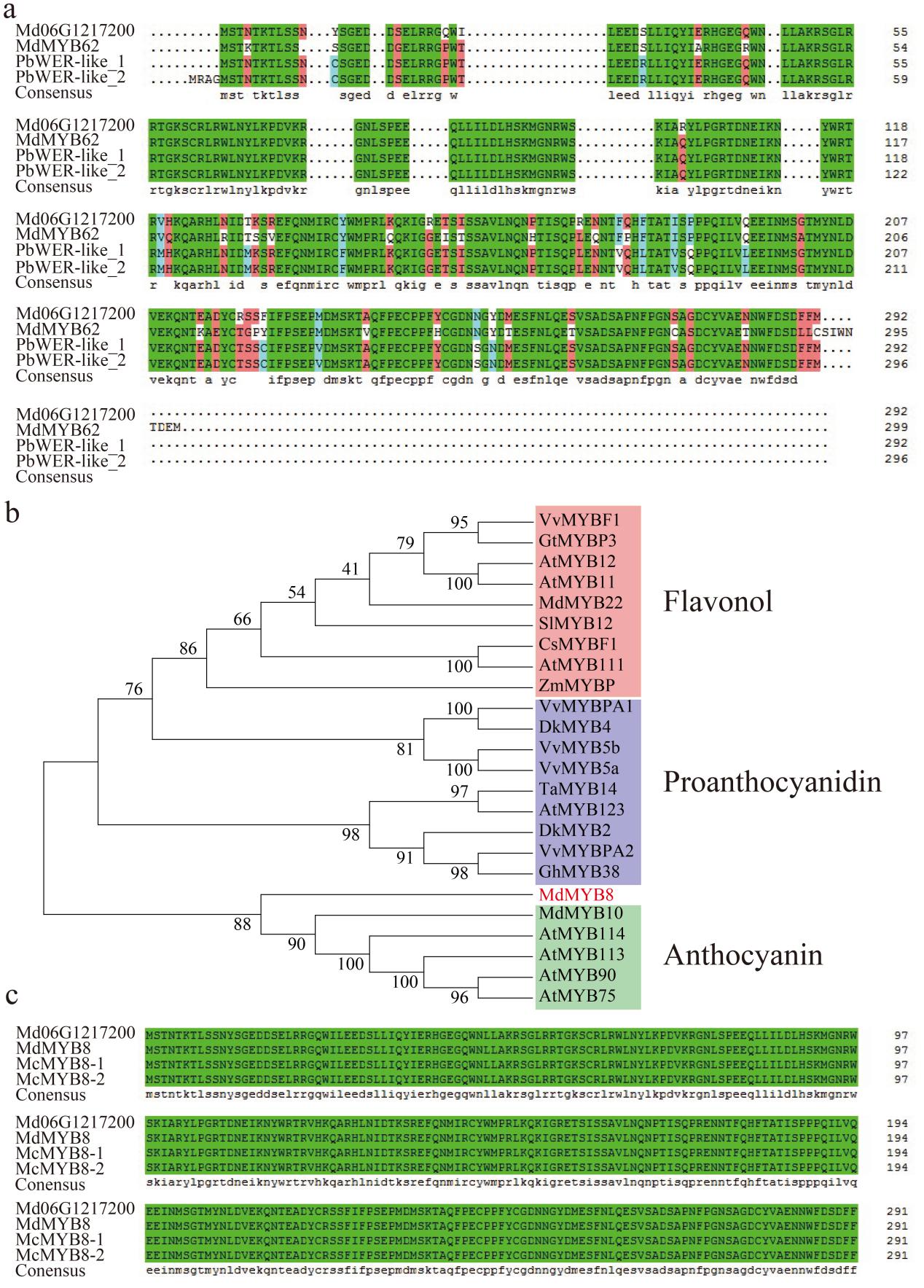
**

**Supplementary Fig. S6.** Multiple alignment of MdMYB8 (MD06G1217200) and a phylogenetic tree analysis with related MYB protein. (a) Alignment of the candidate hub gene *MdMYB8* (MD06G1217200) with amino acid sequences from other transcription factors. (b) Phylogenetic relationship of MdMYB8 (MD06G1217200) with other MYB transcription factors. GenBank accession numbers are as follows: VvMYBF1 (ACT88298), GtMYBP3 (BAM71801), AtMYB12 (NP_182268), AtMYB11 (NP_191820), MdMYB22 (AAZ20438), SlMYB12 (ACB46530), CsMYBF1 (AMH40451), AtMYB111 (AAK97396), ZmMYBP (P27898), VvMYBPA1 (CAJ90831), DkMYB4 (BAI49721), VvMYB5b (AAX51291), VvMYB5a (AAS68190), TaMYB14 (AFJ53053), AtMYB123 (CAC40021), DkMYB2 (BAI49719), VvMYBPA2 (ACK56131), GhMYB38 (AAK19618), MdMYB10 (ACQ45201), AtMYB114 (AEE34502), AtMYB113 (AEE34501), AtMYB90 (AAG42002), AtMYB75 (AAG42001). (c) Multiple alignment of MdMYB8 genes, including MdMYB8 (crabapple cultivar ‘Flame’), McMYB8-1 (crabapple cultivar ‘Royalty’), McMYB8-2 (crabapple cultivar ‘India magic’).

| **Table S1.** List of DEGs related to ‘Plant hormone signal transduction’ between S1 vs. S2, S2 vs. S3, S3 vs. S4 and S4 vs. S5. | | |
| --- | --- | --- |
| **Gene ID** | **NR_annotation** | **Types** |
| MD15G1417900 | PREDICTED: G-box-binding factor 4-like [*Malus* *domestica*] | Stage 1 vs. Stage 2 |
| MD10G1061400 | PREDICTED: indole-3-acetic acid-induced protein ARG7-like [*Malus* *domestica*] | Stage 1 vs. Stage 2 |
| MD12G1199000 | PREDICTED: transcription factor PIF3-like [*Malus* *domestica*] | Stage 1 vs. Stage 2 |
| MD15G1346400 | PREDICTED: two-component response regulator ORR9-like isoform X2 [*Malus* *domestica*] | Stage 1 vs. Stage 2 |
| MD05G1113300 | PREDICTED: auxin-induced protein 15A-like [*Malus* *domestica*] | Stage 1 vs. Stage 2 |
| MD15G1014200 | PREDICTED: auxin-induced protein 15A [*Malus* *domestica*] | Stage 1 vs. Stage 2 |
| MD17G1198300 | PREDICTED: auxin-responsive protein IAA1 [*Malus* *domestica*] | Stage 1 vs. Stage 2 |
| MD13G1209700 | PREDICTED: ethylene receptor 2-like [*Malus* *domestica*] | Stage 1 vs. Stage 2 |
| MD10G1287900 | auxin response factor 3 [*Malus* *domestica*] | Stage 1 vs. Stage 2 |
| MD11G1093100 | PREDICTED: probable protein phosphatase 2C 24 [*Malus* *domestica*] | Stage 1 vs. Stage 2 |
| MD15G1155800 | PREDICTED: two-component response regulator ARR12-like isoform X1 [*Malus* *domestica*] | Stage 1 vs. Stage 2 |
| MD05G1087300 | PREDICTED: cyclin-D3-1 [*Malus* *domestica*] | Stage 1 vs. Stage 2 |
| MD15G1195800 | PREDICTED: protein phosphatase 2C 56-like [*Malus* *domestica*] | Stage 1 vs. Stage 2 |
| MD16G1023300 | DELLA protein [*Malus* *domestica*] | Stage 1 vs. Stage 2 |
| MD02G1030300 | PREDICTED: EIN3-binding F-box protein 1-like [*Malus* *domestica*] | Stage 1 vs. Stage 2 |
| MD07G1291000 | PREDICTED: probable protein phosphatase 2C 51 [*Pyrus* x *bretschneideri*] | Stage 1 vs. Stage 2 |
| MD01G1139200 | PREDICTED: protein phosphatase 2C 37-like [*Pyrus* x *bretschneideri*] | Stage 1 vs. Stage 2 |
| MD08G1150200 | EIN3-binding F-box protein 1-like [*Malus* *domestica*] | Stage 1 vs. Stage 2 |
| MD05G1052100 | PREDICTED: auxin-induced protein 15A-like [*Malus* *domestica*] | Stage 1 vs. Stage 2 |
| MD15G1171800 | PREDICTED: EIN3-binding F-box protein 1-like [*Malus* *domestica*] | Stage 1 vs. Stage 2 |
| MD05G1113400 | hypothetical protein PRUPE_8G157900 [*Prunus persica*] | Stage 1 vs. Stage 2 |
| MD13G1222200 | ARF domain class transcription factor [*Malus* *domestica*] | Stage 1 vs. Stage 2 |
| MD15G1125200 | PREDICTED: EIN3-binding F-box protein 1-like [*Pyrus* x *bretschneideri*] | Stage 1 vs. Stage 2 |
| MD15G1081800 | PREDICTED: ABSCISIC ACID-INSENSITIVE 5-like protein 5 isoform X1 [*Pyrus* x *bretschneideri*] | Stage 1 vs. Stage 2 |
| MD15G1413000 | PREDICTED: BRASSINOSTEROID INSENSITIVE 1-associated receptor kinase 1-like [*Malus* *domestica*] | Stage 1 vs. Stage 2 |
| MD00G1033700 | PREDICTED: auxin-induced protein 6B-like [*Malus* *domestica*] | Stage 1 vs. Stage 2 |
| MD07G1203700 | PREDICTED: protein phosphatase 2C 37 [*Malus* *domestica*] | Stage 1 vs. Stage 2 |
| MD05G1052000 | PREDICTED: auxin-induced protein 15A-like [*Malus* *domestica*] | Stage 1 vs. Stage 2 |
| MD16G1017900 | PREDICTED: two-component response regulator ORR21-like [*Malus* *domestica*] | Stage 1 vs. Stage 2 |
| MD16G1206700 | PREDICTED: auxin-responsive protein IAA14-like [*Malus* *domestica*] | Stage 1 vs. Stage 2 |
| MD10G1060900 | PREDICTED: auxin-induced protein 15A-like [*Pyrus* x *bretschneideri*] | Stage 1 vs. Stage 2 |
| MD02G1105900 | PREDICTED: protein TIFY 3-like [*Malus* *domestica*] | Stage 1 vs. Stage 2 |
| MD05G1051700 | PREDICTED: auxin-induced protein 15A-like [*Malus* *domestica*] | Stage 1 vs. Stage 2 |
| MD12G1226800 | PREDICTED: histidine-containing phosphotransfer protein 1 [*Malus* *domestica*] | Stage 1 vs. Stage 2 |
| MD05G1325800 | PREDICTED: protein TRANSPORT INHIBITOR RESPONSE 1-like [*Malus* *domestica*] | Stage 1 vs. Stage 2 |
| MD12G1162400 | PREDICTED: auxin transporter-like protein 2 [*Malus* *domestica*] | Stage 2 vs. Stage 3 |
| MD11G1293900 | histidine-containing phosphotransfer 3a [*Malus* *domestica*] | Stage 2 vs. Stage 3 |
| MD15G1346400 | PREDICTED: two-component response regulator ORR9-like isoform X2 [*Malus* *domestica*] | Stage 2 vs. Stage 3 |
| MD05G1113300 | PREDICTED: auxin-induced protein 15A-like [*Malus* *domestica*] | Stage 2 vs. Stage 3 |
| MD17G1081000 | PREDICTED: jasmonic acid-amido synthetase JAR1 [*Pyrus* x *bretschneideri*] | Stage 2 vs. Stage 3 |
| MD07G1117400 | PREDICTED: auxin-responsive protein SAUR36-like [*Malus* *domestica*] | Stage 2 vs. Stage 3 |
| MD15G1169100 | PREDICTED: auxin-responsive protein IAA20-like [*Malus* *domestica*] | Stage 2 vs. Stage 3 |
| MD05G1092300 | PREDICTED: probable indole-3-acetic acid-amido synthetase GH3.1 [*Malus* *domestica*] | Stage 2 vs. Stage 3 |
| MD06G1182400 | PREDICTED: two-component response regulator ARR5-like [*Malus* *domestica*] | Stage 2 vs. Stage 3 |
| MD13G1213100 | ethylene-responsive transcription factor 1B-like [*Malus* *domestica*] | Stage 2 vs. Stage 3 |
| MD10G1059600 | PREDICTED: auxin-induced protein 15A-like [*Malus* *domestica*] | Stage 2 vs. Stage 3 |
| MD11G1299000 | PREDICTED: protein TRANSPORT INHIBITOR RESPONSE 1 [*Malus* *domestica*] | Stage 2 vs. Stage 3 |
| MD09G1216100 | auxin-responsive Aux/IAA protein [*Pyrus* *pyrifolia*] | Stage 2 vs. Stage 3 |
| MD13G1209700 | PREDICTED: ethylene receptor 2-like [*Malus* *domestica*] | Stage 2 vs. Stage 3 |
| MD05G1198700 | PREDICTED: ethylene-responsive transcription factor 1B-like [*Pyrus* x *bretschneideri*] | Stage 2 vs. Stage 3 |
| MD15G1360600 | PREDICTED: transcription factor TGA7 [*Malus* *domestica*] | Stage 2 vs. Stage 3 |
| MD07G1147700 | PREDICTED: abscisic acid receptor PYL9-like [*Malus* *domestica*] | Stage 2 vs. Stage 3 |
| MD05G1087300 | PREDICTED: cyclin-D3-1 [*Malus* *domestica*] | Stage 2 vs. Stage 3 |
| MD04G1212400 | gibberellin receptor GID1C-like [*Malus* *domestica*] | Stage 2 vs. Stage 3 |
| MD10G1059700 | PREDICTED: auxin-responsive protein SAUR21-like [*Malus* *domestica*] | Stage 2 vs. Stage 3 |
| MD05G1052400 | PREDICTED: indole-3-acetic acid-induced protein ARG7-like [*Pyrus* x *bretschneideri*] | Stage 2 vs. Stage 3 |
| MD07G1291000 | PREDICTED: probable protein phosphatase 2C 51 [*Pyrus* x *bretschneideri*] | Stage 2 vs. Stage 3 |
| MD11G1306200 | ethylene receptor [*Malus* *domestica*] | Stage 2 vs. Stage 3 |
| MD03G1273300 | uncharacterized protein LOC103432477 [*Malus* *domestica*] | Stage 2 vs. Stage 3 |
| MD01G1139200 | PREDICTED: protein phosphatase 2C 37-like [*Pyrus* x *bretschneideri*] | Stage 2 vs. Stage 3 |
| MD15G1075800 | PREDICTED: F-box protein GID2-like [*Malus* *domestica*] | Stage 2 vs. Stage 3 |
| MD02G1084600 | PREDICTED: protein phosphatase 2C 77-like [*Malus* *domestica*] | Stage 2 vs. Stage 3 |
| MD10G1059800 | PREDICTED: auxin-responsive protein SAUR21-like [*Malus* *domestica*] | Stage 2 vs. Stage 3 |
| MD15G1171800 | PREDICTED: EIN3-binding F-box protein 1-like [*Malus* *domestica*] | Stage 2 vs. Stage 3 |
| MD12G1245100 | PREDICTED: protein EIN4 [*Malus* *domestica*] | Stage 2 vs. Stage 3 |
| MD06G1001100 | ethylene receptor [*Malus* *domestica*] | Stage 2 vs. Stage 3 |
| MD13G1017000 | PREDICTED: histidine kinase 4 [*Malus* *domestica*] | Stage 2 vs. Stage 3 |
| MD16G1216900 | PREDICTED: ethylene-responsive transcription factor 1B-like [*Malus* *domestica*] | Stage 2 vs. Stage 3 |
| MD05G1109100 | PREDICTED: pathogenesis-related protein 1-like [*Malus* *domestica*] | Stage 2 vs. Stage 3 |
| MD14G1086100 | PREDICTED: two-component response regulator ORR10-like [*Pyrus* x *bretschneideri*] | Stage 2 vs. Stage 3 |
| MD08G1091700 | PREDICTED: uncharacterized protein LOC103441251 [*Malus* *domestica*] | Stage 2 vs. Stage 3 |
| MD10G1060100 | PREDICTED: auxin-induced protein 15A-like [*Malus* *domestica*] | Stage 2 vs. Stage 3 |
| MD02G1205700 | PREDICTED: auxin-responsive protein SAUR36-like [*Malus* *domestica*] | Stage 2 vs. Stage 3 |
| MD05G1052200 | PREDICTED: auxin-induced protein 15A-like [*Pyrus* x *bretschneideri*] | Stage 2 vs. Stage 3 |
| MD12G1093500 | PREDICTED: two-component response regulator ORR10 [*Malus* *domestica*] | Stage 2 vs. Stage 3 |
| MD05G1223400 | PREDICTED: auxin-responsive protein SAUR71-like [*Malus* *domestica*] | Stage 2 vs. Stage 3 |
| MD07G1215900 | PREDICTED: auxin transporter-like protein 2 [*Malus* *domestica*] | Stage 2 vs. Stage 3 |
| MD17G1189100 | PREDICTED: LOW QUALITY PROTEIN: auxin-responsive protein IAA21-like [*Malus* *domestica*] | Stage 2 vs. Stage 3 |
| MD10G1060600 | PREDICTED: auxin-induced protein 15A-like [*Malus* *domestica*] | Stage 2 vs. Stage 3 |
| MD10G1059200 | PREDICTED: auxin-induced protein 15A-like [*Malus* *domestica*] | Stage 2 vs. Stage 3 |
| MD08G1161200 | PREDICTED: two-component response regulator ORR10-like [*Malus* *domestica*] | Stage 2 vs. Stage 3 |
| MD15G1413000 | PREDICTED: BRASSINOSTEROID INSENSITIVE 1-associated receptor kinase 1-like [*Malus* *domestica*] | Stage 2 vs. Stage 3 |
| MD10G1192900 | PREDICTED: auxin-induced protein AUX28 [*Malus* *domestica*] | Stage 2 vs. Stage 3 |
| MD00G1033700 | PREDICTED: auxin-induced protein 6B-like [*Malus* *domestica*] | Stage 2 vs. Stage 3 |
| MD15G1225800 | PREDICTED: protein TIFY 3B-like [*Malus* *domestica*] | Stage 2 vs. Stage 3 |
| MD08G1111200 | PREDICTED: auxin-responsive protein IAA2-like [*Malus* *domestica*] | Stage 2 vs. Stage 3 |
| MD15G1060800 | PREDICTED: abscisic acid receptor PYL2-like [*Malus* *domestica*] | Stage 2 vs. Stage 3 |
| MD12G1061900 | PREDICTED: transcription factor HBP-1b(c38)-like [*Malus* *domestica*] | Stage 2 vs. Stage 3 |
| MD13G1085200 | PREDICTED: two-component response regulator ARR5-like [*Malus* *domestica*] | Stage 2 vs. Stage 3 |
| MD10G1059500 | PREDICTED: indole-3-acetic acid-induced protein ARG7-like, partial [*Malus* *domestica*] | Stage 2 vs. Stage 3 |
| MD00G1165200 | PREDICTED: jasmonic acid-amido synthetase JAR1 isoform X1 [*Malus* *domestica*] | Stage 2 vs. Stage 3 |
| MD11G1085000 | PREDICTED: probable serine/threonine-protein kinase At5g41260 [*Malus* *domestica*] | Stage 2 vs. Stage 3 |
| MD16G1212500 | PREDICTED: ethylene receptor 2-like [*Malus* *domestica*] | Stage 2 vs. Stage 3 |
| MD09G1208000 | PREDICTED: auxin-responsive protein IAA21-like isoform X2 [*Malus* *domestica*] | Stage 2 vs. Stage 3 |
| MD09G1091000 | PREDICTED: jasmonic acid-amido synthetase JAR1 isoform X1 [*Malus* *domestica*] | Stage 2 vs. Stage 3 |
| MD12G1227200 | PREDICTED: gibberellin receptor GID1C-like [*Malus* *domestica*] | Stage 2 vs. Stage 3 |
| MD07G1203700 | PREDICTED: protein phosphatase 2C 37 [*Malus* *domestica*] | Stage 2 vs. Stage 3 |
| MD13G1268900 | PREDICTED: xyloglucan endotransglucosylase/hydrolase protein 22-like [*Malus* *domestica*] | Stage 2 vs. Stage 3 |
| MD16G1124300 | PREDICTED: auxin-responsive protein SAUR32-like [*Malus* *domestica*] | Stage 2 vs. Stage 3 |
| MD13G1120900 | histidine kinase 3-like [*Malus* *domestica*] | Stage 2 vs. Stage 3 |
| MD08G1014900 | PREDICTED: auxin-induced protein 15A-like [*Malus* *domestica*] | Stage 2 vs. Stage 3 |
| MD05G1092900 | PREDICTED: probable indole-3-acetic acid-amido synthetase GH3.1 [*Malus* *domestica*] | Stage 2 vs. Stage 3 |
| MD16G1206700 | PREDICTED: auxin-responsive protein IAA14-like [*Malus* *domestica*] | Stage 2 vs. Stage 3 |
| MD02G1100000 | PREDICTED: uncharacterized protein LOC103453925 isoform X2 [*Malus* *domestica*] | Stage 2 vs. Stage 3 |
| MD04G1149300 | PREDICTED: auxin transporter-like protein 2 isoform X1 [*Pyrus* x *bretschneideri*] | Stage 2 vs. Stage 3 |
| MD09G1136800 | PREDICTED: protein TIFY 5A-like [*Pyrus* x *bretschneideri*] | Stage 2 vs. Stage 3 |
| MD02G1105900 | PREDICTED: protein TIFY 3-like [*Malus* *domestica*] | Stage 2 vs. Stage 3 |
| MD05G1051700 | PREDICTED: auxin-induced protein 15A-like [*Malus* *domestica*] | Stage 2 vs. Stage 3 |
| MD10G1184800 | PREDICTED: ethylene-responsive transcription factor 1B-like [*Malus* *domestica*] | Stage 2 vs. Stage 3 |
| MD17G1133300 | NPR6 [*Malus* *domestica*] | Stage 2 vs. Stage 3 |
| MD16G1084400 | PREDICTED: two-component response regulator ARR5 [*Malus* *domestica*] | Stage 2 vs. Stage 3 |
| MD10G1061400 | PREDICTED: indole-3-acetic acid-induced protein ARG7-like [*Malus* *domestica*] | Stage 3 vs. Stage 4 |
| MD12G1198900 | transcription factor PIF3-like [*Malus* *domestica*] | Stage 3 vs. Stage 4 |
| MD01G1227100 | PREDICTED: auxin-responsive protein SAUR32-like [*Malus* *domestica*] | Stage 3 vs. Stage 4 |
| MD17G1081000 | PREDICTED: jasmonic acid-amido synthetase JAR1 [*Pyrus* x *bretschneideri*] | Stage 3 vs. Stage 4 |
| MD07G1117400 | PREDICTED: auxin-responsive protein SAUR36-like [*Malus* *domestica*] | Stage 3 vs. Stage 4 |
| MD06G1182400 | PREDICTED: two-component response regulator ARR5-like [*Malus* *domestica*] | Stage 3 vs. Stage 4 |
| MD02G1057200 | auxin-responsive protein IAA27 [*Malus* *domestica*] | Stage 3 vs. Stage 4 |
| MD13G1213100 | ethylene-responsive transcription factor 1B-like [*Malus* *domestica*] | Stage 3 vs. Stage 4 |
| MD10G1059600 | PREDICTED: auxin-induced protein 15A-like [*Malus* *domestica*] | Stage 3 vs. Stage 4 |
| MD09G1216100 | auxin-responsive Aux/IAA protein [*Pyrus* *pyrifolia*] | Stage 3 vs. Stage 4 |
| MD17G1164400 | PREDICTED: protein TIFY 10B-like [*Malus* *domestica*] | Stage 3 vs. Stage 4 |
| MD05G1198700 | PREDICTED: ethylene-responsive transcription factor 1B-like [*Pyrus* x *bretschneideri*] | Stage 3 vs. Stage 4 |
| MD14G1021600 | PREDICTED: protein ABSCISIC ACID-INSENSITIVE 5-like isoform X1 [*Pyrus* x *bretschneideri*] | Stage 3 vs. Stage 4 |
| MD15G1195800 | PREDICTED: protein phosphatase 2C 56-like [*Malus* *domestica*] | Stage 3 vs. Stage 4 |
| MD09G1146000 | PREDICTED: transcription factor PIF4-like [*Malus* *domestica*] | Stage 3 vs. Stage 4 |
| MD03G1273300 | uncharacterized protein LOC103432477 [*Malus* *domestica*] | Stage 3 vs. Stage 4 |
| MD15G1075800 | PREDICTED: F-box protein GID2-like [*Malus* *domestica*] | Stage 3 vs. Stage 4 |
| MD02G1027600 | PREDICTED: auxin-responsive protein IAA29-like [*Malus* *domestica*] | Stage 3 vs. Stage 4 |
| MD05G1052100 | PREDICTED: auxin-induced protein 15A-like [*Malus* *domestica*] | Stage 3 vs. Stage 4 |
| MD14G1188400 | PREDICTED: two-component response regulator ARR5-like [*Malus* *domestica*] | Stage 3 vs. Stage 4 |
| MD02G1084600 | PREDICTED: protein phosphatase 2C 77-like [*Malus* *domestica*] | Stage 3 vs. Stage 4 |
| MD10G1059800 | PREDICTED: auxin-responsive protein SAUR21-like [*Malus* *domestica*] | Stage 3 vs. Stage 4 |
| MD12G1245100 | PREDICTED: protein EIN4 [*Malus* *domestica*] | Stage 3 vs. Stage 4 |
| MD02G1096100 | PREDICTED: protein TIFY 10A [*Malus* *domestica*] | Stage 3 vs. Stage 4 |
| MD16G1216900 | PREDICTED: ethylene-responsive transcription factor 1B-like [*Malus* *domestica*] | Stage 3 vs. Stage 4 |
| MD08G1091700 | PREDICTED: uncharacterized protein LOC103441251 [*Malus* *domestica*] | Stage 3 vs. Stage 4 |
| MD09G1178600 | PREDICTED: protein TIFY 6b-like [*Malus* *domestica*] | Stage 3 vs. Stage 4 |
| MD02G1205700 | PREDICTED: auxin-responsive protein SAUR36-like [*Malus* *domestica*] | Stage 3 vs. Stage 4 |
| MD00G1033700 | PREDICTED: auxin-induced protein 6B-like [*Malus* *domestica*] | Stage 3 vs. Stage 4 |
| MD01G1021600 | PREDICTED: transcription factor TGA4-like isoform X4 [*Malus* *domestica*] | Stage 3 vs. Stage 4 |
| MD13G1127100 | PREDICTED: protein TIFY 9 [*Malus* *domestica*] | Stage 3 vs. Stage 4 |
| MD16G1274200 | PREDICTED: transcription factor MYC2-like [*Malus* *domestica*] | Stage 3 vs. Stage 4 |
| MD11G1085000 | PREDICTED: probable serine/threonine-protein kinase At5g41260 [*Malus* *domestica*] | Stage 3 vs. Stage 4 |
| MD16G1212500 | PREDICTED: ethylene receptor 2-like [*Malus* *domestica*] | Stage 3 vs. Stage 4 |
| MD09G1208000 | PREDICTED: auxin-responsive protein IAA21-like isoform X2 [*Malus* *domestica*] | Stage 3 vs. Stage 4 |
| MD05G1205800 | PREDICTED: auxin-induced protein AUX28-like [*Malus* *domestica*] | Stage 3 vs. Stage 4 |
| MD10G1236700 | NPR3 [*Malus* *domestica*] | Stage 3 vs. Stage 4 |
| MD09G1146600 | NPR5a [*Malus* *domestica*] | Stage 3 vs. Stage 4 |
| MD15G1090600 | auxin [*Malus* zumi] | Stage 3 vs. Stage 4 |
| MD17G1198100 | PREDICTED: auxin-induced protein IAA6 [*Malus* *domestica*] | Stage 3 vs. Stage 4 |
| MD13G1204700 | PREDICTED: auxin-induced protein 22D [*Malus* *domestica*] | Stage 3 vs. Stage 4 |
| MD16G1124300 | PREDICTED: auxin-responsive protein SAUR32-like [*Malus* *domestica*] | Stage 3 vs. Stage 4 |
| MD16G1127400 | PREDICTED: protein TIFY 9-like [*Pyrus* x *bretschneideri*] | Stage 3 vs. Stage 4 |
| MD10G1303700 | PREDICTED: protein TRANSPORT INHIBITOR RESPONSE 1-like [*Malus* *domestica*] | Stage 3 vs. Stage 4 |
| MD15G1220400 | PREDICTED: protein TIFY 10A-like [*Malus* *domestica*] | Stage 3 vs. Stage 4 |
| MD09G1136800 | PREDICTED: protein TIFY 5A-like [*Pyrus* x *bretschneideri*] | Stage 3 vs. Stage 4 |
| MD10G1184800 | PREDICTED: ethylene-responsive transcription factor 1B-like [*Malus* *domestica*] | Stage 3 vs. Stage 4 |
| MD11G1296000 | uncharacterized protein LOC103449094 [*Malus* *domestica*] | Stage 4 vs. Stage 5 |
| MD01G1035000 | sucrose non-fermenting-1-related protein kinase 2.12 [*Malus* prunifolia] | Stage 4 vs. Stage 5 |
| MD12G1162400 | PREDICTED: auxin transporter-like protein 2 [*Malus* *domestica*] | Stage 4 vs. Stage 5 |
| MD01G1056900 | probable serine/threonine-protein kinase At5g41260 isoform X1 [*Prunus avium*] | Stage 4 vs. Stage 5 |
| MD11G1293900 | histidine-containing phosphotransfer 3a [*Malus* *domestica*] | Stage 4 vs. Stage 5 |
| MD15G1321000 | serine/threonine-protein kinase SAPK2-like [*Malus* *domestica*] | Stage 4 vs. Stage 5 |
| MD01G1227100 | PREDICTED: auxin-responsive protein SAUR32-like [*Malus* *domestica*] | Stage 4 vs. Stage 5 |
| MD08G1035400 | PREDICTED: protein BRASSINAZOLE-RESISTANT 1 [*Malus* *domestica*] | Stage 4 vs. Stage 5 |
| MD05G1113300 | PREDICTED: auxin-induced protein 15A-like [*Malus* *domestica*] | Stage 4 vs. Stage 5 |
| MD07G1117400 | PREDICTED: auxin-responsive protein SAUR36-like [*Malus* *domestica*] | Stage 4 vs. Stage 5 |
| MD15G1169100 | PREDICTED: auxin-responsive protein IAA20-like [*Malus* *domestica*] | Stage 4 vs. Stage 5 |
| MD16G1267300 | xyloglucan endotransglucosylase/hydrolase 8 [*Malus* *domestica*] | Stage 4 vs. Stage 5 |
| MD15G1222900 | PREDICTED: uncharacterized protein LOC103400902 isoform X2 [*Malus* *domestica*] | Stage 4 vs. Stage 5 |
| MD02G1057200 | auxin-responsive protein IAA27 [*Malus* *domestica*] | Stage 4 vs. Stage 5 |
| MD04G1225100 | auxin-responsive protein IAA16 [*Malus* *domestica*] | Stage 4 vs. Stage 5 |
| MD13G1213100 | ethylene-responsive transcription factor 1B-like [*Malus* *domestica*] | Stage 4 vs. Stage 5 |
| MD10G1059600 | PREDICTED: auxin-induced protein 15A-like [*Malus* *domestica*] | Stage 4 vs. Stage 5 |
| MD09G1216100 | auxin-responsive Aux/IAA protein [*Pyrus* *pyrifolia*] | Stage 4 vs. Stage 5 |
| MD10G1287900 | auxin response factor 3 [*Malus* *domestica*] | Stage 4 vs. Stage 5 |
| MD10G1176400 | PREDICTED: auxin-responsive protein IAA11-like [*Malus* *domestica*] | Stage 4 vs. Stage 5 |
| MD11G1093100 | PREDICTED: probable protein phosphatase 2C 24 [*Malus* *domestica*] | Stage 4 vs. Stage 5 |
| MD09G1202300 | auxin-responsive protein IAA26-like [*Malus* *domestica*] | Stage 4 vs. Stage 5 |
| MD15G1077100 | PREDICTED: cyclin-D3-1-like [*Malus* *domestica*] | Stage 4 vs. Stage 5 |
| MD07G1147700 | PREDICTED: abscisic acid receptor PYL9-like [*Malus* *domestica*] | Stage 4 vs. Stage 5 |
| MD11G1293000 | PREDICTED: probable indole-3-acetic acid-amido synthetase GH3.6 [*Malus* *domestica*] | Stage 4 vs. Stage 5 |
| MD02G1039600 | PREDICTED: LOW QUALITY PROTEIN: DELLA protein GAI [*Malus* *domestica*] | Stage 4 vs. Stage 5 |
| MD05G1087300 | PREDICTED: cyclin-D3-1 [*Malus* *domestica*] | Stage 4 vs. Stage 5 |
| MD14G1021600 | PREDICTED: protein ABSCISIC ACID-INSENSITIVE 5-like isoform X1 [*Pyrus* x *bretschneideri*] | Stage 4 vs. Stage 5 |
| MD10G1059700 | PREDICTED: auxin-responsive protein SAUR21-like [*Malus* *domestica*] | Stage 4 vs. Stage 5 |
| MD16G1023300 | DELLA protein [*Malus* *domestica*] | Stage 4 vs. Stage 5 |
| MD13G1205000 | PREDICTED: auxin-responsive protein IAA14-like [*Pyrus* x *bretschneideri*] | Stage 4 vs. Stage 5 |
| MD12G1241800 | PREDICTED: auxin-responsive protein IAA16 [*Pyrus* x *bretschneideri*] | Stage 4 vs. Stage 5 |
| MD16G1046000 | PREDICTED: transcription factor HBP-1b(c38) [*Malus* *domestica*] | Stage 4 vs. Stage 5 |
| MD09G1264800 | DELLA protein GAI [*Malus* *domestica*] | Stage 4 vs. Stage 5 |
| MD07G1291000 | PREDICTED: probable protein phosphatase 2C 51 [*Pyrus* x *bretschneideri*] | Stage 4 vs. Stage 5 |
| MD08G1207300 | auxin-responsive protein IAA8-like [*Malus* *domestica*] | Stage 4 vs. Stage 5 |
| MD09G1146000 | PREDICTED: transcription factor PIF4-like [*Malus* *domestica*] | Stage 4 vs. Stage 5 |
| MD15G1191800 | PREDICTED: auxin-responsive protein IAA27-like [*Pyrus* x *bretschneideri*] | Stage 4 vs. Stage 5 |
| MD08G1059000 | PREDICTED: two-component response regulator ARR12 [*Malus* *domestica*] | Stage 4 vs. Stage 5 |
| MD03G1273300 | uncharacterized protein LOC103432477 [*Malus* *domestica*] | Stage 4 vs. Stage 5 |
| MD15G1075800 | PREDICTED: F-box protein GID2-like [*Malus* *domestica*] | Stage 4 vs. Stage 5 |
| MD10G1061100 | PREDICTED: indole-3-acetic acid-induced protein ARG7-like [*Malus* *domestica*] | Stage 4 vs. Stage 5 |
| MD05G1052100 | PREDICTED: auxin-induced protein 15A-like [*Malus* *domestica*] | Stage 4 vs. Stage 5 |
| MD01G1088500 | PREDICTED: probable serine/threonine-protein kinase At4g35230 [*Pyrus* x *bretschneideri*] | Stage 4 vs. Stage 5 |
| MD02G1084600 | PREDICTED: protein phosphatase 2C 77-like [*Malus* *domestica*] | Stage 4 vs. Stage 5 |
| MD10G1059800 | PREDICTED: auxin-responsive protein SAUR21-like [*Malus* *domestica*] | Stage 4 vs. Stage 5 |
| MD11G1160500 | PREDICTED: histidine-containing phosphotransfer protein 2-like [*Malus* *domestica*] | Stage 4 vs. Stage 5 |
| MD13G1022100 | spur-type DELLA protein [*Malus* *domestica*] | Stage 4 vs. Stage 5 |
| MD17G1265100 | PREDICTED: shaggy-related protein kinase eta isoform X1 [*Malus* *domestica*] | Stage 4 vs. Stage 5 |
| MD08G1015500 | PREDICTED: auxin response factor 5-like [*Malus* *domestica*] | Stage 4 vs. Stage 5 |
| *Malus*_*domestica*_newGene_8696 | PREDICTED: auxin response factor 1 [*Pyrus* x *bretschneideri*] | Stage 4 vs. Stage 5 |
| MD06G1001100 | ethylene receptor [*Malus* *domestica*] | Stage 4 vs. Stage 5 |
| MD15G1330400 | PREDICTED: transcription factor TGA4-like [*Malus* *domestica*] | Stage 4 vs. Stage 5 |
| MD14G1131900 | PREDICTED: auxin response factor 9 [*Malus* *domestica*] | Stage 4 vs. Stage 5 |
| MD17G1275100 | hypothetical protein EUTSA_v10018818mg [*Eutrema salsugineum*] | Stage 4 vs. Stage 5 |
| MD02G1096100 | PREDICTED: protein TIFY 10A [*Malus* *domestica*] | Stage 4 vs. Stage 5 |
| MD16G1216900 | PREDICTED: ethylene-responsive transcription factor 1B-like [*Malus* *domestica*] | Stage 4 vs. Stage 5 |
| MD10G1060800 | PREDICTED: indole-3-acetic acid-induced protein ARG7-like [*Malus* *domestica*] | Stage 4 vs. Stage 5 |
| MD05G1027000 | PREDICTED: G-box-binding factor 4-like [*Pyrus* x *bretschneideri*] | Stage 4 vs. Stage 5 |
| MD05G1109100 | PREDICTED: pathogenesis-related protein 1-like [*Malus* *domestica*] | Stage 4 vs. Stage 5 |
| MD08G1091700 | PREDICTED: uncharacterized protein LOC103441251 [*Malus* *domestica*] | Stage 4 vs. Stage 5 |
| MD14G1152100 | PREDICTED: auxin-responsive protein SAUR71-like [*Pyrus* x *bretschneideri*] | Stage 4 vs. Stage 5 |
| MD09G1178600 | PREDICTED: protein TIFY 6b-like [*Malus* *domestica*] | Stage 4 vs. Stage 5 |
| MD05G1256300 | NPR4a [*Malus* *domestica*] | Stage 4 vs. Stage 5 |
| MD01G1083400 | PREDICTED: auxin response factor 18-like isoform X1 [*Malus* *domestica*] | Stage 4 vs. Stage 5 |
| MD05G1052200 | PREDICTED: auxin-induced protein 15A-like [*Pyrus* x *bretschneideri*] | Stage 4 vs. Stage 5 |
| MD12G1113400 | PREDICTED: uncharacterized protein LOC103450013 [*Malus* *domestica*] | Stage 4 vs. Stage 5 |
| MD05G1113400 | hypothetical protein PRUPE_8G157900 [*Prunus persica*] | Stage 4 vs. Stage 5 |
| MD05G1223400 | PREDICTED: auxin-responsive protein SAUR71-like [*Malus* *domestica*] | Stage 4 vs. Stage 5 |
| MD07G1215900 | PREDICTED: auxin transporter-like protein 2 [*Malus* *domestica*] | Stage 4 vs. Stage 5 |
| MD17G1189100 | PREDICTED: LOW QUALITY PROTEIN: auxin-responsive protein IAA21-like [*Malus* *domestica*] | Stage 4 vs. Stage 5 |
| MD13G1176300 | histidine-containing phosphotransfer 1a [*Malus* *domestica*] | Stage 4 vs. Stage 5 |
| MD10G1060600 | PREDICTED: auxin-induced protein 15A-like [*Malus* *domestica*] | Stage 4 vs. Stage 5 |
| MD10G1059200 | PREDICTED: auxin-induced protein 15A-like [*Malus* *domestica*] | Stage 4 vs. Stage 5 |
| MD13G1222200 | ARF domain class transcription factor [*Malus* *domestica*] | Stage 4 vs. Stage 5 |
| MD15G1180500 | DELLA protein GAI-like [*Malus* *domestica*] | Stage 4 vs. Stage 5 |
| MD15G1413000 | PREDICTED: BRASSINOSTEROID INSENSITIVE 1-associated receptor kinase 1-like [*Malus* *domestica*] | Stage 4 vs. Stage 5 |
| MD10G1192900 | PREDICTED: auxin-induced protein AUX28 [*Malus* *domestica*] | Stage 4 vs. Stage 5 |
| MD07G1172500 | PREDICTED: auxin-induced protein 15A-like [*Malus* *domestica*] | Stage 4 vs. Stage 5 |
| MD01G1021600 | PREDICTED: transcription factor TGA4-like isoform X4 [*Malus* *domestica*] | Stage 4 vs. Stage 5 |
| MD13G1127100 | PREDICTED: protein TIFY 9 [*Malus* *domestica*] | Stage 4 vs. Stage 5 |
| MD15G1060800 | PREDICTED: abscisic acid receptor PYL2-like [*Malus* *domestica*] | Stage 4 vs. Stage 5 |
| MD15G1305800 | PREDICTED: auxin-responsive protein IAA9 [*Pyrus* x *bretschneideri*] | Stage 4 vs. Stage 5 |
| MD12G1061900 | PREDICTED: transcription factor HBP-1b(c38)-like [*Malus* *domestica*] | Stage 4 vs. Stage 5 |
| MD09G1121900 | CHASE histidine kinase 2 [*Malus* *domestica*] | Stage 4 vs. Stage 5 |
| MD04G1212100 | PREDICTED: histidine-containing phosphotransfer protein 1 [*Malus* *domestica*] | Stage 4 vs. Stage 5 |
| MD06G1046300 | serine/threonine-protein kinase SAPK3-like [*Malus* *domestica*] | Stage 4 vs. Stage 5 |
| MD00G1165200 | PREDICTED: jasmonic acid-amido synthetase JAR1 isoform X1 [*Malus* *domestica*] | Stage 4 vs. Stage 5 |
| MD11G1085000 | PREDICTED: probable serine/threonine-protein kinase At5g41260 [*Malus* *domestica*] | Stage 4 vs. Stage 5 |
| MD16G1212500 | PREDICTED: ethylene receptor 2-like [*Malus* *domestica*] | Stage 4 vs. Stage 5 |
| MD05G1051800 | PREDICTED: auxin-induced protein 15A-like [*Malus* *domestica*] | Stage 4 vs. Stage 5 |
| MD09G1208000 | PREDICTED: auxin-responsive protein IAA21-like isoform X2 [*Malus* *domestica*] | Stage 4 vs. Stage 5 |
| MD05G1205800 | PREDICTED: auxin-induced protein AUX28-like [*Malus* *domestica*] | Stage 4 vs. Stage 5 |
| MD04G1185100 | PREDICTED: transcription factor PIF3 isoform X1 [*Malus* *domestica*] | Stage 4 vs. Stage 5 |
| MD02G1178200 | PREDICTED: cyclin-D3-2 [*Malus* *domestica*] | Stage 4 vs. Stage 5 |
| MD15G1288100 | PREDICTED: cyclin-D3-2-like [*Malus* *domestica*] | Stage 4 vs. Stage 5 |
| MD17G1198100 | PREDICTED: auxin-induced protein IAA6 [*Malus* *domestica*] | Stage 4 vs. Stage 5 |
| MD09G1091000 | PREDICTED: jasmonic acid-amido synthetase JAR1 isoform X1 [*Malus* *domestica*] | Stage 4 vs. Stage 5 |
| MD16G1014900 | histidine kinase 4-like [*Malus* *domestica*] | Stage 4 vs. Stage 5 |
| MD03G1276700 | PREDICTED: protein TRANSPORT INHIBITOR RESPONSE 1-like [*Malus* *domestica*] | Stage 4 vs. Stage 5 |
| MD16G1142800 | PREDICTED: indole-3-acetic acid-amido synthetase GH3.17-like [*Malus* *domestica*] | Stage 4 vs. Stage 5 |
| MD13G1204700 | PREDICTED: auxin-induced protein 22D [*Malus* *domestica*] | Stage 4 vs. Stage 5 |
| *Malus*_*domestica*_newGene_5487 | PREDICTED: probable serine/threonine-protein kinase At4g35230 [*Pyrus* x *bretschneideri*] | Stage 4 vs. Stage 5 |
| MD04G1225000 | auxin-responsive protein IAA3-like [*Malus* *domestica*] | Stage 4 vs. Stage 5 |
| MD01G1220800 | PREDICTED: probable protein phosphatase 2C 51 isoform X1 [*Malus* *domestica*] | Stage 4 vs. Stage 5 |
| MD08G1014900 | PREDICTED: auxin-induced protein 15A-like [*Malus* *domestica*] | Stage 4 vs. Stage 5 |
| MD16G1127400 | PREDICTED: protein TIFY 9-like [*Pyrus* x *bretschneideri*] | Stage 4 vs. Stage 5 |
| MD14G1083700 | PREDICTED: regulatory protein NPR5-like [*Pyrus* x *bretschneideri*] | Stage 4 vs. Stage 5 |
| MD15G1414000 | PREDICTED: BRASSINOSTEROID INSENSITIVE 1-associated receptor kinase 1-like [*Malus* *domestica*] | Stage 4 vs. Stage 5 |
| MD15G1084100 | PREDICTED: BRI1 kinase inhibitor 1-like [*Malus* *domestica*] | Stage 4 vs. Stage 5 |
| MD05G1052000 | PREDICTED: auxin-induced protein 15A-like [*Malus* *domestica*] | Stage 4 vs. Stage 5 |
| MD10G1193000 | uncharacterized protein LOC103445716 [*Malus* *domestica*] | Stage 4 vs. Stage 5 |
| MD05G1092900 | PREDICTED: probable indole-3-acetic acid-amido synthetase GH3.1 [*Malus* *domestica*] | Stage 4 vs. Stage 5 |
| MD15G1014300 | PREDICTED: auxin-responsive protein SAUR71-like [*Malus* *domestica*] | Stage 4 vs. Stage 5 |
| MD00G1036600 | PREDICTED: auxin-responsive protein SAUR32-like [*Malus* *domestica*] | Stage 4 vs. Stage 5 |
| MD01G1149100 | PREDICTED: auxin transporter-like protein 1 [*Malus* *domestica*] | Stage 4 vs. Stage 5 |
| MD15G1373200 | PREDICTED: serine/threonine-protein kinase SAPK1-like isoform X2 [*Malus* *domestica*] | Stage 4 vs. Stage 5 |
| MD16G1108400 | PREDICTED: two-component response regulator ARR11-like isoform X1 [*Malus* *domestica*] | Stage 4 vs. Stage 5 |
| MD16G1178100 | PREDICTED: histidine-containing phosphotransfer protein 1 [*Malus* *domestica*] | Stage 4 vs. Stage 5 |
| MD17G1260700 | DELLA protein GAI-like [*Malus* *domestica*] | Stage 4 vs. Stage 5 |
| MD07G1297400 | PREDICTED: auxin-responsive protein SAUR32 [*Malus* *domestica*] | Stage 4 vs. Stage 5 |
| MD02G1100000 | PREDICTED: uncharacterized protein LOC103453925 isoform X2 [*Malus* *domestica*] | Stage 4 vs. Stage 5 |
| MD04G1149300 | PREDICTED: auxin transporter-like protein 2 isoform X1 [*Pyrus* x *bretschneideri*] | Stage 4 vs. Stage 5 |
| MD17G1183500 | PREDICTED: auxin-responsive protein IAA26-like [*Pyrus* x *bretschneideri*] | Stage 4 vs. Stage 5 |
| MD02G1105900 | PREDICTED: protein TIFY 3-like [*Malus* *domestica*] | Stage 4 vs. Stage 5 |
| MD05G1051700 | PREDICTED: auxin-induced protein 15A-like [*Malus* *domestica*] | Stage 4 vs. Stage 5 |
| MD08G1187200 | serine/threonine-protein kinase SAPK2 [*Malus* *domestica*] | Stage 4 vs. Stage 5 |
| MD15G1014400 | PREDICTED: auxin response factor 5 [*Malus* *domestica*] | Stage 4 vs. Stage 5 |
| MD14G1186400 | PREDICTED: probable serine/threonine-protein kinase At5g41260 isoform X1 [*Pyrus* x *bretschneideri*] | Stage 4 vs. Stage 5 |
| MD16G1020800 | PREDICTED: protein TIFY 6B-like [*Malus* *domestica*] | Stage 4 vs. Stage 5 |
| MD12G1226800 | PREDICTED: histidine-containing phosphotransfer protein 1 [*Malus* *domestica*] | Stage 4 vs. Stage 5 |
| MD05G1325800 | PREDICTED: protein TRANSPORT INHIBITOR RESPONSE 1-like [*Malus* *domestica*] | Stage 4 vs. Stage 5 |

| **Table S2**. List of genes from the MElightcyan module. | |
| --- | --- |
| **Gene ID** | **NR_annotation** |
| MD00G1004900 | PREDICTED: transcription factor TCP19-like [*Malus* *domestica*] |
| MD00G1005300 | PREDICTED: uncharacterized protein LOC103439700 [*Malus* *domestica*] |
| MD00G1021800 | PREDICTED: topless-related protein 1-like [*Malus* *domestica*] |
| MD00G1031300 | PREDICTED: pyruvate kinase isozyme A, chloroplastic-like isoform X2 [*Malus* *domestica*] |
| MD00G1033000 | PREDICTED: 4-coumarate--CoA ligase-like 7 [*Malus* *domestica*] |
| MD00G1036000 | -- |
| MD00G1039300 | PREDICTED: probable E3 ubiquitin-protein ligase ARI2 [*Malus* *domestica*] |
| MD00G1039900 | PREDICTED: B-box zinc finger protein 19-like isoform X1 [*Pyrus* x *bretschneideri*] |
| MD00G1040600 | PREDICTED: protein FMP32, mitochondrial-like isoform X1 [*Malus* *domestica*] |
| MD00G1046300 | PREDICTED: protein ROOT HAIR DEFECTIVE 3 homolog 2-like isoform X8 [*Pyrus* x *bretschneideri*] |
| MD00G1047200 | PREDICTED: protein ROOT HAIR DEFECTIVE 3 homolog 2-like isoform X8 [*Pyrus* x *bretschneideri*] |
| MD00G1054500 | PREDICTED: 2-hydroxyacyl-CoA lyase [*Malus* *domestica*] |
| MD00G1057700 | PREDICTED: uncharacterized protein LOC103434407 [*Malus* *domestica*] |
| MD00G1060400 | PREDICTED: cysteine-rich repeat secretory protein 56-like [*Malus* *domestica*] |
| MD00G1068800 | PREDICTED: transcription factor bHLH18-like [*Pyrus* x *bretschneideri*] |
| MD00G1077800 | PREDICTED: receptor-like protein 2 [*Malus* *domestica*] |
| MD00G1079900 | putative ribose-5-phosphate isomerase 3 chloroplastic [*Zea mays*] |
| MD00G1080300 | -- |
| MD00G1080400 | PREDICTED: uncharacterized protein LOC108172753 [*Malus* *domestica*] |
| MD00G1082000 | PREDICTED: organ-specific protein S2-like [*Malus* *domestica*] |
| MD00G1086900 | PREDICTED: uncharacterized protein LOC103446497 [*Malus* *domestica*] |
| MD00G1090600 | PREDICTED: uncharacterized protein LOC103932561 [*Pyrus* x *bretschneideri*] |
| MD00G1096000 | PREDICTED: E3 ubiquitin-protein ligase RMA1H1-like [*Malus* *domestica*] |
| MD00G1096700 | PREDICTED: uncharacterized protein LOC103424417 [*Malus* *domestica*] |
| MD00G1097000 | E3 ubiquitin-protein ligase At3g02290 [*Prunus persica*] |
| MD00G1104600 | PREDICTED: probable serine/threonine protein kinase IRE [*Pyrus* x *bretschneideri*] |
| MD00G1106600 | PREDICTED: protein YLS9-like [*Malus* *domestica*] |
| MD00G1106900 | PREDICTED: uncharacterized protein LOC103400446 [*Malus* *domestica*] |
| MD00G1107600 | PREDICTED: probable protein S-acyltransferase 4 [*Pyrus* x *bretschneideri*] |
| MD00G1109200 | PREDICTED: uncharacterized protein LOC103938571 [*Pyrus* x *bretschneideri*] |
| MD00G1114800 | PREDICTED: autophagy-related protein 8f [*Malus* *domestica*] |
| MD00G1121500 | PREDICTED: 7-deoxyloganetin glucosyltransferase-like [*Malus* *domestica*] |
| MD00G1123300 | PREDICTED: haloacid dehalogenase-like hydrolase domain-containing protein Sgpp [*Malus* *domestica*] |
| MD00G1127700 | PREDICTED: late embryogenesis abundant protein Lea5-A [*Malus* *domestica*] |
| MD00G1129100 | PREDICTED: serine/threonine-protein kinase Nek5-like [*Pyrus* x *bretschneideri*] |
| MD00G1131300 | PREDICTED: uncharacterized protein At5g19025 [*Malus* *domestica*] |
| MD00G1132300 | PREDICTED: decapping nuclease DXO homolog, chloroplastic-like [*Malus* *domestica*] |
| MD00G1132400 | PREDICTED: protein ABA DEFICIENT 4, chloroplastic-like isoform X2 [*Malus* *domestica*] |
| MD00G1135400 | PREDICTED: uncharacterized protein LOC103952911 [*Pyrus* x *bretschneideri*] |
| MD00G1145100 | PREDICTED: LOW QUALITY PROTEIN: methylenetetrahydrofolate reductase 2-like, partial [*Malus* *domestica*] |
| MD00G1147000 | PREDICTED: uncharacterized protein At1g76660-like [*Pyrus* x *bretschneideri*] |
| MD00G1150500 | PREDICTED: LOW QUALITY PROTEIN: 10 kDa chaperonin-like [*Malus* *domestica*] |
| MD00G1162000 | PREDICTED: 1-acylglycerol-3-phosphate O-acyltransferase-like [*Malus* *domestica*] |
| MD00G1165100 | PREDICTED: protein DOWNY MILDEW RESISTANCE 6-like isoform X1 [*Malus* *domestica*] |
| MD00G1165500 | PREDICTED: dolichol kinase EVAN isoform X3 [*Malus* *domestica*] |
| MD00G1171800 | PREDICTED: probable alpha,alpha-trehalose-phosphate synthase [UDP-forming] 10 [*Malus* *domestica*] |
| MD00G1181400 | PREDICTED: homocysteine S-methyltransferase 3 [*Pyrus* x *bretschneideri*] |
| MD00G1185500 | PREDICTED: C2 domain-containing protein At1g53590-like isoform X2 [*Malus* *domestica*] |
| MD00G1189000 | PREDICTED: heavy metal-associated isoprenylated plant protein 3-like [*Pyrus* x *bretschneideri*] |
| MD00G1190800 | PREDICTED: peroxidase 12-like [*Malus* *domestica*] |
| MD00G1193100 | PREDICTED: protein NETWORKED 4A-like isoform X1 [*Malus* *domestica*] |
| MD00G1200100 | PREDICTED: glycolipid transfer protein 1-like [*Malus* *domestica*] |
| MD00G1208200 | PREDICTED: uncharacterized protein LOC103434072 [*Malus* *domestica*] |
| MD00G1215600 | PREDICTED: peroxisomal multifunctional enzyme A-like [*Pyrus* x *bretschneideri*] |
| MD00G1220300 | PREDICTED: 1-aminocyclopropane-1-carboxylate oxidase homolog 3-like [*Malus* *domestica*] |
| MD00G1220800 | PREDICTED: oxysterol-binding protein-related protein 1C-like isoform X2 [*Malus* *domestica*] |
| MD01G1006200 | PREDICTED: uncharacterized protein LOC103432869 isoform X5 [*Malus* *domestica*] |
| MD01G1009700 | PREDICTED: ribonuclease P protein subunit p25-like protein [*Pyrus* x *bretschneideri*] |
| MD01G1024400 | PREDICTED: bifunctional aspartate aminotransferase and glutamate/aspartate-prephenate aminotransferase-like [*Malus* *domestica*] |
| MD01G1025400 | PREDICTED: probable glucan 1,3-beta-glucosidase A [*Pyrus* x *bretschneideri*] |
| MD01G1027400 | PREDICTED: PLAT domain-containing protein 3-like [*Pyrus* x *bretschneideri*] |
| MD01G1029100 | PREDICTED: annexin D2-like [*Pyrus* x *bretschneideri*] |
| MD01G1032700 | PREDICTED: classical arabinogalactan protein 4-like [*Malus* *domestica*] |
| MD01G1040300 | -- |
| MD01G1041300 | PREDICTED: probable 1-deoxy-D-xylulose-5-phosphate synthase 2, chloroplastic [*Malus* *domestica*] |
| MD01G1046600 | PREDICTED: probable sphingolipid transporter spinster homolog 2 isoform X3 [*Malus* *domestica*] |
| MD01G1046700 | PREDICTED: 2-alkenal reductase (NADP(+)-dependent)-like [*Prunus mume*] |
| MD01G1049600 | PREDICTED: BON1-associated protein 2-like [*Malus* *domestica*] |
| MD01G1051300 | PREDICTED: uncharacterized protein LOC103331974 [*Prunus mume*] |
| MD01G1051900 | N-acetylserotonin O-methyltransferase [*Malus* zumi] |
| MD01G1054400 | uncharacterized protein LOC18786106 [*Prunus persica*] |
| MD01G1054800 | myb-related protein Myb4-like [*Malus* *domestica*] |
| MD01G1059700 | PREDICTED: DISEASE-RESISTANCE LOCUS RECEPTOR-LIKE PROTEIN KINASE-like 2.1 [*Pyrus* x *bretschneideri*] |
| MD01G1061100 | PREDICTED: DEAD-box ATP-dependent RNA helicase 28-like [*Pyrus* x *bretschneideri*] |
| MD01G1065600 | L-3-cyanoalanine synthase 2, mitochondrial [*Malus* *domestica*] |
| MD01G1065900 | PREDICTED: ubiquitin-conjugating enzyme E2-23 kDa-like isoform X1 [*Malus* *domestica*] |
| MD01G1066500 | PREDICTED: probable E3 ubiquitin-protein ligase RHB1A [*Pyrus* x *bretschneideri*] |
| MD01G1068500 | PREDICTED: soyasaponin III rhamnosyltransferase-like [*Malus* *domestica*] |
| MD01G1069600 | PREDICTED: sphingoid long-chain bases kinase 2, mitochondrial-like isoform X2 [*Malus* *domestica*] |
| MD01G1071900 | PREDICTED: desiccation protectant protein Lea14 homolog [*Malus* *domestica*] |
| MD01G1087300 | PREDICTED: CBL-interacting serine/threonine-protein kinase 9-like [*Malus* *domestica*] |
| MD01G1087400 | PREDICTED: 3-ketoacyl-CoA synthase 1-like [*Malus* *domestica*] |
| MD01G1088200 | PREDICTED: vacuolar-processing enzyme-like [*Malus* *domestica*] |
| MD01G1089800 | PREDICTED: caffeic acid 3-O-methyltransferase-like [*Malus* *domestica*] |
| MD01G1093200 | PREDICTED: NAC domain-containing protein 62-like [*Malus* *domestica*] |
| MD01G1094700 | PREDICTED: NAC domain-containing protein 5-like isoform X1 [*Malus* *domestica*] |
| MD01G1103300 | PREDICTED: glycerol-3-phosphate dehydrogenase SDP6, mitochondrial isoform X1 [*Malus* *domestica*] |
| MD01G1103800 | hypothetical protein PRUPE_2G209600 [*Prunus persica*] |
| MD01G1107300 | PREDICTED: uncharacterized protein LOC103406265 [*Malus* *domestica*] |
| MD01G1110900 | PREDICTED: uncharacterized protein LOC103432448 [*Malus* *domestica*] |
| MD01G1118000 | chalcone isomerase [*Pyrus* *pyrifolia*] |
| MD01G1118100 | PREDICTED: chalcone--flavonone isomerase-like isoform X3 [*Malus* *domestica*] |
| MD01G1119700 | PREDICTED: caffeoylshikimate esterase-like [*Malus* *domestica*] |
| MD01G1120400 | PREDICTED: uncharacterized protein LOC103426379 [*Malus* *domestica*] |
| MD01G1133400 | PREDICTED: heat shock cognate 70 kDa protein-like [*Pyrus* x *bretschneideri*] |
| MD01G1139800 | PREDICTED: wall-associated receptor kinase-like 20 [*Pyrus* x *bretschneideri*] |
| MD01G1143100 | PREDICTED: UDP-glycosyltransferase 76F1-like [*Pyrus* x *bretschneideri*] |
| MD01G1146200 | PREDICTED: uncharacterized protein At2g39920-like [*Malus* *domestica*] |
| MD01G1147600 | PREDICTED: peroxisomal nicotinamide adenine dinucleotide carrier-like isoform X2 [*Malus* *domestica*] |
| MD01G1157700 | PREDICTED: ferritin-3, chloroplastic-like [*Malus* *domestica*] |
| MD01G1159900 | PREDICTED: biotin carboxyl carrier protein of acetyl-CoA carboxylase-like [*Malus* *domestica*] |
| MD01G1160300 | PREDICTED: protein EARLY RESPONSIVE TO DEHYDRATION 15-like [*Malus* *domestica*] |
| MD01G1162700 | PREDICTED: peroxidase P7-like [*Malus* *domestica*] |
| MD01G1163900 | PREDICTED: adenine/guanine permease AZG1-like [*Pyrus* x *bretschneideri*] |
| MD01G1164900 | tRNA (guanine(26)-N(2))-dimethyltransferase isoform X4 [*Prunus avium*] |
| MD01G1169200 | PREDICTED: uncharacterized protein LOC108169406 [*Malus* *domestica*] |
| MD01G1172000 | PREDICTED: protein SRC2 homolog [*Malus* *domestica*] |
| MD01G1173500 | PREDICTED: metacaspase-1-like [*Malus* *domestica*] |
| MD01G1177000 | ethylene-responsive transcription factor 2-like [*Malus* *domestica*] |
| MD01G1178900 | PREDICTED: receptor-like protein 12 [*Malus* *domestica*] |
| MD01G1180200 | PREDICTED: uncharacterized protein LOC103942294 [*Pyrus* x *bretschneideri*] |
| MD01G1180900 | PREDICTED: thiol protease aleurain-like, partial [*Malus* *domestica*] |
| MD01G1188000 | PREDICTED: cytochrome P450 704C1-like [*Pyrus* x *bretschneideri*] |
| MD01G1188200 | PREDICTED: cytochrome P450 704C1-like [*Malus* *domestica*] |
| MD01G1194400 | PREDICTED: uncharacterized protein LOC103439700 [*Malus* *domestica*] |
| MD01G1195100 | PREDICTED: sorbitol dehydrogenase-like [*Malus* *domestica*] |
| MD01G1195200 | sorbitol dehydrogenase-like [*Malus* *domestica*] |
| MD01G1195700 | PREDICTED: calcium-dependent protein kinase 26-like [*Malus* *domestica*] |
| MD01G1196100 | PREDICTED: dehydration-responsive element-binding protein 1B [*Malus* *domestica*] |
| MD01G1199300 | PREDICTED: uncharacterized protein LOC103441353 [*Malus* *domestica*] |
| MD01G1204300 | PREDICTED: cytochrome P450 89A2-like [*Malus* *domestica*] |
| MD01G1204400 | PREDICTED: cytochrome P450 89A2-like [*Malus* *domestica*] |
| MD01G1206700 | PREDICTED: protein DETOXIFICATION 35-like [*Malus* *domestica*] |
| MD01G1209900 | PREDICTED: pentatricopeptide repeat-containing protein At4g21190-like [*Malus* *domestica*] |
| MD01G1211100 | PREDICTED: reticulon-like protein B1 [*Malus* *domestica*] |
| MD01G1213300 | PREDICTED: acidic endochitinase SE2-like [*Malus* *domestica*] |
| MD01G1214100 | PREDICTED: probable inactive receptor kinase At4g23740 [*Malus* *domestica*] |
| MD01G1217700 | PREDICTED: uncharacterized protein At4g00950-like [*Pyrus* x *bretschneideri*] |
| MD01G1218900 | PREDICTED: caffeic acid 3-O-methyltransferase-like isoform X1 [*Malus* *domestica*] |
| MD01G1220400 | PREDICTED: zinc finger protein CONSTANS-LIKE 2-like isoform X1 [*Malus* *domestica*] |
| MD01G1226600 | homeobox-leucine zipper protein ATHB-7-like [*Malus* *domestica*] |
| MD01G1226700 | -- |
| MD01G1227100 | PREDICTED: auxin-responsive protein SAUR32-like [*Malus* *domestica*] |
| MD01G1227700 | PREDICTED: LOW QUALITY PROTEIN: polyadenylate-binding protein-interacting protein 12 [*Malus* *domestica*] |
| MD01G1229100 | caffeic acid O-methyltransferase [*Malus* *domestica*] |
| MD01G1229700 | PREDICTED: heavy metal-associated isoprenylated plant protein 3-like [*Malus* *domestica*] |
| MD01G1232800 | PREDICTED: lecithin retinol acyltransferase-like [*Malus* *domestica*] |
| MD01G1236500 | PREDICTED: cellulose synthase-like protein D3 [*Malus* *domestica*] |
| MD01G1236600 | -- |
| MD01G1239000 | PREDICTED: GPCR-type G protein 1-like isoform X3 [*Pyrus* x *bretschneideri*] |
| MD02G1001400 | unnamed protein product, partial [*Vitis vinifera*] |
| MD02G1001900 | PREDICTED: 2-alkenal reductase (NADP(+)-dependent)-like [*Malus* *domestica*] |
| MD02G1010800 | PREDICTED: calmodulin-binding protein 60 E-like [*Pyrus* x *bretschneideri*] |
| MD02G1011100 | class V chitinase [*Pyrus* x *bretschneideri* x *Pyrus* *pyrifolia*] |
| MD02G1013900 | PREDICTED: methanol O-anthraniloyltransferase-like [*Malus* *domestica*] |
| MD02G1014800 | alcohol acyl transferase [*Malus* *domestica*] |
| MD02G1015200 | alcohol acyl transferase [*Malus* *domestica*] |
| MD02G1023800 | PREDICTED: F-box/kelch-repeat protein At3g06240-like isoform X1 [*Pyrus* x *bretschneideri*] |
| MD02G1032300 | -- |
| MD02G1038100 | PREDICTED: EG45-like domain containing protein [*Malus* *domestica*] |
| MD02G1039600 | PREDICTED: LOW QUALITY PROTEIN: DELLA protein GAI [*Malus* *domestica*] |
| MD02G1045800 | PREDICTED: proline-rich receptor-like protein kinase PERK1 [*Malus* *domestica*] |
| MD02G1047700 | hypothetical protein EUGRSUZ_C01044 [Eucalyptus grandis] |
| MD02G1048100 | hypothetical protein PRUPE_7G230000 [*Prunus persica*] |
| MD02G1048400 | PREDICTED: uncharacterized protein LOC103449036 [*Malus* *domestica*] |
| MD02G1048700 | hypothetical protein PRUPE_7G229500 [*Prunus persica*] |
| MD02G1050800 | PREDICTED: 1-aminocyclopropane-1-carboxylate oxidase homolog 3-like [*Malus* *domestica*] |
| MD02G1053100 | PREDICTED: TMV resistance protein N-like [*Pyrus* x *bretschneideri*] |
| MD02G1053300 | PREDICTED: 1-aminocyclopropane-1-carboxylate oxidase homolog 3-like [*Malus* *domestica*] |
| MD02G1056200 | PREDICTED: VIN3-like protein 1 isoform X1 [*Malus* *domestica*] |
| MD02G1056400 | PREDICTED: strigolactone esterase D14-like [*Malus* *domestica*] |
| MD02G1060200 | PREDICTED: ethylene-responsive transcription factor TINY-like [*Malus* *domestica*] |
| MD02G1062300 | PREDICTED: uncharacterized protein LOC103449249 [*Malus* *domestica*] |
| MD02G1065600 | PREDICTED: uncharacterized protein LOC103449718 [*Malus* *domestica*] |
| MD02G1067200 | PREDICTED: probable glutamate carboxypeptidase 2, partial [*Malus* *domestica*] |
| MD02G1067800 | PREDICTED: glucan endo-1,3-beta-glucosidase 13 [*Pyrus* x *bretschneideri*] |
| MD02G1074400 | PREDICTED: uncharacterized protein LOC103426665 isoform X1 [*Malus* *domestica*] |
| MD02G1079200 | beta-galactosidase precursor [*Malus* *domestica*] |
| MD02G1084200 | PREDICTED: uncharacterized protein LOC103426741 [*Malus* *domestica*] |
| MD02G1084600 | PREDICTED: protein phosphatase 2C 77-like [*Malus* *domestica*] |
| MD02G1094000 | PREDICTED: uncharacterized protein LOC103929707 isoform X1 [*Pyrus* x *bretschneideri*] |
| MD02G1096500 | ethylene-responsive transcription factor ERF017-like [*Malus* *domestica*] |
| MD02G1097500 | PREDICTED: 3-oxoacyl-[acyl-carrier-protein] synthase II, chloroplastic-like [*Pyrus* x *bretschneideri*] |
| MD02G1097900 | PREDICTED: serine/threonine-protein kinase pakG-like [*Malus* *domestica*] |
| MD02G1099300 | PREDICTED: uncharacterized protein LOC103416009 [*Malus* *domestica*] |
| MD02G1100200 | PREDICTED: auxin-responsive protein SAUR72-like [*Pyrus* x *bretschneideri*] |
| MD02G1105300 | PREDICTED: alpha-aminoadipic semialdehyde synthase-like [*Malus* *domestica*] |
| MD02G1106200 | PREDICTED: N-acylphosphatidylethanolamine synthase-like isoform X1 [*Malus* *domestica*] |
| MD02G1106900 | -- |
| MD02G1107700 | PREDICTED: diacylglycerol O-acyltransferase 2-like [*Pyrus* x *bretschneideri*] |
| MD02G1109900 | -- |
| MD02G1112400 | PREDICTED: TMV resistance protein N-like [*Pyrus* x *bretschneideri*] |
| MD02G1113600 | PREDICTED: uncharacterized protein LOC103959201, partial [*Pyrus* x *bretschneideri*] |
| MD02G1113800 | PREDICTED: protein PIN-LIKES 5-like [*Malus* *domestica*] |
| MD02G1114200 | PREDICTED: probable protein phosphatase 2C 47 [*Malus* *domestica*] |
| MD02G1122200 | PREDICTED: ganglioside-induced differentiation-associated protein 2 [*Malus* *domestica*] |
| MD02G1124500 | PREDICTED: aspartyl protease family protein 1 isoform X1 [*Malus* *domestica*] |
| MD02G1127500 | PREDICTED: oleoyl-acyl carrier protein thioesterase 1, chloroplastic-like isoform X2 [*Malus* *domestica*] |
| MD02G1128700 | PREDICTED: alpha-mannosidase-like [*Malus* *domestica*] |
| MD02G1133600 | PREDICTED: LOW QUALITY PROTEIN: palmitoyl-monogalactosyldiacylglycerol delta-7 desaturase, chloroplastic-like [*Malus* *domestica*] |
| MD02G1136000 | PREDICTED: cytochrome P450 84A1-like [*Malus* *domestica*] |
| MD02G1139900 | PREDICTED: dehydrin Xero 1-like [*Malus* *domestica*] |
| MD02G1148100 | PREDICTED: CRAL-TRIO domain-containing protein C23B6.04c-like [*Pyrus* x *bretschneideri*] |
| MD02G1148700 | PREDICTED: uncharacterized protein LOC103426803 [*Malus* *domestica*] |
| MD02G1150100 | PREDICTED: F-box protein At5g07610-like [*Malus* *domestica*] |
| MD02G1153100 | PREDICTED: anthocyanidin 3-O-glucosyltransferase 5-like [*Malus* *domestica*] |
| MD02G1153700 | PREDICTED: anthocyanidin 3-O-glucosyltransferase 5-like [*Malus* *domestica*] |
| MD02G1161400 | PREDICTED: salicylic acid-binding protein 2-like [*Malus* *domestica*] |
| MD02G1166700 | PREDICTED: uncharacterized protein LOC103418344 isoform X1 [*Malus* *domestica*] |
| MD02G1168400 | PREDICTED: zinc finger CCCH domain-containing protein 62-like isoform X2 [*Pyrus* x *bretschneideri*] |
| MD02G1174700 | PREDICTED: F-box protein At5g67140 [*Malus* *domestica*] |
| MD02G1175500 | PREDICTED: uncharacterized acetyltransferase At3g50280-like [*Malus* *domestica*] |
| MD02G1187000 | PREDICTED: NADH dehydrogenase [ubiquinone] iron-sulfur protein 4, mitochondrial [*Malus* *domestica*] |
| MD02G1187800 | PREDICTED: heptahelical transmembrane protein 4-like isoform X2 [*Pyrus* x *bretschneideri*] |
| MD02G1190500 | PREDICTED: probable pectinesterase 29 [*Malus* *domestica*] |
| MD02G1194000 | PREDICTED: uncharacterized protein LOC103405414 [*Malus* *domestica*] |
| MD02G1194900 | PREDICTED: UDP-galactose/UDP-glucose transporter 2 isoform X2 [*Malus* *domestica*] |
| MD02G1201500 | PREDICTED: uncharacterized protein LOC103405871 [*Malus* *domestica*] |
| MD02G1209900 | PREDICTED: spindle and kinetochore-associated protein 1 homolog [*Pyrus* x *bretschneideri*] |
| MD02G1210100 | autophagy-related protein 8f [*Malus* *domestica*] |
| MD02G1222400 | PREDICTED: probable receptor-like protein kinase At5g47070 [*Malus* *domestica*] |
| MD02G1222600 | PREDICTED: uncharacterized protein LOC103960230 [*Pyrus* x *bretschneideri*] |
| MD02G1226200 | PREDICTED: haloacid dehalogenase-like hydrolase domain-containing protein Sgpp [*Pyrus* x *bretschneideri*] |
| MD02G1226400 | PREDICTED: haloacid dehalogenase-like hydrolase domain-containing protein Sgpp [*Malus* *domestica*] |
| MD02G1229900 | PREDICTED: 3-hydroxyisobutyryl-CoA hydrolase 1-like isoform X3 [*Malus* *domestica*] |
| MD02G1234900 | PREDICTED: LEAF RUST 10 DISEASE-RESISTANCE LOCUS RECEPTOR-LIKE PROTEIN KINASE-like 2.5 [*Pyrus* x *bretschneideri*] |
| MD02G1243200 | PREDICTED: NAC domain-containing protein 78-like [*Malus* *domestica*] |
| MD02G1250900 | PREDICTED: putative disease resistance protein At3g14460 [*Malus* *domestica*] |
| MD02G1251900 | PREDICTED: putative disease resistance protein At3g14460 [*Malus* *domestica*] |
| MD02G1253600 | PREDICTED: rust resistance kinase Lr10-like, partial [*Malus* *domestica*] |
| MD02G1255400 | PREDICTED: copper transport protein ATX1 [*Malus* *domestica*] |
| MD02G1264100 | PREDICTED: NADP-dependent D-sorbitol-6-phosphate dehydrogenase-like [*Malus* *domestica*] |
| MD02G1265300 | PREDICTED: ethylene-responsive transcription factor WRI1 [*Pyrus* x *bretschneideri*] |
| MD02G1266900 | PREDICTED: protein ZINC INDUCED FACILITATOR-LIKE 1-like [*Malus* *domestica*] |
| MD02G1267000 | PREDICTED: protein ZINC INDUCED FACILITATOR-LIKE 1-like isoform X1 [*Malus* *domestica*] |
| MD02G1267400 | PREDICTED: MATH domain and coiled-coil domain-containing protein At3g58370-like [*Malus* *domestica*] |
| MD02G1274500 | PREDICTED: rust resistance kinase Lr10-like [*Malus* *domestica*] |
| MD02G1275000 | PREDICTED: cysteine proteinase inhibitor 12-like isoform X1 [*Malus* *domestica*] |
| MD02G1287700 | protein NRT1/ PTR FAMILY 5.4-like, partial [*Prunus avium*] |
| MD02G1291200 | PREDICTED: annexin D4-like [*Pyrus* x *bretschneideri*] |
| MD02G1292200 | PREDICTED: repetitive proline-rich cell wall protein 2 [*Pyrus* x *bretschneideri*] |
| MD02G1298800 | -- |
| MD02G1299000 | PREDICTED: pheophytinase, chloroplastic-like [*Pyrus* x *bretschneideri*] |
| MD02G1301300 | PREDICTED: protein BPS1, chloroplastic-like [*Malus* *domestica*] |
| MD02G1303800 | PREDICTED: uncharacterized protein LOC103945503 [*Pyrus* x *bretschneideri*] |
| MD02G1304400 | PREDICTED: cinnamoyl-CoA reductase 1-like isoform X1 [*Malus* *domestica*] |
| MD02G1305800 | PREDICTED: uncharacterized protein LOC103412949 [*Malus* *domestica*] |
| MD02G1313500 | PREDICTED: uncharacterized protein LOC103413235 [*Malus* *domestica*] |
| MD02G1319100 | PREDICTED: callose synthase 1-like [*Malus* *domestica*] |
| MD02G1319200 | PREDICTED: uncharacterized protein LOC103413530 [*Malus* *domestica*] |
| MD03G1005700 | PREDICTED: transcription initiation factor TFIID subunit 7-like [*Malus* *domestica*] |
| MD03G1007100 | PREDICTED: uncharacterized protein LOC103415080 [*Malus* *domestica*] |
| MD03G1018200 | PREDICTED: uncharacterized protein LOC103407558 [*Malus* *domestica*] |
| MD03G1019600 | -- |
| MD03G1028700 | PREDICTED: transmembrane emp24 domain-containing protein p24beta2-like [*Malus* *domestica*] |
| MD03G1032300 | PREDICTED: 1-aminocyclopropane-1-carboxylate oxidase homolog 3-like [*Malus* *domestica*] |
| MD03G1039200 | PREDICTED: BTB/POZ domain-containing protein At1g55760 isoform X2 [*Malus* *domestica*] |
| MD03G1044000 | PREDICTED: FH protein interacting protein FIP2-like [*Malus* *domestica*] |
| MD03G1048900 | root phototropism protein 2-like [*Malus* *domestica*] |
| MD03G1050500 | PREDICTED: LOW QUALITY PROTEIN: protein NETWORKED 4A-like [*Malus* *domestica*] |
| MD03G1062900 | PREDICTED: probable phytol kinase 3, chloroplastic [*Prunus mume*] |
| MD03G1064100 | PREDICTED: probable N-acetyltransferase HLS1 [*Pyrus* x *bretschneideri*] |
| MD03G1066200 | PREDICTED: protein PMR5-like [*Malus* *domestica*] |
| MD03G1073800 | PREDICTED: LOW QUALITY PROTEIN: 50S ribosomal protein L4, chloroplastic-like [*Malus* *domestica*] |
| MD03G1080500 | PREDICTED: pentatricopeptide repeat-containing protein At5g67570, chloroplastic [*Pyrus* x *bretschneideri*] |
| MD03G1082800 | PREDICTED: universal stress protein PHOS32-like [*Malus* *domestica*] |
| MD03G1090700 | PREDICTED: expansin-A6 [*Malus* *domestica*] |
| MD03G1096000 | PREDICTED: uncharacterized protein At3g17950-like [*Malus* *domestica*] |
| MD03G1096900 | PREDICTED: uncharacterized protein LOC103421264 [*Malus* *domestica*] |
| MD03G1100400 | PREDICTED: uncharacterized protein LOC103427173 [*Malus* *domestica*] |
| MD03G1105200 | PREDICTED: exonuclease V, chloroplastic [*Malus* *domestica*] |
| MD03G1108500 | PREDICTED: mitogen-activated protein kinase 3-like [*Malus* *domestica*] |
| MD03G1111700 | PREDICTED: uncharacterized protein LOC103427185 [*Malus* *domestica*] |
| MD03G1116100 | PREDICTED: isovaleryl-CoA dehydrogenase, mitochondrial [*Pyrus* x *bretschneideri*] |
| MD03G1116800 | PREDICTED: LOW QUALITY PROTEIN: uncharacterized protein LOC103422843 [*Malus* *domestica*] |
| MD03G1117900 | PREDICTED: aquaporin PIP2-2 [*Malus* *domestica*] |
| MD03G1125200 | PREDICTED: uncharacterized protein LOC103407783 [*Malus* *domestica*] |
| MD03G1125900 | PREDICTED: UDP-glycosyltransferase 86A1-like [*Malus* *domestica*] |
| MD03G1128800 | zinc finger protein ZAT5-like [*Malus* *domestica*] |
| MD03G1142300 | PREDICTED: zinc finger A20 and AN1 domain-containing stress-associated protein 3-like [*Malus* *domestica*] |
| MD03G1148700 | PREDICTED: capsanthin/capsorubin synthase, chromoplastic-like [*Malus* *domestica*] |
| MD03G1149700 | PREDICTED: cold-inducible RNA-binding protein-like [*Malus* *domestica*] |
| MD03G1150600 | PREDICTED: uncharacterized protein LOC103424822 [*Malus* *domestica*] |
| MD03G1159500 | PREDICTED: uncharacterized protein LOC103448122 isoform X2 [*Malus* *domestica*] |
| MD03G1164800 | dihydropyrimidinase [*Prunus persica*] |
| MD03G1166500 | PREDICTED: uncharacterized protein LOC103441872 [*Malus* *domestica*] |
| MD03G1172000 | PREDICTED: uncharacterized protein LOC103418956 [*Malus* *domestica*] |
| MD03G1172700 | PREDICTED: protein RETICULATA-RELATED 4, chloroplastic [*Malus* *domestica*] |
| MD03G1172800 | PREDICTED: cinnamoyl-CoA reductase 1 [*Malus* *domestica*] |
| MD03G1179900 | -- |
| MD03G1183800 | PREDICTED: two-pore potassium channel 1-like [*Malus* *domestica*] |
| MD03G1185600 | G-type lectin S-receptor-like serine/threonine-protein kinase At4g27290 isoform X1 [*Prunus avium*] |
| MD03G1191800 | PREDICTED: uncharacterized protein LOC108173951 [*Malus* *domestica*] |
| MD03G1192500 | PREDICTED: NADH dehydrogenase [ubiquinone] 1 alpha subcomplex subunit 2 [*Prunus mume*] |
| MD03G1200400 | PREDICTED: protein translation factor SUI1 homolog [*Malus* *domestica*] |
| MD03G1208900 | PREDICTED: glucose-6-phosphate/phosphate translocator 1, chloroplastic-like [*Malus* *domestica*] |
| MD03G1212700 | PREDICTED: protein SUPPRESSOR OF GENE SILENCING 3-like [*Malus* *domestica*] |
| MD03G1215100 | PREDICTED: uncharacterized protein LOC103429487 [*Malus* *domestica*] |
| MD03G1218200 | PREDICTED: uncharacterized protein LOC103430028 [*Malus* *domestica*] |
| MD03G1218500 | PREDICTED: cytochrome P450 71A25-like [*Pyrus* x *bretschneideri*] |
| MD03G1218600 | -- |
| MD03G1225000 | PREDICTED: cytochrome P450 CYP72A219-like [*Malus* *domestica*] |
| MD03G1226300 | PREDICTED: dynein light chain, cytoplasmic-like [*Malus* *domestica*] |
| MD03G1227600 | PREDICTED: cytochrome c oxidase copper chaperone 2 [*Pyrus* x *bretschneideri*] |
| MD03G1230900 | PREDICTED: transcription factor EMB1444-like [*Pyrus* x *bretschneideri*] |
| MD03G1236200 | PREDICTED: reticulon-4-interacting protein 1, mitochondrial-like [*Malus* *domestica*] |
| MD03G1239400 | PREDICTED: uncharacterized protein ycf45-like [*Malus* *domestica*] |
| MD03G1244700 | PREDICTED: LOW QUALITY PROTEIN: gamma-glutamyl hydrolase 2-like [*Malus* *domestica*] |
| MD03G1244800 | PREDICTED: LOW QUALITY PROTEIN: gamma-glutamyl hydrolase 2-like [*Malus* *domestica*] |
| MD03G1246200 | PREDICTED: BES1/BZR1 homolog protein 4-like [*Pyrus* x *bretschneideri*] |
| MD03G1263700 | PREDICTED: fatty-acid-binding protein 1-like [*Malus* *domestica*] |
| MD03G1269000 | PREDICTED: mediator of RNA polymerase II transcription subunit 33B-like [*Malus* *domestica*] |
| MD03G1271900 | PREDICTED: methyl-CpG-binding domain-containing protein 4-like isoform X1 [*Malus* *domestica*] |
| MD03G1272800 | PREDICTED: autophagy-related protein 18g [*Malus* *domestica*] |
| MD03G1273400 | -- |
| MD03G1277500 | -- |
| MD03G1278100 | PREDICTED: glutaredoxin-C11-like [*Malus* *domestica*] |
| MD03G1278600 | PREDICTED: BTB/POZ domain-containing protein At1g03010-like isoform X1 [*Malus* *domestica*] |
| MD03G1282700 | phi class glutathione S-transferase [*Malus* *domestica*] |
| MD03G1282900 | PREDICTED: glutathione S-transferase-like [*Malus* *domestica*] |
| MD03G1286600 | PREDICTED: probable E3 ubiquitin-protein ligase BAH1-like 2 [*Malus* *domestica*] |
| MD03G1287000 | PREDICTED: probable E3 ubiquitin-protein ligase BAH1-like 1 [*Malus* *domestica*] |
| MD03G1289600 | PREDICTED: poly [ADP-ribose] polymerase 2-like [*Malus* *domestica*] |
| MD03G1296100 | PREDICTED: uncharacterized protein LOC103432632 [*Malus* *domestica*] |
| MD03G1297400 | PREDICTED: uncharacterized protein LOC103432643 [*Malus* *domestica*] |
| MD04G1002400 | -- |
| MD04G1004900 | PREDICTED: glutathione reductase, cytosolic [*Malus* *domestica*] |
| MD04G1008900 | PREDICTED: ubiquitin-conjugating enzyme E2 4-like isoform X2 [*Pyrus* x *bretschneideri*] |
| MD04G1013500 | PREDICTED: probable sucrose-phosphate synthase 2 [*Malus* *domestica*] |
| MD04G1016000 | PREDICTED: glucose-6-phosphate 1-dehydrogenase 2, chloroplastic, partial [*Malus* *domestica*] |
| MD04G1017300 | PREDICTED: protein RALF-like 24 [*Pyrus* x *bretschneideri*] |
| MD04G1018500 | PREDICTED: selenium-binding protein 2-like [*Malus* *domestica*] |
| MD04G1018700 | PREDICTED: probable acyl-activating enzyme 16, chloroplastic isoform X1 [*Pyrus* x *bretschneideri*] |
| MD04G1019600 | PREDICTED: crocetin glucosyltransferase, chloroplastic-like [*Malus* *domestica*] |
| MD04G1021300 | PREDICTED: protein TIC 22-like, chloroplastic [*Malus* *domestica*] |
| MD04G1023200 | PREDICTED: cysteine proteinase inhibitor B [*Malus* *domestica*] |
| MD04G1024200 | PREDICTED: uncharacterized protein LOC103400623 [*Malus* *domestica*] |
| MD04G1030300 | PREDICTED: chitinase-like protein 1 [*Malus* *domestica*] |
| MD04G1031200 | PREDICTED: mitochondrial import inner membrane translocase subunit Tim16 [*Malus* *domestica*] |
| MD04G1032400 | PREDICTED: vesicle transport protein GOT1B [*Pyrus* x *bretschneideri*] |
| MD04G1033500 | PREDICTED: UDP-glycosyltransferase 92A1-like [*Malus* *domestica*] |
| MD04G1036500 | PREDICTED: EH domain-containing protein 1-like [*Pyrus* x *bretschneideri*] |
| MD04G1042400 | probable fructokinase-4 [*Prunus avium*] |
| MD04G1047700 | PREDICTED: endochitinase EP3-like [*Malus* *domestica*] |
| MD04G1051600 | PREDICTED: vesicle-associated protein 2-1 [*Malus* *domestica*] |
| MD04G1054200 | PREDICTED: coiled-coil domain-containing protein 97-like [*Malus* *domestica*] |
| MD04G1057600 | PREDICTED: oleosin 18.5 kDa-like [*Malus* *domestica*] |
| MD04G1060100 | probable glycosyltransferase At5g03795 [*Prunus avium*] |
| MD04G1063300 | PREDICTED: WAT1-related protein At5g47470 [*Malus* *domestica*] |
| MD04G1065500 | PREDICTED: RHOMBOID-like protein 2 [*Malus* *domestica*] |
| MD04G1070300 | PREDICTED: protein STAY-GREEN, chloroplastic-like [*Malus* *domestica*] |
| MD04G1082300 | PREDICTED: legumain [*Malus* *domestica*] |
| MD04G1083700 | PREDICTED: malignant T-cell-amplified sequence 1 homolog [*Pyrus* x *bretschneideri*] |
| MD04G1093300 | PREDICTED: heavy metal-associated isoprenylated plant protein 3-like isoform X1 [*Malus* *domestica*] |
| MD04G1111100 | PREDICTED: probable protein phosphatase 2C 38 [*Malus* *domestica*] |
| MD04G1112000 | PREDICTED: glutathione S-transferase L3-like isoform X2 [*Pyrus* x *bretschneideri*] |
| MD04G1132400 | PREDICTED: protein SRG1-like [*Pyrus* x *bretschneideri*] |
| MD04G1134300 | PREDICTED: uncharacterized protein LOC103939549 [*Pyrus* x *bretschneideri*] |
| MD04G1142100 | PREDICTED: uncharacterized protein LOC103433542 [*Malus* *domestica*] |
| MD04G1144500 | PREDICTED: uncharacterized protein At1g04910-like [*Malus* *domestica*] |
| MD04G1145100 | PREDICTED: mitochondrial uncoupling protein 1 [*Pyrus* x *bretschneideri*] |
| MD04G1146200 | PREDICTED: ataxin-3 homolog [*Malus* *domestica*] |
| MD04G1147700 | PREDICTED: laccase-4-like [*Malus* *domestica*] |
| MD04G1147800 | PREDICTED: transcription factor DIVARICATA [*Malus* *domestica*] |
| MD04G1154600 | PREDICTED: trihelix transcription factor GT-3b-like [*Malus* *domestica*] |
| MD04G1158500 | putative ribose-5-phosphate isomerase 3 chloroplastic [*Zea mays*] |
| MD04G1159900 | PREDICTED: pyruvate decarboxylase 2-like [*Malus* *domestica*] |
| MD04G1160100 | PREDICTED: pyruvate decarboxylase 2-like [*Malus* *domestica*] |
| MD04G1167500 | PREDICTED: uncharacterized protein LOC103433716 [*Malus* *domestica*] |
| MD04G1167600 | PREDICTED: CASP-like protein 4B1 [*Malus* *domestica*] |
| MD04G1170900 | PREDICTED: cationic peroxidase 1-like [*Malus* *domestica*] |
| MD04G1172400 | PREDICTED: U-box domain-containing protein 16 [*Malus* *domestica*] |
| MD04G1173600 | lipid transfer protein precursor [*Malus* *domestica*] |
| MD04G1176100 | PREDICTED: serine/threonine-protein kinase AtPK2/AtPK19-like [*Malus* *domestica*] |
| MD04G1185700 | PREDICTED: hydrophobic protein RCI2A-like [*Pyrus* x *bretschneideri*] |
| MD04G1188500 | PREDICTED: ribulose bisphosphate carboxylase small chain clone 512-like [*Pyrus* x *bretschneideri*] |
| MD04G1193000 | PREDICTED: solute carrier family 40 member 3, chloroplastic isoform X1 [*Malus* *domestica*] |
| MD04G1193700 | PREDICTED: LOW QUALITY PROTEIN: peptide chain release factor PrfB2, chloroplastic-like [*Malus* *domestica*] |
| MD04G1197700 | PREDICTED: F-box protein PP2-B15-like [*Malus* *domestica*] |
| MD04G1197900 | PREDICTED: uncharacterized protein LOC103433901 [*Malus* *domestica*] |
| MD04G1199400 | PREDICTED: uncharacterized protein LOC103965293 [*Pyrus* x *bretschneideri*] |
| MD04G1204000 | lipoxygenase [*Malus* *domestica*] |
| MD04G1209300 | PREDICTED: GDSL esterase/lipase At1g54790-like [*Malus* *domestica*] |
| MD04G1210100 | PREDICTED: glutathione synthetase, chloroplastic-like [*Malus* *domestica*] |
| MD04G1220900 | zeta-carotene desaturase, chloroplastic/chromoplastic-like [*Malus* *domestica*] |
| MD04G1224700 | PREDICTED: transcription factor UNE12-like isoform X2 [*Malus* *domestica*] |
| MD04G1233200 | PREDICTED: rust resistance kinase Lr10-like [*Malus* *domestica*] |
| MD04G1236000 | PREDICTED: bidirectional sugar transporter NEC1-like [*Malus* *domestica*] |
| MD04G1236100 | PREDICTED: putative leucine-rich repeat receptor-like serine/threonine-protein kinase At2g19230, partial [*Malus* *domestica*] |
| MD04G1238400 | PREDICTED: receptor-like protein kinase FERONIA [*Pyrus* x *bretschneideri*] |
| MD04G1245800 | PREDICTED: RNA polymerase II transcription factor B subunit 5 [*Malus* *domestica*] |
| MD04G1248100 | PREDICTED: LOW QUALITY PROTEIN: putative metallophosphoesterase At3g03305 [*Malus* *domestica*] |
| MD05G1002900 | PREDICTED: polygalacturonase inhibitor 2-like [*Malus* *domestica*] |
| MD05G1005300 | PREDICTED: CTP synthase-like [*Malus* *domestica*] |
| MD05G1007300 | PREDICTED: uncharacterized protein LOC103956544 isoform X1 [*Pyrus* x *bretschneideri*] |
| MD05G1009200 | PREDICTED: uncharacterized protein LOC103436624 [*Malus* *domestica*] |
| MD05G1017100 | PREDICTED: pathogen-related protein-like [*Pyrus* x *bretschneideri*] |
| MD05G1018200 | PREDICTED: dof zinc finger protein DOF3.5 [*Malus* *domestica*] |
| MD05G1020500 | PREDICTED: U-box domain-containing protein 29-like [*Malus* *domestica*] |
| MD05G1022800 | PREDICTED: TMV resistance protein N-like [*Malus* *domestica*] |
| MD05G1028500 | PREDICTED: glycine-rich cell wall structural protein 1.0-like [*Malus* *domestica*] |
| MD05G1033800 | PREDICTED: tryptophan aminotransferase-related protein 3-like [*Malus* *domestica*] |
| MD05G1034300 | PREDICTED: type IV inositol polyphosphate 5-phosphatase 3 isoform X1 [*Malus* *domestica*] |
| MD05G1035600 | PREDICTED: hypoxanthine-guanine phosphoribosyltransferase-like [*Malus* *domestica*] |
| MD05G1035700 | U-box domain-containing protein 6 [*Prunus persica*] |
| MD05G1038600 | PREDICTED: uncharacterized protein LOC103443899 isoform X2 [*Malus* *domestica*] |
| MD05G1045600 | PREDICTED: dicarboxylate transporter 2, chloroplastic-like, partial [*Pyrus* x *bretschneideri*] |
| MD05G1047300 | PREDICTED: beta carbonic anhydrase 5, chloroplastic isoform X2 [*Malus* *domestica*] |
| MD05G1050100 | PREDICTED: uncharacterized protein LOC103940797 [*Pyrus* x *bretschneideri*] |
| MD05G1050300 | PREDICTED: phosphoglycerate mutase-like protein 1 [*Malus* *domestica*] |
| MD05G1054800 | sorbitol-6-phosphate dehydrogenase 1 [*Malus* *domestica*] |
| MD05G1060700 | PREDICTED: LOW QUALITY PROTEIN: probable carboxylesterase 18 [*Malus* *domestica*] |
| MD05G1069000 | PREDICTED: putative Peroxidase 48 [*Pyrus* x *bretschneideri*] |
| MD05G1072500 | PREDICTED: B3 domain-containing transcription factor VRN1-like isoform X1 [*Malus* *domestica*] |
| MD05G1074200 | PREDICTED: protein DMR6-LIKE OXYGENASE 2-like [*Malus* *domestica*] |
| MD05G1080300 | PREDICTED: putative leucine-rich repeat receptor-like serine/threonine-protein kinase At2g14440 [*Malus* *domestica*] |
| MD05G1081100 | PREDICTED: alpha-amylase-like [*Malus* *domestica*] |
| MD05G1081200 | PREDICTED: alpha-amylase-like [*Malus* *domestica*] |
| MD05G1082700 | PREDICTED: probable receptor-like protein kinase At1g49730 isoform X1 [*Pyrus* x *bretschneideri*] |
| MD05G1083900 | PREDICTED: caffeoyl-CoA O-methyltransferase [*Malus* *domestica*] |
| MD05G1096400 | PREDICTED: somatic embryogenesis receptor kinase 2-like [*Malus* *domestica*] |
| MD05G1097000 | PREDICTED: FAD-linked sulfhydryl oxidase ERV1 [*Malus* *domestica*] |
| MD05G1100200 | PREDICTED: TMV resistance protein N-like isoform X2 [*Malus* *domestica*] |
| MD05G1107500 | PREDICTED: LOW QUALITY PROTEIN: tubulin alpha chain-like [*Malus* *domestica*] |
| MD05G1107600 | PREDICTED: inositol-tetrakisphosphate 1-kinase 3 isoform X1 [*Pyrus* x *bretschneideri*] |
| MD05G1108400 | PREDICTED: uncharacterized protein LOC103436305 [*Malus* *domestica*] |
| MD05G1109800 | -- |
| MD05G1113000 | PREDICTED: transcription factor bHLH18-like [*Malus* *domestica*] |
| MD05G1116000 | PREDICTED: syntaxin-52-like isoform X1 [*Pyrus* x *bretschneideri*] |
| MD05G1118500 | PREDICTED: uncharacterized protein LOC103403603 [*Malus* *domestica*] |
| MD05G1120700 | synaptotagmin-1 [*Prunus persica*] |
| MD05G1122000 | PREDICTED: probable BOI-related E3 ubiquitin-protein ligase 2 [*Malus* *domestica*] |
| MD05G1125100 | PREDICTED: serine/threonine protein phosphatase 2A 57 kDa regulatory subunit B&apos; beta isoform-like [*Malus* *domestica*] |
| MD05G1126400 | PREDICTED: uncharacterized protein LOC103412108 [*Malus* *domestica*] |
| MD05G1128800 | PREDICTED: L-ascorbate oxidase homolog [*Malus* *domestica*] |
| MD05G1135100 | PREDICTED: uncharacterized protein LOC103436201 [*Malus* *domestica*] |
| MD05G1135200 | PREDICTED: transport and Golgi organization protein 2 homolog [*Malus* *domestica*] |
| MD05G1136000 | PREDICTED: uncharacterized protein LOC103452197 isoform X1 [*Malus* *domestica*] |
| MD05G1136300 | PREDICTED: LOW QUALITY PROTEIN: actin cytoskeleton-regulatory complex protein PAN1-like [*Malus* *domestica*] |
| MD05G1138200 | PREDICTED: uncharacterized protein LOC103436010 isoform X2 [*Malus* *domestica*] |
| MD05G1143500 | 3-hydroxy-3-methylglutaryl coenzyme A reductase [*Pyrus* communis] |
| MD05G1154400 | -- |
| MD05G1155900 | PREDICTED: uncharacterized protein LOC103949561 [*Pyrus* x *bretschneideri*] |
| MD05G1156300 | PREDICTED: protein FAR-RED IMPAIRED RESPONSE 1-like isoform X1 [*Pyrus* x *bretschneideri*] |
| MD05G1162300 | PREDICTED: AUGMIN subunit 3-like isoform X2 [*Pyrus* x *bretschneideri*] |
| MD05G1163000 | PREDICTED: uncharacterized protein LOC103435913 isoform X2 [*Malus* *domestica*] |
| MD05G1163900 | PREDICTED: uncharacterized protein LOC103435906 [*Malus* *domestica*] |
| MD05G1169500 | PREDICTED: cyclin-C1-2-like isoform X2 [*Pyrus* x *bretschneideri*] |
| MD05G1170000 | PREDICTED: cytochrome P450 CYP82D47-like [*Malus* *domestica*] |
| MD05G1185600 | PREDICTED: uncharacterized protein LOC103428014 isoform X1 [*Malus* *domestica*] |
| MD05G1189100 | PREDICTED: uncharacterized protein LOC103435761 [*Malus* *domestica*] |
| MD05G1193600 | PREDICTED: glycolipid transfer protein 1-like [*Malus* *domestica*] |
| MD05G1199100 | PREDICTED: protein DOWNY MILDEW RESISTANCE 6-like [*Pyrus* x *bretschneideri*] |
| MD05G1203900 | PREDICTED: uncharacterized protein LOC103435696 [*Malus* *domestica*] |
| MD05G1207000 | PREDICTED: gibberellin 2-beta-dioxygenase 1-like isoform X1 [*Malus* *domestica*] |
| MD05G1208700 | PREDICTED: probable inactive 2-oxoglutarate-dependent dioxygenase AOP2 [*Malus* *domestica*] |
| MD05G1208800 | PREDICTED: coiled-coil domain-containing protein 94 homolog [*Malus* *domestica*] |
| MD05G1209300 | PREDICTED: peroxisomal (S)-2-hydroxy-acid oxidase-like isoform X1 [*Pyrus* x *bretschneideri*] |
| MD05G1213200 | PREDICTED: DNA damage-inducible protein 1 [*Pyrus* x *bretschneideri*] |
| MD05G1223700 | PREDICTED: putative protein TPRXL [*Malus* *domestica*] |
| MD05G1229500 | PREDICTED: DELLA protein RGL1-like [*Malus* *domestica*] |
| MD05G1230700 | PREDICTED: glycine-rich RNA-binding protein 4, mitochondrial isoform X2 [*Malus* *domestica*] |
| MD05G1230900 | PREDICTED: uncharacterized protein LOC103454570 [*Malus* *domestica*] |
| MD05G1234400 | PREDICTED: glyceraldehyde-3-phosphate dehydrogenase GAPCP1, chloroplastic-like [*Malus* *domestica*] |
| MD05G1235400 | PREDICTED: metal tolerance protein 10-like isoform X2 [*Malus* *domestica*] |
| MD05G1236300 | PREDICTED: leucine-rich repeat receptor protein kinase EMS1-like [*Pyrus* x *bretschneideri*] |
| MD05G1238900 | PREDICTED: organic cation/carnitine transporter 3 [*Malus* *domestica*] |
| MD05G1243500 | PREDICTED: uncharacterized protein LOC103435326 [*Malus* *domestica*] |
| MD05G1244500 | PREDICTED: vacuolar-sorting receptor 6-like [*Malus* *domestica*] |
| MD05G1251000 | PREDICTED: glycine cleavage system H protein 2, mitochondrial [*Pyrus* x *bretschneideri*] |
| MD05G1252400 | PREDICTED: glutathionyl-hydroquinone reductase YqjG [*Malus* *domestica*] |
| MD05G1254300 | protein PHLOEM PROTEIN 2-LIKE A9-like [*Malus* *domestica*] |
| MD05G1255300 | PREDICTED: uncharacterized protein LOC103408991 [*Malus* *domestica*] |
| MD05G1259800 | -- |
| MD05G1260500 | PREDICTED: acyl-coenzyme A thioesterase 13-like [*Malus* *domestica*] |
| MD05G1261900 | PREDICTED: protein prune homolog isoform X1 [*Malus* *domestica*] |
| MD05G1264700 | PREDICTED: protein G1-like1 [*Pyrus* x *bretschneideri*] |
| MD05G1267600 | PREDICTED: reticuline oxidase-like protein [*Pyrus* x *bretschneideri*] |
| MD05G1272500 | PREDICTED: GDP-L-galactose phosphorylase 2-like isoform X1 [*Malus* *domestica*] |
| MD05G1276800 | PREDICTED: transcription factor MYB114-like [*Malus* *domestica*] |
| MD05G1277700 | PREDICTED: phospholipase A1-Igamma1, chloroplastic-like [*Malus* *domestica*] |
| MD05G1280000 | PREDICTED: solanesyl diphosphate synthase 3, chloroplastic/mitochondrial-like isoform X2 [*Malus* *domestica*] |
| MD05G1281200 | PREDICTED: F-box protein At5g46170-like [*Malus* *domestica*] |
| MD05G1282000 | PREDICTED: protein TIFY 5A-like [*Malus* *domestica*] |
| MD05G1282700 | PREDICTED: probable 9-cis-epoxycarotenoid dioxygenase NCED5, chloroplastic [*Malus* *domestica*] |
| MD05G1287300 | PREDICTED: tobamovirus multiplication protein 1-like isoform X1 [*Malus* *domestica*] |
| MD05G1289900 | PREDICTED: probable purine permease 9 [*Malus* *domestica*] |
| MD05G1294000 | PREDICTED: MACPF domain-containing protein CAD1 [*Malus* *domestica*] |
| MD05G1296400 | PREDICTED: uncharacterized protein LOC103435110 [*Malus* *domestica*] |
| MD05G1300300 | PREDICTED: peroxisome biogenesis protein 7-like [*Malus* *domestica*] |
| MD05G1301500 | PREDICTED: uncharacterized protein LOC103940720 [*Pyrus* x *bretschneideri*] |
| MD05G1311900 | PREDICTED: auxin-repressed 12.5 kDa protein-like isoform X1 [*Malus* *domestica*] |
| MD05G1313600 | PREDICTED: E3 ubiquitin-protein ligase RMA1H1-like [*Malus* *domestica*] |
| MD05G1317800 | PREDICTED: FHA domain-containing protein PS1 [*Pyrus* x *bretschneideri*] |
| MD05G1323000 | PREDICTED: uncharacterized protein LOC103419671 [*Malus* *domestica*] |
| MD05G1328200 | PREDICTED: G-type lectin S-receptor-like serine/threonine-protein kinase RKS1 [*Malus* *domestica*] |
| MD05G1333700 | PREDICTED: G-type lectin S-receptor-like serine/threonine-protein kinase At1g11410 [*Pyrus* x *bretschneideri*] |
| MD05G1336400 | PREDICTED: thioredoxin-like protein CXXS1 [*Malus* *domestica*] |
| MD05G1336800 | PREDICTED: cysteine-rich receptor-like protein kinase 29 [*Malus* *domestica*] |
| MD05G1336900 | PREDICTED: F-box/LRR-repeat protein At5g63520-like [*Malus* *domestica*] |
| MD05G1337500 | PREDICTED: putative receptor-like protein kinase At4g00960 [*Malus* *domestica*] |
| MD05G1345500 | PREDICTED: uncharacterized protein LOC103434506 [*Malus* *domestica*] |
| MD05G1349800 | PREDICTED: probable WRKY transcription factor 31 [*Malus* *domestica*] |
| MD05G1355300 | protein PHLOEM PROTEIN 2-LIKE A1-like [*Malus* *domestica*] |
| MD05G1355800 | PREDICTED: tropinone reductase-like 3 [*Malus* *domestica*] |
| MD05G1355900 | PREDICTED: uncharacterized membrane protein At1g16860-like isoform X1 [*Malus* *domestica*] |
| MD05G1357200 | PREDICTED: umecyanin-like [*Malus* *domestica*] |
| MD05G1359900 | PREDICTED: inositol transporter 4-like isoform X1 [*Malus* *domestica*] |
| MD05G1362800 | PREDICTED: nuclear inhibitor of protein phosphatase 1-like [*Malus* *domestica*] |
| MD05G1363600 | PREDICTED: ATP-dependent 6-phosphofructokinase 2 [*Pyrus* x *bretschneideri*] |
| MD06G1002600 | PREDICTED: protein PIN-LIKES 3-like [*Pyrus* x *bretschneideri*] |
| MD06G1002700 | PREDICTED: protein PIN-LIKES 3-like [*Malus* *domestica*] |
| MD06G1003500 | PREDICTED: cinnamoyl-CoA reductase 2-like [*Malus* *domestica*] |
| MD06G1003700 | PREDICTED: cinnamoyl-CoA reductase 2-like [*Malus* *domestica*] |
| MD06G1005100 | PREDICTED: monosaccharide-sensing protein 2-like [*Malus* *domestica*] |
| MD06G1010300 | PREDICTED: transport and Golgi organization 2 homolog [*Malus* *domestica*] |
| MD06G1010900 | ABA 8&apos;-hydroxylase 4 [*Pyrus* *pyrifolia*] |
| MD06G1017000 | PREDICTED: acyl-CoA-binding protein isoform X1 [*Malus* *domestica*] |
| MD06G1017700 | PREDICTED: methanol O-anthraniloyltransferase-like [*Pyrus* x *bretschneideri*] |
| MD06G1019200 | alcohol acyltransferase, partial [*Malus* *domestica*] |
| MD06G1020500 | PREDICTED: cysteine--tRNA ligase 2, cytoplasmic-like [*Pyrus* x *bretschneideri*] |
| MD06G1025600 | PREDICTED: probable calcium-binding protein CML11 [*Malus* *domestica*] |
| MD06G1026100 | PREDICTED: lysine-specific demethylase JMJ706-like isoform X1 [*Malus* *domestica*] |
| MD06G1027300 | PREDICTED: probable xyloglucan endotransglucosylase/hydrolase protein 30 [*Malus* *domestica*] |
| MD06G1028600 | PREDICTED: ribose-phosphate pyrophosphokinase 1 [*Malus* *domestica*] |
| MD06G1032200 | PREDICTED: plant intracellular Ras-group-related LRR protein 5-like [*Malus* *domestica*] |
| MD06G1041000 | PREDICTED: expansin-like B1 [*Malus* *domestica*] |
| MD06G1041200 | PREDICTED: haloacid dehalogenase-like hydrolase domain-containing protein Sgpp [*Malus* *domestica*] |
| MD06G1046300 | serine/threonine-protein kinase SAPK3-like [*Malus* *domestica*] |
| MD06G1061100 | PREDICTED: autophagy-related protein 8f [*Malus* *domestica*] |
| MD06G1067000 | PREDICTED: uncharacterized protein LOC103935816 [*Pyrus* x *bretschneideri*] |
| MD06G1070000 | PREDICTED: hydroxymethylglutaryl-CoA synthase-like [*Malus* *domestica*] |
| MD06G1074900 | PREDICTED: maspardin-like [*Malus* *domestica*] |
| MD06G1079100 | PREDICTED: oxalate--CoA ligase-like [*Malus* *domestica*] |
| MD06G1081300 | PREDICTED: probable phospholipid hydroperoxide glutathione peroxidase [*Pyrus* x *bretschneideri*] |
| MD06G1084800 | PREDICTED: probable carotenoid cleavage dioxygenase 4, chloroplastic [*Pyrus* x *bretschneideri*] |
| MD06G1093000 | PREDICTED: respiratory burst oxidase homolog protein A [*Pyrus* x *bretschneideri*] |
| MD06G1100900 | hypothetical protein PRUPE_5G114500 [*Prunus persica*] |
| MD06G1101000 | hypothetical protein PRUPE_5G114500 [*Prunus persica*] |
| MD06G1105300 | probable polygalacturonase [*Prunus persica*] |
| MD06G1109400 | PREDICTED: E3 ubiquitin-protein ligase SINAT4-like [*Pyrus* x *bretschneideri*] |
| MD06G1111300 | PREDICTED: probable E3 ubiquitin-protein ligase RHA4A [*Malus* *domestica*] |
| MD06G1112200 | PREDICTED: uncharacterized protein LOC108175123 [*Malus* *domestica*] |
| MD06G1113500 | PREDICTED: probable trehalase [*Malus* *domestica*] |
| MD06G1118500 | PREDICTED: uncharacterized protein LOC103437424 [*Malus* *domestica*] |
| MD06G1120200 | PREDICTED: transcription factor MYC2-like [*Malus* *domestica*] |
| MD06G1120800 | PREDICTED: protein GPR107-like [*Malus* *domestica*] |
| MD06G1122200 | PREDICTED: uncharacterized protein LOC108867497 [*Pyrus* x *bretschneideri*] |
| MD06G1124100 | PREDICTED: protein DMR6-LIKE OXYGENASE 2-like [*Malus* *domestica*] |
| MD06G1125600 | PREDICTED: nuclear pore complex protein NUP98A-like isoform X3 [*Malus* *domestica*] |
| MD06G1129000 | PREDICTED: chromophore lyase CRL, chloroplastic-like isoform X2 [*Malus* *domestica*] |
| MD06G1135200 | PREDICTED: uncharacterized protein LOC103934197 [*Pyrus* x *bretschneideri*] |
| MD06G1141800 | PREDICTED: F-box protein At1g67340-like [*Malus* *domestica*] |
| MD06G1141900 | PREDICTED: ubiquitin-like modifier-activating enzyme 5 isoform X1 [*Malus* *domestica*] |
| MD06G1143500 | PREDICTED: putative calcium-binding protein CML19 [*Malus* *domestica*] |
| MD06G1144200 | PREDICTED: probable adenylate kinase 7, mitochondrial isoform X1 [*Malus* *domestica*] |
| MD06G1150400 | PREDICTED: pectinesterase PPME1-like [*Pyrus* x *bretschneideri*] |
| MD06G1150500 | PREDICTED: uncharacterized protein LOC103407873 [*Malus* *domestica*] |
| MD06G1156800 | PREDICTED: elongator complex protein 3-like isoform X1 [*Malus* *domestica*] |
| MD06G1160500 | PREDICTED: photosystem II core complex proteins psbY, chloroplastic-like [*Malus* *domestica*] |
| MD06G1160700 | PREDICTED: peptide methionine sulfoxide reductase-like [*Malus* *domestica*] |
| MD06G1165100 | PREDICTED: uncharacterized protein LOC103926780 [*Pyrus* x *bretschneideri*] |
| MD06G1166300 | PREDICTED: L-type lectin-domain containing receptor kinase S.1 [*Malus* *domestica*] |
| MD06G1167600 | PREDICTED: uncharacterized protein LOC103337926 isoform X1 [*Prunus mume*] |
| MD06G1173800 | PREDICTED: probable inactive poly [ADP-ribose] polymerase SRO5 [*Malus* *domestica*] |
| MD06G1178800 | PREDICTED: LIM domain-containing protein WLIM1-like [*Malus* *domestica*] |
| MD06G1179700 | PREDICTED: alpha-mannosidase I MNS5-like isoform X2 [*Malus* *domestica*] |
| MD06G1180300 | PREDICTED: uncharacterized protein At5g50100, mitochondrial-like [*Malus* *domestica*] |
| MD06G1181300 | hypothetical protein PRUPE_5G179600 [*Prunus persica*] |
| MD06G1184300 | PREDICTED: coatomer subunit beta&apos;-1-like [*Malus* *domestica*] |
| MD06G1186000 | PREDICTED: calcium-dependent protein kinase 10-like [*Malus* *domestica*] |
| MD06G1187400 | PREDICTED: (-)-isopiperitenol/(-)-carveol dehydrogenase, mitochondrial-like [*Malus* *domestica*] |
| MD06G1199100 | PREDICTED: flavin-containing monooxygenase FMO GS-OX-like 9 [*Malus* *domestica*] |
| MD06G1204700 | PREDICTED: universal stress protein PHOS32-like [*Malus* *domestica*] |
| MD06G1205700 | PREDICTED: uncharacterized protein LOC103437920 [*Malus* *domestica*] |
| MD06G1207500 | PREDICTED: agmatine deiminase [*Malus* *domestica*] |
| MD06G1208200 | PREDICTED: plant cysteine oxidase 3 [*Malus* *domestica*] |
| MD06G1213500 | PREDICTED: cytochrome P450 CYP736A12-like [*Malus* *domestica*] |
| MD06G1213800 | PREDICTED: cytochrome P450 CYP736A12-like [*Malus* *domestica*] |
| MD06G1214400 | PREDICTED: cytochrome P450 CYP736A12-like [*Malus* *domestica*] |
| MD06G1217200 | PREDICTED: transcription factor WER [*Malus* *domestica*] |
| MD06G1217400 | PREDICTED: uncharacterized protein LOC103438012 [*Malus* *domestica*] |
| MD06G1223900 | PREDICTED: F-box/LRR-repeat protein 3-like [*Malus* *domestica*] |
| MD06G1225600 | PREDICTED: glycerophosphodiester phosphodiesterase GDPD6-like [*Malus* *domestica*] |
| MD06G1226600 | PREDICTED: uncharacterized protein LOC103428325 isoform X1 [*Malus* *domestica*] |
| MD06G1230600 | PREDICTED: LOW QUALITY PROTEIN: peptidyl-prolyl cis-trans isomerase FKBP17-2, chloroplastic-like [*Malus* *domestica*] |
| MD06G1231000 | PREDICTED: probable calcium-binding protein CML27 [*Malus* *domestica*] |
| MD06G1232300 | hypothetical protein PRUPE_5G236600 [*Prunus persica*] |
| MD06G1233600 | PREDICTED: DNA-directed RNA polymerase II subunit 4-like [*Malus* *domestica*] |
| MD06G1237600 | PREDICTED: uncharacterized protein At4g15970-like isoform X3 [*Malus* *domestica*] |
| MD06G1238500 | PREDICTED: retinol dehydrogenase 12-like [*Malus* *domestica*] |
| MD07G1001800 | PREDICTED: callose synthase 1 [*Pyrus* x *bretschneideri*] |
| MD07G1004400 | PREDICTED: uncharacterized protein At1g04910-like [*Malus* *domestica*] |
| MD07G1004600 | lipoxygenase [*Malus* *domestica*] |
| MD07G1008600 | PREDICTED: thylakoid lumenal 19 kDa protein, chloroplastic [*Malus* *domestica*] |
| MD07G1009400 | PREDICTED: uncharacterized protein LOC103438396 [*Malus* *domestica*] |
| MD07G1011000 | PREDICTED: uncharacterized protein LOC103438504 [*Malus* *domestica*] |
| MD07G1017500 | PREDICTED: uncharacterized protein LOC103443604 [*Malus* *domestica*] |
| MD07G1019700 | PREDICTED: putative disease resistance protein RGA3 [*Malus* *domestica*] |
| MD07G1023400 | PREDICTED: uncharacterized protein LOC103428386 isoform X2 [*Malus* *domestica*] |
| MD07G1024300 | PREDICTED: disease resistance protein RGA2-like [*Malus* *domestica*] |
| MD07G1025000 | PREDICTED: serine/threonine-protein kinase-like protein At3g51990 [*Pyrus* x *bretschneideri*] |
| MD07G1028400 | PREDICTED: probable inactive purple acid phosphatase 27 [*Malus* *domestica*] |
| MD07G1029700 | PREDICTED: probable inactive purple acid phosphatase 27 [*Malus* *domestica*] |
| MD07G1033200 | PREDICTED: xanthoxin dehydrogenase isoform X2 [*Malus* *domestica*] |
| MD07G1041100 | PREDICTED: cytochrome c oxidase assembly protein COX11, mitochondrial-like [*Pyrus* x *bretschneideri*] |
| MD07G1052300 | PREDICTED: uncharacterized protein LOC103438742 [*Malus* *domestica*] |
| MD07G1053700 | PREDICTED: uncharacterized protein LOC103447797 [*Malus* *domestica*] |
| MD07G1062200 | PREDICTED: uricase-2 isozyme 1-like [*Malus* *domestica*] |
| MD07G1066200 | copper transport protein ATX1 [*Prunus persica*] |
| MD07G1068400 | PREDICTED: uncharacterized protein LOC103438808 [*Malus* *domestica*] |
| MD07G1069900 | -- |
| MD07G1072800 | PREDICTED: uncharacterized protein LOC103951720 [*Pyrus* x *bretschneideri*] |
| MD07G1073600 | PREDICTED: malate dehydrogenase [NADP], chloroplastic-like [*Malus* *domestica*] |
| MD07G1079200 | PREDICTED: disease resistance protein RML1A-like [*Malus* *domestica*] |
| MD07G1082500 | PREDICTED: uncharacterized protein LOC103438906 [*Malus* *domestica*] |
| MD07G1082700 | PREDICTED: protein NRT1/ PTR FAMILY 7.3-like isoform X1 [*Pyrus* x *bretschneideri*] |
| MD07G1083900 | PREDICTED: WD-40 repeat-containing protein MSI1-like [*Malus* *domestica*] |
| MD07G1084700 | PREDICTED: uncharacterized protein LOC103942790 isoform X2 [*Pyrus* x *bretschneideri*] |
| MD07G1095400 | PREDICTED: AP-1 complex subunit mu-2-like [*Malus* *domestica*] |
| MD07G1101300 | PREDICTED: flowering locus K homology domain-like [*Malus* *domestica*] |
| MD07G1118800 | PREDICTED: ycf20-like protein [*Malus* *domestica*] |
| MD07G1121000 | PREDICTED: ATP-dependent DNA helicase 2 subunit KU80-like [*Malus* *domestica*] |
| MD07G1124400 | PREDICTED: glyoxylate/hydroxypyruvate reductase HPR3-like [*Malus* *domestica*] |
| MD07G1130700 | PREDICTED: uncharacterized protein LOC103439160 [*Malus* *domestica*] |
| MD07G1130800 | PREDICTED: desiccation protectant protein Lea14 homolog [*Malus* *domestica*] |
| MD07G1130900 | PREDICTED: desiccation protectant protein Lea14 homolog [*Malus* *domestica*] |
| MD07G1132000 | PREDICTED: uncharacterized protein LOC103410155 [*Malus* *domestica*] |
| MD07G1132300 | PREDICTED: LOW QUALITY PROTEIN: uncharacterized protein At1g01500-like [*Malus* *domestica*] |
| MD07G1132700 | PREDICTED: 50S ribosomal protein L5, chloroplastic-like [*Malus* *domestica*] |
| MD07G1135000 | L-3-cyanoalanine synthase 1, mitochondrial [*Malus* *domestica*] |
| MD07G1139300 | PREDICTED: leucine-rich repeat receptor protein kinase MSP1-like isoform X2 [*Pyrus* x *bretschneideri*] |
| MD07G1139600 | PREDICTED: AT-hook motif nuclear-localized protein 9 [*Malus* *domestica*] |
| MD07G1144600 | PREDICTED: mitochondrial carrier protein MTM1-like [*Malus* *domestica*] |
| MD07G1144900 | PREDICTED: arabinogalactan peptide 20-like [*Malus* *domestica*] |
| MD07G1145400 | -- |
| MD07G1146600 | -- |
| MD07G1147100 | PREDICTED: transcription factor CPC-like [*Malus* *domestica*] |
| MD07G1148500 | PREDICTED: mitochondrial inner membrane protein OXA1-like [*Malus* *domestica*] |
| MD07G1154500 | PREDICTED: LOW QUALITY PROTEIN: serine/arginine-rich splicing factor RS31A [*Malus* *domestica*] |
| MD07G1169000 | PREDICTED: FGGY carbohydrate kinase domain-containing protein-like [*Malus* *domestica*] |
| MD07G1171600 | PREDICTED: OTU domain-containing protein DDB_G0284757-like isoform X1 [*Malus* *domestica*] |
| MD07G1171700 | PREDICTED: ATPase ASNA1 homolog [*Malus* *domestica*] |
| MD07G1172700 | PREDICTED: phenylalanine ammonia-lyase 1 [*Malus* *domestica*] |
| MD07G1174800 | PREDICTED: caffeoylshikimate esterase-like [*Malus* *domestica*] |
| MD07G1176500 | PREDICTED: serine carboxypeptidase-like 27 [*Malus* *domestica*] |
| MD07G1178100 | PREDICTED: BAG family molecular chaperone regulator 5, mitochondrial-like [*Malus* *domestica*] |
| MD07G1185400 | PREDICTED: uncharacterized protein LOC108868264 [*Pyrus* x *bretschneideri*] |
| MD07G1185600 | PREDICTED: GTP-binding nuclear protein Ran1B-like [Cucumis sativus] |
| MD07G1187200 | PREDICTED: 3-ketoacyl-CoA synthase 19-like [*Pyrus* x *bretschneideri*] |
| MD07G1187400 | PREDICTED: 3-ketoacyl-CoA synthase 19-like [*Pyrus* x *bretschneideri*] |
| MD07G1189600 | PREDICTED: uncharacterized protein LOC103434533 [*Malus* *domestica*] |
| MD07G1189900 | PREDICTED: probable sulfate transporter 4.2 isoform X2 [*Malus* *domestica*] |
| MD07G1191700 | PREDICTED: putative nuclease HARBI1 [*Pyrus* x *bretschneideri*] |
| MD07G1191800 | PREDICTED: actin-depolymerizing factor 1 [*Malus* *domestica*] |
| MD07G1192900 | PREDICTED: zinc finger protein ZAT12-like [*Malus* *domestica*] |
| MD07G1193800 | PREDICTED: probable protein phosphatase 2C 78 [*Malus* *domestica*] |
| MD07G1196500 | heat shock cognate 70 kDa protein 2-like [*Malus* *domestica*] |
| MD07G1196700 | PREDICTED: heat shock cognate 70 kDa protein-like [*Malus* *domestica*] |
| MD07G1199400 | PREDICTED: probable serine/threonine-protein kinase NAK [*Malus* *domestica*] |
| MD07G1210800 | PREDICTED: 16.9 kDa class I heat shock protein 2-like [*Malus* *domestica*] |
| MD07G1211400 | PREDICTED: uncharacterized protein LOC103942664 [*Pyrus* x *bretschneideri*] |
| MD07G1211800 | PREDICTED: galactose-binding lectin-like [*Malus* *domestica*] |
| MD07G1221400 | PREDICTED: LOW QUALITY PROTEIN: putative ABC1 protein At2g40090 [*Malus* *domestica*] |
| MD07G1227000 | PREDICTED: protein trichome birefringence-like 33 isoform X2 [*Malus* *domestica*] |
| MD07G1228300 | PREDICTED: biotin carboxyl carrier protein of acetyl-CoA carboxylase-like isoform X2 [*Malus* *domestica*] |
| MD07G1228500 | PREDICTED: protein EARLY RESPONSIVE TO DEHYDRATION 15-like [*Malus* *domestica*] |
| MD07G1229000 | PREDICTED: uncharacterized protein LOC103432881 [*Malus* *domestica*] |
| MD07G1233600 | PREDICTED: uncharacterized protein LOC103432918 [*Malus* *domestica*] |
| MD07G1234900 | hypothetical protein PRUPE_2G265200 [*Prunus persica*] |
| MD07G1237000 | PREDICTED: methylesterase 17-like [*Malus* *domestica*] |
| MD07G1238800 | PREDICTED: cytochrome P450 714A1-like [*Malus* *domestica*] |
| MD07G1243600 | ATP-dependent DNA helicase SRS2-like protein At4g25120 isoform X2 [*Prunus persica*] |
| MD07G1249500 | PREDICTED: acid phosphatase 1 [*Malus* *domestica*] |
| MD07G1249600 | PREDICTED: putative kinase-like protein TMKL1 [*Malus* *domestica*] |
| MD07G1249700 | PREDICTED: U-box domain-containing protein 35 isoform X1 [*Malus* *domestica*] |
| MD07G1249800 | PREDICTED: U-box domain-containing protein 52-like [*Malus* *domestica*] |
| MD07G1253400 | hypothetical protein PRUPE_6G011700 [*Prunus persica*] |
| MD07G1258400 | PREDICTED: uncharacterized protein LOC103439669 [*Malus* *domestica*] |
| MD07G1263200 | ethylene-responsive transcription factor ERF027-like [*Malus* *domestica*] |
| MD07G1268200 | PREDICTED: remorin-like [*Malus* *domestica*] |
| MD07G1272700 | PREDICTED: pectin acetylesterase 9 isoform X2 [*Malus* *domestica*] |
| MD07G1278700 | PREDICTED: putative septum site-determining protein minD homolog, chloroplastic [*Pyrus* x *bretschneideri*] |
| MD07G1286100 | PREDICTED: probable trehalose-phosphate phosphatase 4 isoform X1 [*Malus* *domestica*] |
| MD07G1287700 | PREDICTED: starch synthase 1, chloroplastic/amyloplastic-like isoform X1 [*Malus* *domestica*] |
| MD07G1296500 | PREDICTED: cytochrome b5 [*Malus* *domestica*] |
| MD07G1297100 | homeobox-leucine zipper protein ATHB-12-like [*Malus* *domestica*] |
| MD07G1297400 | PREDICTED: auxin-responsive protein SAUR32 [*Malus* *domestica*] |
| MD08G1002300 | PREDICTED: 1,4-alpha-glucan-branching enzyme 1, chloroplastic/amyloplastic-like [*Malus* *domestica*] |
| MD08G1009800 | PREDICTED: GDSL esterase/lipase EXL3-like [*Malus* *domestica*] |
| MD08G1014800 | PREDICTED: ATP sulfurylase 2 [*Pyrus* x *bretschneideri*] |
| MD08G1017500 | PREDICTED: 1-acylglycerol-3-phosphate O-acyltransferase-like [*Malus* *domestica*] |
| MD08G1028000 | PREDICTED: pentatricopeptide repeat-containing protein At1g01970 [*Pyrus* x *bretschneideri*] |
| MD08G1028100 | endo-beta-mannanase, partial [*Malus* *domestica*] |
| MD08G1032200 | PREDICTED: peroxisomal membrane protein 11A-like [*Malus* *domestica*] |
| MD08G1038900 | PREDICTED: wall-associated receptor kinase-like 14 [*Malus* *domestica*] |
| MD08G1040600 | PREDICTED: serpin-ZX-like [*Malus* *domestica*] |
| MD08G1042400 | PREDICTED: probable disease resistance protein At4g33300 [*Malus* *domestica*] |
| MD08G1043100 | PREDICTED: calcineurin B-like protein 10 [*Malus* *domestica*] |
| MD08G1043900 | PREDICTED: serine/threonine protein phosphatase 2A 55 kDa regulatory subunit B alpha isoform-like isoform X8 [*Pyrus* x *bretschneideri*] |
| MD08G1045600 | PREDICTED: copper transporter 5.1-like [*Malus* *domestica*] |
| MD08G1047600 | PREDICTED: glutamate receptor 2.7-like [*Malus* *domestica*] |
| MD08G1055100 | PREDICTED: putative glutathione peroxidase 7, chloroplastic [*Malus* *domestica*] |
| MD08G1060000 | PREDICTED: ethylene-responsive transcription factor ERF003 [*Malus* *domestica*] |
| MD08G1064700 | PREDICTED: graves disease carrier protein-like [*Pyrus* x *bretschneideri*] |
| MD08G1066000 | PREDICTED: LOW QUALITY PROTEIN: peptidyl-prolyl cis-trans isomerase CYP57-like [*Malus* *domestica*] |
| MD08G1066400 | PREDICTED: aminopeptidase M1-like [*Malus* *domestica*] |
| MD08G1078800 | PREDICTED: basic leucine zipper 43-like [*Malus* *domestica*] |
| MD08G1080400 | PREDICTED: uncharacterized protein LOC103428750 isoform X1 [*Malus* *domestica*] |
| MD08G1080900 | PREDICTED: NADH dehydrogenase [ubiquinone] iron-sulfur protein 4, mitochondrial-like [*Malus* *domestica*] |
| MD08G1086300 | PREDICTED: adenine phosphoribosyltransferase 1, chloroplastic-like [*Pyrus* x *bretschneideri*] |
| MD08G1088800 | PREDICTED: uncharacterized protein LOC103441068 [*Malus* *domestica*] |
| MD08G1096000 | PREDICTED: ethylene-responsive transcription factor ERF011-like [*Malus* *domestica*] |
| MD08G1101600 | PREDICTED: peroxisomal multifunctional enzyme A-like [*Malus* *domestica*] |
| MD08G1101700 | PREDICTED: alpha-amylase-like [*Pyrus* x *bretschneideri*] |
| MD08G1104000 | PREDICTED: probable carboxylesterase 2 [*Pyrus* x *bretschneideri*] |
| MD08G1109700 | PREDICTED: ATP-dependent 6-phosphofructokinase 6-like [*Pyrus* x *bretschneideri*] |
| MD08G1112100 | PREDICTED: lachrymatory-factor synthase [*Malus* *domestica*] |
| MD08G1117900 | PREDICTED: coiled-coil domain-containing protein 25-like [*Malus* *domestica*] |
| MD08G1118000 | PREDICTED: uncharacterized protein LOC103410743 [*Malus* *domestica*] |
| MD08G1121600 | PREDICTED: flavonol synthase/flavanone 3-hydroxylase [*Pyrus* x *bretschneideri*] |
| MD08G1124400 | PREDICTED: L-arabinokinase-like isoform X2 [*Malus* *domestica*] |
| MD08G1124700 | PREDICTED: putative glutathione-specific gamma-glutamylcyclotransferase 2 [*Malus* *domestica*] |
| MD08G1126500 | PREDICTED: probable sarcosine oxidase [*Malus* *domestica*] |
| MD08G1127900 | PREDICTED: F-box protein AFR-like [*Malus* *domestica*] |
| MD08G1128200 | PREDICTED: uncharacterized protein LOC103410803 [*Malus* *domestica*] |
| MD08G1130200 | PREDICTED: plasma membrane ATPase 4-like [*Malus* *domestica*] |
| MD08G1134900 | PREDICTED: uncharacterized protein LOC103441384 [*Malus* *domestica*] |
| MD08G1135400 | PREDICTED: molybdate transporter 1 [*Malus* *domestica*] |
| MD08G1143000 | PREDICTED: cytochrome b-c1 complex subunit 7-2-like [*Malus* *domestica*] |
| MD08G1143200 | PREDICTED: uncharacterized protein LOC103456171 [*Malus* *domestica*] |
| MD08G1144500 | -- |
| MD08G1148300 | PREDICTED: probable sugar phosphate/phosphate translocator At5g25400 [*Malus* *domestica*] |
| MD08G1168600 | flavonol synthase [*Malus* *domestica*] |
| MD08G1171700 | -- |
| MD08G1174800 | PREDICTED: probable glycerol-3-phosphate acyltransferase 3 [*Malus* *domestica*] |
| MD08G1183000 | PREDICTED: signal peptidase complex-like protein DTM1 [*Pyrus* x *bretschneideri*] |
| MD08G1184000 | PREDICTED: 14-3-3 protein 7-like isoform X2 [*Pyrus* x *bretschneideri*] |
| MD08G1186100 | PREDICTED: protein EXORDIUM-like [*Pyrus* x *bretschneideri*] |
| MD08G1186800 | PREDICTED: F-box protein At1g78280 isoform X2 [*Malus* *domestica*] |
| MD08G1188600 | PREDICTED: queuine tRNA-ribosyltransferase-like [*Malus* *domestica*] |
| MD08G1189800 | PREDICTED: uncharacterized protein LOC103442526 isoform X1 [*Malus* *domestica*] |
| MD08G1195000 | PREDICTED: phosphoribosylformylglycinamidine cyclo-ligase, chloroplastic/mitochondrial-like [*Malus* *domestica*] |
| MD08G1195600 | PREDICTED: probable pectinesterase/pectinesterase inhibitor 20 [*Malus* *domestica*] |
| MD08G1197900 | PREDICTED: protein transport protein SFT2-like [*Malus* *domestica*] |
| MD08G1208000 | PREDICTED: phosphoglycerate mutase-like protein 1 isoform X1 [*Pyrus* x *bretschneideri*] |
| MD08G1216500 | agamous-like MADS-box protein AGL11 [*Malus* *domestica*] |
| MD08G1220800 | PREDICTED: 3-hydroxyacyl-[acyl-carrier-protein] dehydratase FabZ-like [*Malus* *domestica*] |
| MD08G1224000 | PREDICTED: uncharacterized protein LOC103445080 [*Malus* *domestica*] |
| MD08G1227400 | PREDICTED: uncharacterized protein LOC103428932 [*Malus* *domestica*] |
| MD08G1232200 | PREDICTED: 11S globulin seed storage protein 2-like [*Pyrus* x *bretschneideri*] |
| MD08G1232700 | PREDICTED: probable isoaspartyl peptidase/L-asparaginase 3 isoform X1 [*Pyrus* x *bretschneideri*] |
| MD08G1240400 | PREDICTED: nifU-like protein 2, chloroplastic [*Malus* *domestica*] |
| MD08G1247500 | PREDICTED: NADPH-dependent pterin aldehyde reductase-like [*Malus* *domestica*] |
| MD09G1000400 | PREDICTED: acetyl-coenzyme A synthetase, chloroplastic/glyoxysomal isoform X1 [*Malus* *domestica*] |
| MD09G1010200 | PREDICTED: 50S ribosomal protein L27, chloroplastic-like [*Malus* *domestica*] |
| MD09G1013100 | PREDICTED: triacylglycerol lipase 2 [*Pyrus* x *bretschneideri*] |
| MD09G1013500 | PREDICTED: UPF0481 protein At3g47200-like [*Pyrus* x *bretschneideri*] |
| MD09G1020200 | PREDICTED: protein ABHD17C-like [*Malus* *domestica*] |
| MD09G1024400 | PREDICTED: uncharacterized protein LOC103421573 isoform X3 [*Malus* *domestica*] |
| MD09G1025600 | PREDICTED: TMV resistance protein N-like isoform X2 [*Pyrus* x *bretschneideri*] |
| MD09G1027300 | PREDICTED: uncharacterized protein LOC103421468 [*Malus* *domestica*] |
| MD09G1029600 | PREDICTED: phosphoenolpyruvate/phosphate translocator 2, chloroplastic-like isoform X1 [*Malus* *domestica*] |
| MD09G1035100 | homeobox-leucine zipper protein HAT5-like [*Malus* *domestica*] |
| MD09G1036100 | PREDICTED: uncharacterized protein LOC103963933 [*Pyrus* x *bretschneideri*] |
| MD09G1037400 | PREDICTED: protein ALTERED XYLOGLUCAN 4-like isoform X1 [*Malus* *domestica*] |
| MD09G1042100 | PREDICTED: type I inositol polyphosphate 5-phosphatase 4-like isoform X1 [*Malus* *domestica*] |
| MD09G1051000 | myb-related protein 306 [*Malus* *domestica*] |
| MD09G1051100 | PREDICTED: mini zinc finger protein 2 [*Malus* *domestica*] |
| MD09G1054200 | PREDICTED: cystathionine gamma-synthase 1, chloroplastic-like [*Malus* *domestica*] |
| MD09G1055000 | PREDICTED: uncharacterized protein LOC103421504 [*Malus* *domestica*] |
| MD09G1057400 | PREDICTED: phosphoglycerate mutase-like protein 1 [*Malus* *domestica*] |
| MD09G1064200 | PREDICTED: biotin carboxyl carrier protein of acetyl-CoA carboxylase 2, chloroplastic [*Malus* *domestica*] |
| MD09G1068700 | PREDICTED: protein SPIRAL1-like 2, partial [*Pyrus* x *bretschneideri*] |
| MD09G1069500 | probable linoleate 9S-lipoxygenase 5 [*Malus* *domestica*] |
| MD09G1069800 | PREDICTED: septum-promoting GTP-binding protein 1 [*Malus* *domestica*] |
| MD09G1073100 | PREDICTED: PI-PLC X domain-containing protein At5g67130-like [*Malus* *domestica*] |
| MD09G1075400 | PREDICTED: uncharacterized protein LOC103411228 [*Malus* *domestica*] |
| MD09G1079800 | PREDICTED: S-adenosylmethionine decarboxylase proenzyme-like [*Malus* *domestica*] |
| MD09G1083200 | PREDICTED: cyclin-B1-2-like [*Pyrus* x *bretschneideri*] |
| MD09G1088000 | PREDICTED: uncharacterized protein LOC103947825 [*Pyrus* x *bretschneideri*] |
| MD09G1091900 | PREDICTED: uncharacterized protein LOC103947847 [*Pyrus* x *bretschneideri*] |
| MD09G1095200 | PREDICTED: receptor-like serine/threonine-protein kinase SD1-8 [*Pyrus* x *bretschneideri*] |
| MD09G1104300 | PREDICTED: uncharacterized protein LOC103442579 [*Malus* *domestica*] |
| MD09G1108700 | PREDICTED: uncharacterized protein LOC103442921 [*Malus* *domestica*] |
| MD09G1108800 | PREDICTED: aconitate hydratase, cytoplasmic [*Malus* *domestica*] |
| MD09G1109600 | PREDICTED: 2-alkenal reductase (NADP(+)-dependent)-like [*Pyrus* x *bretschneideri*] |
| MD09G1114000 | PREDICTED: palmitoyl-monogalactosyldiacylglycerol delta-7 desaturase, chloroplastic-like [*Malus* *domestica*] |
| MD09G1114100 | PREDICTED: palmitoyl-monogalactosyldiacylglycerol delta-7 desaturase, chloroplastic-like [*Malus* *domestica*] |
| MD09G1116500 | PREDICTED: GDSL esterase/lipase At2g04570-like [*Malus* *domestica*] |
| MD09G1119000 | PREDICTED: fimbrin-5-like [*Malus* *domestica*] |
| MD09G1119600 | PREDICTED: uncharacterized protein LOC103444613 [*Malus* *domestica*] |
| MD09G1131400 | PREDICTED: probable E3 ubiquitin-protein ligase XERICO [*Malus* *domestica*] |
| MD09G1132200 | PREDICTED: subtilisin-like protease SBT5.3 isoform X2 [*Malus* *domestica*] |
| MD09G1137600 | PREDICTED: cysteine-rich and transmembrane domain-containing protein B-like isoform X1 [*Pyrus* x *bretschneideri*] |
| MD09G1140700 | uDP-glycosyltransferase 71A15 [*Malus* *domestica*] |
| MD09G1147900 | PREDICTED: uncharacterized protein LOC103957777 [*Pyrus* x *bretschneideri*] |
| MD09G1149300 | PREDICTED: aspartyl protease family protein 2 isoform X1 [*Malus* *domestica*] |
| MD09G1150700 | PREDICTED: probable WRKY transcription factor 71 [*Pyrus* x *bretschneideri*] |
| MD09G1152600 | brassinosteroid-regulated protein BRU1-like precursor [*Malus* *domestica*] |
| MD09G1152700 | PREDICTED: xyloglucan endotransglucosylase/hydrolase 2-like [*Malus* *domestica*] |
| MD09G1156400 | PREDICTED: citrate synthase, glyoxysomal [*Malus* *domestica*] |
| MD09G1156500 | PREDICTED: V-type proton ATPase subunit D-like [*Malus* *domestica*] |
| MD09G1163100 | PREDICTED: 23 kDa jasmonate-induced protein-like [*Malus* *domestica*] |
| MD09G1174500 | PREDICTED: GDSL esterase/lipase 5-like [*Malus* *domestica*] |
| MD09G1176500 | PREDICTED: dnaJ homolog subfamily B member 7-like isoform X1 [*Malus* *domestica*] |
| MD09G1178600 | PREDICTED: protein TIFY 6b-like [*Malus* *domestica*] |
| MD09G1183900 | PREDICTED: ubiquitin-like protein ATG12 [*Malus* *domestica*] |
| MD09G1187300 | PREDICTED: pollen-specific leucine-rich repeat extensin-like protein 1 [*Malus* *domestica*] |
| MD09G1187500 | PREDICTED: protein YLS9-like [*Malus* *domestica*] |
| MD09G1192200 | PREDICTED: putative hydrolase C777.06c [*Malus* *domestica*] |
| MD09G1193000 | PREDICTED: protein YLS9 [*Malus* *domestica*] |
| MD09G1197500 | PREDICTED: jacalin-related lectin 19-like [*Malus* *domestica*] |
| MD09G1197800 | PREDICTED: IAA-amino acid hydrolase ILR1-like 4 [*Malus* *domestica*] |
| MD09G1199000 | hypothetical protein PRUPE_6G069400 [*Prunus persica*] |
| MD09G1199900 | PREDICTED: protein CHUP1, chloroplastic isoform X1 [*Malus* *domestica*] |
| MD09G1201400 | profilin [*Malus* *domestica*] |
| MD09G1206900 | PREDICTED: LOW QUALITY PROTEIN: cytochrome c oxidase assembly protein COX19-like [*Malus* *domestica*] |
| MD09G1211900 | -- |
| MD09G1216200 | hypothetical protein PRUPE_3G075000 [*Prunus persica*] |
| MD09G1218700 | PREDICTED: uncharacterized protein LOC103935383 [*Pyrus* x *bretschneideri*] |
| MD09G1224500 | PREDICTED: probable WRKY transcription factor 40 [*Malus* *domestica*] |
| MD09G1227900 | callose synthase 9 isoform X2 [*Prunus persica*] |
| MD09G1228500 | PREDICTED: AP2-like ethylene-responsive transcription factor At1g16060 isoform X2 [*Malus* *domestica*] |
| MD09G1241900 | PREDICTED: protein E6 [*Malus* *domestica*] |
| MD09G1246100 | PREDICTED: uncharacterized protein LOC103440258 [*Malus* *domestica*] |
| MD09G1251300 | PREDICTED: lanC-like protein GCL2 [*Malus* *domestica*] |
| MD09G1251400 | -- |
| MD09G1254000 | PREDICTED: probable S-adenosylmethionine-dependent methyltransferase At5g38100 [*Malus* *domestica*] |
| MD09G1255400 | PREDICTED: uncharacterized protein LOC103948482 [*Pyrus* x *bretschneideri*] |
| MD09G1257800 | -- |
| MD09G1257900 | -- |
| MD09G1263000 | PREDICTED: protein OPI10 homolog [*Malus* *domestica*] |
| MD09G1265000 | PREDICTED: ABC transporter B family member 19-like [*Malus* *domestica*] |
| MD09G1270200 | PREDICTED: COX assembly mitochondrial protein 2 homolog isoform X3 [*Malus* *domestica*] |
| MD09G1274400 | PREDICTED: disease resistance protein At4g27190-like [*Pyrus* x *bretschneideri*] |
| MD09G1275700 | PREDICTED: uncharacterized protein LOC103444370 [*Malus* *domestica*] |
| MD09G1278200 | PREDICTED: transcription elongation factor SPT4 homolog 1-like [*Malus* *domestica*] |
| MD09G1278600 | MYB transcription factor [*Malus* *domestica*] |
| MD09G1279300 | PREDICTED: protein REVEILLE 1-like [*Malus* *domestica*] |
| MD09G1285900 | PREDICTED: probable glycerol-3-phosphate acyltransferase 3 [*Malus* *domestica*] |
| MD09G1292300 | PREDICTED: protein RADIALIS-like 6 [*Pyrus* x *bretschneideri*] |
| MD10G1002500 | PREDICTED: probable sucrose-phosphate synthase 4 [*Malus* *domestica*] |
| MD10G1005000 | PREDICTED: uncharacterized protein LOC103421996 [*Malus* *domestica*] |
| MD10G1007500 | PREDICTED: TMV resistance protein N-like, partial [*Malus* *domestica*] |
| MD10G1007600 | PREDICTED: TMV resistance protein N-like, partial [*Malus* *domestica*] |
| MD10G1007900 | PREDICTED: uncharacterized protein LOC103951109 [*Pyrus* x *bretschneideri*] |
| MD10G1017700 | PREDICTED: peptidyl-prolyl cis-trans isomerase CYP63-like [*Malus* *domestica*] |
| MD10G1023900 | PREDICTED: THO complex subunit 7A-like [*Pyrus* x *bretschneideri*] |
| MD10G1029100 | uncharacterized protein LOC109803890 [Cajanus cajan] |
| MD10G1032000 | ethylene-responsive transcription factor ABR1-like [*Malus* *domestica*] |
| MD10G1033300 | PREDICTED: peroxidase 12-like [*Malus* *domestica*] |
| MD10G1049900 | PREDICTED: ABC transporter G family member 11-like [*Malus* *domestica*] |
| MD10G1053100 | PREDICTED: protein DMR6-LIKE OXYGENASE 1-like [*Pyrus* x *bretschneideri*] |
| MD10G1058000 | PREDICTED: glutamyl-tRNA reductase 1, chloroplastic-like [*Malus* *domestica*] |
| MD10G1061900 | PREDICTED: amidophosphoribosyltransferase, chloroplastic [*Malus* *domestica*] |
| MD10G1062300 | NADP-dependent D-sorbitol-6-phosphate dehydrogenase [*Malus* *domestica*] |
| MD10G1062400 | PREDICTED: V-type proton ATPase 16 kDa proteolipid subunit [*Pyrus* x *bretschneideri*] |
| MD10G1066900 | PREDICTED: cysteine protease RD19A [*Malus* *domestica*] |
| MD10G1074400 | PREDICTED: protein RADIALIS-like 5 [*Malus* *domestica*] |
| MD10G1076700 | PREDICTED: probable receptor-like serine/threonine-protein kinase At4g34500 [*Malus* *domestica*] |
| MD10G1080100 | PREDICTED: polyol transporter 5-like isoform X2 [*Malus* *domestica*] |
| MD10G1086100 | PREDICTED: extra-large guanine nucleotide-binding protein 1-like [*Malus* *domestica*] |
| MD10G1091600 | PREDICTED: LOW QUALITY PROTEIN: probable carboxylesterase 12 [*Malus* *domestica*] |
| MD10G1105800 | PREDICTED: adenylyl-sulfate kinase 3-like [*Malus* *domestica*] |
| MD10G1107500 | -- |
| MD10G1110000 | PREDICTED: phytosulfokines-like [*Pyrus* x *bretschneideri*] |
| MD10G1111300 | PREDICTED: UDP-glycosyltransferase 74E1-like [*Pyrus* x *bretschneideri*] |
| MD10G1129900 | PREDICTED: mechanosensitive ion channel protein 6 [*Malus* *domestica*] |
| MD10G1132000 | PREDICTED: L-ascorbate oxidase homolog [*Malus* *domestica*] |
| MD10G1137800 | PREDICTED: uncharacterized protein ycf39-like isoform X3 [*Malus* *domestica*] |
| MD10G1139000 | PREDICTED: sorcin-like isoform X1 [*Malus* *domestica*] |
| MD10G1139800 | PREDICTED: Niemann-Pick C1 protein [*Pyrus* x *bretschneideri*] |
| MD10G1142100 | PREDICTED: monosaccharide-sensing protein 2-like [*Malus* *domestica*] |
| MD10G1146300 | PREDICTED: ribonuclease 2-like [*Malus* *domestica*] |
| MD10G1157100 | PREDICTED: putative quinone-oxidoreductase homolog, chloroplastic [*Malus* *domestica*] |
| MD10G1166300 | PREDICTED: protein SULFUR DEFICIENCY-INDUCED 1-like [*Malus* *domestica*] |
| MD10G1172300 | PREDICTED: probable glutathione S-transferase [*Malus* *domestica*] |
| MD10G1175000 | PREDICTED: pleiotropic drug resistance protein 1-like [*Malus* *domestica*] |
| MD10G1179100 | RecName: Full=Polygalacturonase; Short=PG; AltName: Full=Pectinase; Flags: Precursor |
| MD10G1179300 | PREDICTED: uncharacterized protein LOC103445599 [*Malus* *domestica*] |
| MD10G1179400 | PREDICTED: uncharacterized protein LOC103412245 [*Malus* *domestica*] |
| MD10G1187200 | PREDICTED: LOW QUALITY PROTEIN: uncharacterized protein LOC108174283 [*Malus* *domestica*] |
| MD10G1192000 | PREDICTED: probable galacturonosyltransferase 6 isoform X1 [*Malus* *domestica*] |
| MD10G1192900 | PREDICTED: auxin-induced protein AUX28 [*Malus* *domestica*] |
| MD10G1194700 | PREDICTED: uncharacterized endoplasmic reticulum membrane protein C16E8.02-like [*Malus* *domestica*] |
| MD10G1199900 | PREDICTED: decapping nuclease DXO homolog, chloroplastic-like isoform X3 [*Malus* *domestica*] |
| MD10G1202100 | PREDICTED: auxin-responsive protein SAUR71 [*Malus* *domestica*] |
| MD10G1208900 | SKP1-like protein 11 [*Malus* *domestica*] |
| MD10G1210800 | PREDICTED: uncharacterized protein LOC103445882 [*Malus* *domestica*] |
| MD10G1217000 | PREDICTED: organic cation/carnitine transporter 3-like isoform X1 [*Pyrus* x *bretschneideri*] |
| MD10G1218100 | PREDICTED: metacaspase-4-like [*Malus* *domestica*] |
| MD10G1218500 | PREDICTED: uncharacterized protein LOC103445940 [*Malus* *domestica*] |
| MD10G1219500 | PREDICTED: LOW QUALITY PROTEIN: isocitrate dehydrogenase [NADP]-like [*Malus* *domestica*] |
| MD10G1219900 | PREDICTED: ABC transporter D family member 2, chloroplastic-like [*Malus* *domestica*] |
| MD10G1221800 | -- |
| MD10G1222500 | PREDICTED: receptor-like protein 12 [*Malus* *domestica*] |
| MD10G1224100 | PREDICTED: plasma membrane-associated cation-binding protein 1 [*Malus* *domestica*] |
| MD10G1241100 | PREDICTED: probable lysine-specific demethylase JMJ14 [*Malus* *domestica*] |
| MD10G1243000 | PREDICTED: probable WRKY transcription factor 14 [*Malus* *domestica*] |
| MD10G1248700 | PREDICTED: coatomer subunit alpha-1-like [*Malus* *domestica*] |
| MD10G1268700 | PREDICTED: eukaryotic translation initiation factor 4E-1-like [*Pyrus* x *bretschneideri*] |
| MD10G1270500 | PREDICTED: uncharacterized protein LOC103429666 [*Malus* *domestica*] |
| MD10G1272000 | PREDICTED: MACPF domain-containing protein CAD1-like [*Malus* *domestica*] |
| MD10G1277000 | PREDICTED: uncharacterized protein LOC103429684 [*Malus* *domestica*] |
| MD10G1278100 | -- |
| MD10G1283600 | PREDICTED: uncharacterized protein LOC103434862 [*Malus* *domestica*] |
| MD10G1284100 | PREDICTED: nudix hydrolase 15, mitochondrial-like [*Malus* *domestica*] |
| MD10G1288500 | LRR receptor-like protein kinase m4 [*Malus* *domestica*] |
| MD10G1298100 | -- |
| MD10G1298200 | PREDICTED: polyphenol oxidase, chloroplastic-like [*Malus* *domestica*] |
| MD10G1300700 | PREDICTED: uncharacterized protein LOC103446456 [*Malus* *domestica*] |
| MD10G1301000 | PREDICTED: putative glycine-rich cell wall structural protein 1 [*Malus* *domestica*] |
| MD10G1304000 | PREDICTED: sugar carrier protein C [*Pyrus* x *bretschneideri*] |
| MD10G1309100 | PREDICTED: NAD(P)H dehydrogenase (quinone) FQR1-like [*Malus* *domestica*] |
| MD10G1311000 | (E,E)-alpha-farnesene synthase [*Malus* *domestica*] |
| MD10G1312900 | PREDICTED: cysteine-rich receptor-like protein kinase 10 [*Malus* *domestica*] |
| MD10G1319100 | PREDICTED: uncharacterized protein LOC103446863 [*Malus* *domestica*] |
| MD10G1319500 | PREDICTED: zinc finger MYND domain-containing protein 15 [*Malus* *domestica*] |
| MD10G1320100 | PREDICTED: SWI/SNF complex subunit SWI3B-like [*Malus* *domestica*] |
| MD10G1320600 | PREDICTED: uncharacterized protein LOC103412541 [*Malus* *domestica*] |
| MD10G1322500 | PREDICTED: putative lactoylglutathione lyase [*Pyrus* x *bretschneideri*] |
| MD10G1327200 | PREDICTED: uncharacterized protein LOC103446750 [*Malus* *domestica*] |
| MD10G1328000 | PREDICTED: death-associated inhibitor of apoptosis 1-like [*Malus* *domestica*] |
| MD10G1328100 | RecName: Full=1-aminocyclopropane-1-carboxylate oxidase 1 |
| MD10G1329100 | PREDICTED: tropinone reductase-like 3 [*Malus* *domestica*] |
| MD10G1332700 | PREDICTED: tonoplast dicarboxylate transporter-like [*Pyrus* x *bretschneideri*] |
| MD10G1337500 | PREDICTED: pollen-specific leucine-rich repeat extensin-like protein 4 isoform X1 [*Malus* *domestica*] |
| MD10G1337700 | PREDICTED: uncharacterized protein LOC103446809 [*Malus* *domestica*] |
| MD10G1338200 | PREDICTED: putative serine/threonine-protein kinase [*Pyrus* x *bretschneideri*] |
| MD11G1003700 | -- |
| MD11G1014500 | PREDICTED: protein EMBRYO SAC DEVELOPMENT ARREST 3, chloroplastic-like [*Pyrus* x *bretschneideri*] |
| MD11G1020600 | PREDICTED: accelerated cell death 11-like [*Pyrus* x *bretschneideri*] |
| MD11G1021300 | PREDICTED: G-type lectin S-receptor-like serine/threonine-protein kinase LECRK4 [*Malus* *domestica*] |
| MD11G1027500 | PREDICTED: beta-glucosidase 12-like [*Malus* *domestica*] |
| MD11G1028100 | PREDICTED: transcription factor bHLH130-like isoform X2 [*Malus* *domestica*] |
| MD11G1039500 | PREDICTED: BTB/POZ domain-containing protein At1g55760-like [*Malus* *domestica*] |
| MD11G1041800 | PREDICTED: probable amino acid permease 7 [*Pyrus* x *bretschneideri*] |
| MD11G1048400 | PREDICTED: calcium uniporter protein 6, mitochondrial-like [*Malus* *domestica*] |
| MD11G1049000 | -- |
| MD11G1049800 | PREDICTED: ABC transporter B family member 25, mitochondrial-like [*Pyrus* x *bretschneideri*] |
| MD11G1054100 | PREDICTED: non-specific lipid-transfer protein 1-like [*Malus* *domestica*] |
| MD11G1054900 | PREDICTED: uncharacterized protein LOC103963293 [*Pyrus* x *bretschneideri*] |
| MD11G1059900 | PREDICTED: isoleucine N-monooxygenase 1-like [*Malus* *domestica*] |
| MD11G1063500 | PREDICTED: glucan endo-1,3-beta-glucosidase 9-like [*Malus* *domestica*] |
| MD11G1081300 | PREDICTED: uncharacterized protein LOC103436659 [*Malus* *domestica*] |
| MD11G1090100 | PREDICTED: probable disease resistance protein At4g27220 [*Malus* *domestica*] |
| MD11G1093700 | PREDICTED: tropinone reductase homolog At2g29330-like isoform X1 [*Malus* *domestica*] |
| MD11G1093900 | PREDICTED: tropinone reductase homolog At1g07440-like isoform X2 [*Pyrus* x *bretschneideri*] |
| MD11G1097500 | PREDICTED: uncharacterized protein LOC103964682 [*Pyrus* x *bretschneideri*] |
| MD11G1097600 | PREDICTED: uncharacterized protein LOC103412863 [*Malus* *domestica*] |
| MD11G1098000 | PREDICTED: scarecrow-like protein 30 [*Malus* *domestica*] |
| MD11G1110300 | PREDICTED: actin [*Malus* *domestica*] |
| MD11G1110600 | PREDICTED: putative E3 ubiquitin-protein ligase XBAT31 [*Malus* *domestica*] |
| MD11G1111600 | PREDICTED: uncharacterized protein LOC103447724 [*Malus* *domestica*] |
| MD11G1114400 | PREDICTED: uncharacterized protein LOC103948945 isoform X1 [*Pyrus* x *bretschneideri*] |
| MD11G1116300 | PREDICTED: exocyst complex component EXO70B1-like [*Malus* *domestica*] |
| MD11G1116600 | PREDICTED: nudix hydrolase 9 [*Pyrus* x *bretschneideri*] |
| MD11G1126100 | PREDICTED: uncharacterized protein LOC103413046 [*Malus* *domestica*] |
| MD11G1132100 | PREDICTED: DEAD-box ATP-dependent RNA helicase 42 [*Malus* *domestica*] |
| MD11G1134600 | PREDICTED: probable E3 ubiquitin-protein ligase LUL2 [*Malus* *domestica*] |
| MD11G1138900 | PREDICTED: cytokinin riboside 5&apos;-monophosphate phosphoribohydrolase LOG1 [*Malus* *domestica*] |
| MD11G1139100 | PREDICTED: putative cyclic nucleotide-gated ion channel 15 [*Malus* *domestica*] |
| MD11G1142300 | PREDICTED: uncharacterized protein LOC103964818 [*Pyrus* x *bretschneideri*] |
| MD11G1146900 | PREDICTED: protein NUCLEAR FUSION DEFECTIVE 4 [*Malus* *domestica*] |
| MD11G1152500 | PREDICTED: aspartic proteinase-like protein 2 [*Malus* *domestica*] |
| MD11G1159700 | PREDICTED: putative low molecular weight protein-tyrosine-phosphatase slr0328 [*Malus* *domestica*] |
| MD11G1160600 | PREDICTED: heme-binding protein 2-like [*Malus* *domestica*] |
| MD11G1161800 | PREDICTED: fatty acyl-CoA reductase 3-like [*Pyrus* x *bretschneideri*] |
| MD11G1164500 | PREDICTED: probable calcium-binding protein CML48 [*Malus* *domestica*] |
| MD11G1171500 | PREDICTED: protein DMR6-LIKE OXYGENASE 2-like [*Malus* *domestica*] |
| MD11G1172400 | PREDICTED: probable CCR4-associated factor 1 homolog 9 [*Pyrus* x *bretschneideri*] |
| MD11G1172500 | PREDICTED: transcription repressor OFP4-like [*Pyrus* x *bretschneideri*] |
| MD11G1178000 | PREDICTED: beta-glucosidase BoGH3B-like [*Malus* *domestica*] |
| MD11G1178400 | PREDICTED: indole-3-acetaldehyde oxidase-like isoform X2 [*Pyrus* x *bretschneideri*] |
| MD11G1179700 | PREDICTED: non-specific lipid transfer protein GPI-anchored 2-like isoform X1 [*Malus* *domestica*] |
| MD11G1189000 | PREDICTED: glucan endo-1,3-beta-glucosidase-like [*Malus* *domestica*] |
| MD11G1189900 | PREDICTED: ABC transporter G family member 15-like [*Malus* *domestica*] |
| MD11G1190800 | cinnamoyl-CoA reductase 1-like [*Malus* *domestica*] |
| MD11G1192000 | PREDICTED: sulfite reductase [ferredoxin], chloroplastic [*Malus* *domestica*] |
| MD11G1204300 | -- |
| MD11G1213600 | PREDICTED: aspartic proteinase A1-like [*Malus* *domestica*] |
| MD11G1217400 | PREDICTED: protein translation factor SUI1 homolog [*Malus* *domestica*] |
| MD11G1219200 | PREDICTED: uncharacterized protein LOC103448408 [*Malus* *domestica*] |
| MD11G1220000 | -- |
| MD11G1221600 | PREDICTED: uncharacterized protein LOC103967061 [*Pyrus* x *bretschneideri*] |
| MD11G1223700 | PREDICTED: glucose-6-phosphate/phosphate translocator 2, chloroplastic [*Malus* *domestica*] |
| MD11G1224800 | PREDICTED: thiamine thiazole synthase, chloroplastic-like [*Pyrus* x *bretschneideri*] |
| MD11G1229100 | PREDICTED: dihydroflavonol-4-reductase-like [*Malus* *domestica*] |
| MD11G1229700 | PREDICTED: organic cation/carnitine transporter 4-like [*Malus* *domestica*] |
| MD11G1231700 | PREDICTED: FAD-dependent urate hydroxylase-like [*Malus* *domestica*] |
| MD11G1235500 | PREDICTED: uncharacterized protein LOC103448604 [*Malus* *domestica*] |
| MD11G1238000 | PREDICTED: uncharacterized protein LOC103433940 [*Malus* *domestica*] |
| MD11G1239700 | PREDICTED: protein SYM1-like isoform X1 [*Malus* *domestica*] |
| MD11G1247600 | PREDICTED: beta-glucuronosyltransferase GlcAT14B-like [*Malus* *domestica*] |
| MD11G1251600 | PREDICTED: 3-hydroxybutyryl-CoA dehydrogenase-like [*Malus* *domestica*] |
| MD11G1252200 | PREDICTED: transcription factor EMB1444 [*Malus* *domestica*] |
| MD11G1255900 | PREDICTED: uncharacterized exonuclease domain-containing protein At3g15140 [*Malus* *domestica*] |
| MD11G1257300 | PREDICTED: transcription elongation factor 1 homolog [*Malus* *domestica*] |
| MD11G1272000 | PREDICTED: protein SRG1-like [*Pyrus* x *bretschneideri*] |
| MD11G1277200 | PREDICTED: guanine nucleotide-binding protein subunit gamma 1-like [*Malus* *domestica*] |
| MD11G1278500 | PREDICTED: protein SPIRRIG [*Pyrus* x *bretschneideri*] |
| MD11G1285000 | PREDICTED: cyclin-P3-1 [*Malus* *domestica*] |
| MD11G1290400 | PREDICTED: aspartic proteinase-like protein 2 [*Pyrus* x *bretschneideri*] |
| MD11G1291700 | PREDICTED: uncharacterized protein LOC103449078 [*Malus* *domestica*] |
| MD11G1293000 | PREDICTED: probable indole-3-acetic acid-amido synthetase GH3.6 [*Malus* *domestica*] |
| MD11G1293600 | PREDICTED: receptor-like cytosolic serine/threonine-protein kinase RBK2 [*Malus* *domestica*] |
| MD11G1293900 | histidine-containing phosphotransfer 3a [*Malus* *domestica*] |
| MD11G1294100 | PREDICTED: LEC14B protein-like isoform X1 [*Malus* *domestica*] |
| MD11G1300800 | PREDICTED: uncharacterized protein LOC103967389 [*Pyrus* x *bretschneideri*] |
| MD11G1305000 | PREDICTED: stress-related protein-like [*Malus* *domestica*] |
| MD11G1307200 | PREDICTED: pectinesterase-like [*Malus* *domestica*] |
| MD11G1307500 | PREDICTED: probable pectinesterase/pectinesterase inhibitor 7 [*Malus* *domestica*] |
| MD11G1308100 | PREDICTED: uncharacterized protein At5g01610-like [*Malus* *domestica*] |
| MD11G1308400 | PREDICTED: protein SENESCENCE-ASSOCIATED GENE 21, mitochondrial-like [*Malus* *domestica*] |
| MD11G1311200 | PREDICTED: uncharacterized protein LOC103413369 [*Malus* *domestica*] |
| MD11G1312100 | PREDICTED: uncharacterized protein LOC103413369 [*Malus* *domestica*] |
| MD11G1313800 | PREDICTED: ABC transporter C family member 14-like [*Pyrus* x *bretschneideri*] |
| MD11G1315500 | PREDICTED: uncharacterized protein LOC103449217 [*Malus* *domestica*] |
| MD11G1317200 | PREDICTED: uncharacterized protein LOC103422135 [*Malus* *domestica*] |
| MD12G1003700 | PREDICTED: uncharacterized protein LOC103956213 [*Pyrus* x *bretschneideri*] |
| MD12G1004700 | PREDICTED: tubulin alpha-3 chain-like [*Malus* *domestica*] |
| MD12G1011500 | PREDICTED: cation/calcium exchanger 1 [*Malus* *domestica*] |
| MD12G1011900 | PREDICTED: uncharacterized protein LOC103449355 [*Malus* *domestica*] |
| MD12G1013800 | PREDICTED: probable S-sulfocysteine synthase, chloroplastic isoform X2 [*Malus* *domestica*] |
| MD12G1018200 | hypothetical protein PRUPE_7G116900 [*Prunus persica*] |
| MD12G1018600 | PREDICTED: uncharacterized protein LOC103449449 [*Malus* *domestica*] |
| MD12G1034200 | PREDICTED: transcription repressor OFP13-like [*Malus* *domestica*] |
| MD12G1038200 | PREDICTED: uncharacterized protein LOC108866853 [*Pyrus* x *bretschneideri*] |
| MD12G1038300 | PREDICTED: benzyl alcohol O-benzoyltransferase-like [*Malus* *domestica*] |
| MD12G1038600 | PREDICTED: NDR1/HIN1-Like protein 3-like [*Malus* *domestica*] |
| MD12G1038700 | PREDICTED: NDR1/HIN1-like protein 12 [*Malus* *domestica*] |
| MD12G1038800 | PREDICTED: NDR1/HIN1-like protein 12 [*Malus* *domestica*] |
| MD12G1040900 | PREDICTED: E3 ubiquitin-protein ligase PUB23-like [*Malus* *domestica*] |
| MD12G1047000 | PREDICTED: amino acid transporter ANT1 [*Malus* *domestica*] |
| MD12G1048500 | PREDICTED: ubiquitin carboxyl-terminal hydrolase 13-like isoform X3 [*Pyrus* x *bretschneideri*] |
| MD12G1063100 | PREDICTED: uncharacterized protein LOC103939821 [*Pyrus* x *bretschneideri*] |
| MD12G1066900 | PREDICTED: uncharacterized protein LOC103424341 [*Malus* *domestica*] |
| MD12G1067900 | hypothetical protein PRUPE_7G066300 [*Prunus persica*] |
| MD12G1081200 | -- |
| MD12G1081300 | PREDICTED: DNA-directed RNA polymerase V subunit 5A-like isoform X1 [*Malus* *domestica*] |
| MD12G1094200 | PREDICTED: pyrroline-5-carboxylate reductase-like [*Pyrus* x *bretschneideri*] |
| MD12G1095300 | PREDICTED: transcription factor bHLH106-like [*Malus* *domestica*] |
| MD12G1097200 | PREDICTED: LOW QUALITY PROTEIN: uncharacterized protein LOC103927674 [*Pyrus* x *bretschneideri*] |
| MD12G1097400 | PREDICTED: auxin-induced protein 15A-like [*Malus* *domestica*] |
| MD12G1102800 | PREDICTED: dnaJ homolog subfamily C member 28-like [*Malus* *domestica*] |
| MD12G1103200 | PREDICTED: purine-uracil permease NCS1-like [*Malus* *domestica*] |
| MD12G1109600 | PREDICTED: uncharacterized protein LOC103412688 [*Malus* *domestica*] |
| MD12G1113000 | PREDICTED: heavy metal-associated isoprenylated plant protein 3-like [*Malus* *domestica*] |
| MD12G1113200 | PREDICTED: exonuclease V, chloroplastic [*Malus* *domestica*] |
| MD12G1113400 | PREDICTED: uncharacterized protein LOC103450013 [*Malus* *domestica*] |
| MD12G1126400 | PREDICTED: LOW QUALITY PROTEIN: zinc finger CCCH domain-containing protein 11-like [*Malus* *domestica*] |
| MD12G1127300 | PREDICTED: putative disease resistance protein RGA1 isoform X2 [*Pyrus* x *bretschneideri*] |
| MD12G1129400 | PREDICTED: glutathione S-transferase L3-like [*Malus* *domestica*] |
| MD12G1135600 | PREDICTED: uncharacterized protein LOC103436148 isoform X3 [*Malus* *domestica*] |
| MD12G1143700 | PREDICTED: uncharacterized protein At5g02240-like [*Malus* *domestica*] |
| MD12G1145500 | PREDICTED: high-light-induced protein, chloroplastic [*Malus* *domestica*] |
| MD12G1149800 | PREDICTED: aldo-keto reductase family 4 member C10-like [*Pyrus* x *bretschneideri*] |
| MD12G1155300 | PREDICTED: universal stress protein PHOS32-like [*Malus* *domestica*] |
| MD12G1158300 | PREDICTED: protein transport protein yos1-like [*Malus* *domestica*] |
| MD12G1158500 | PREDICTED: uncharacterized protein At1g04910-like [*Pyrus* x *bretschneideri*] |
| MD12G1161200 | PREDICTED: uncharacterized protein LOC103443768 [*Malus* *domestica*] |
| MD12G1161300 | PREDICTED: uncharacterized acetyltransferase At3g50280-like [*Malus* *domestica*] |
| MD12G1168300 | PREDICTED: trihelix transcription factor GT-3b [*Malus* *domestica*] |
| MD12G1170000 | PREDICTED: transcription factor TGA5-like [*Malus* *domestica*] |
| MD12G1178200 | PREDICTED: ferritin-4, chloroplastic-like [*Malus* *domestica*] |
| MD12G1178300 | PREDICTED: uncharacterized PE-PGRS family protein PE_PGRS20-like isoform X1 [*Malus* *domestica*] |
| MD12G1180900 | PREDICTED: CASP-like protein 4B1 [*Malus* *domestica*] |
| MD12G1182900 | protein LURP-one-related 10-like [*Prunus avium*] |
| MD12G1183000 | PREDICTED: protein LURP-one-related 15-like [*Malus* *domestica*] |
| MD12G1183300 | PREDICTED: uncharacterized protein LOC108172847 [*Malus* *domestica*] |
| MD12G1187000 | PREDICTED: non-specific lipid-transfer protein 1-like [*Malus* *domestica*] |
| MD12G1187100 | non-specific lipid-transfer protein precursor [*Malus* *domestica*] |
| MD12G1188800 | PREDICTED: vacuolar-sorting receptor 1-like [*Malus* *domestica*] |
| MD12G1190400 | PREDICTED: serine/threonine-protein kinase AtPK2/AtPK19-like [*Malus* *domestica*] |
| MD12G1194400 | PREDICTED: uncharacterized protein LOC103451252 [*Malus* *domestica*] |
| MD12G1195800 | PREDICTED: serine/threonine-protein kinase At5g01020-like [*Malus* *domestica*] |
| MD12G1197000 | PREDICTED: uncharacterized protein LOC108172041 [*Malus* *domestica*] |
| MD12G1197100 | PREDICTED: loricrin isoform X2 [*Pyrus* x *bretschneideri*] |
| MD12G1197400 | PREDICTED: germin-like protein subfamily 2 member 1 [*Prunus mume*] |
| MD12G1199100 | PREDICTED: hydrophobic protein RCI2B-like [*Malus* *domestica*] |
| MD12G1199300 | PREDICTED: hydrophobic protein RCI2A-like [*Malus* *domestica*] |
| MD12G1203900 | -- |
| MD12G1205900 | nicotianamine synthase [*Malus* baccata var. xiaojinensis] |
| MD12G1206800 | PREDICTED: cyclic phosphodiesterase-like [*Malus* *domestica*] |
| MD12G1209000 | PREDICTED: exonuclease DPD1, chloroplastic/mitochondrial [*Malus* *domestica*] |
| MD12G1217800 | PREDICTED: uncharacterized protein LOC103450934 [*Malus* *domestica*] |
| MD12G1219200 | PREDICTED: uncharacterized protein LOC103344636 [*Prunus mume*] |
| MD12G1221800 | PREDICTED: nuclear transcription factor Y subunit C-3-like [*Malus* *domestica*] |
| MD12G1226000 | PREDICTED: uncharacterized protein LOC103959551 isoform X1 [*Pyrus* x *bretschneideri*] |
| MD12G1227000 | PREDICTED: uncharacterized protein LOC103423569 isoform X1 [*Malus* *domestica*] |
| MD12G1227900 | PREDICTED: protein BONZAI 3-like [*Malus* *domestica*] |
| MD12G1229400 | PREDICTED: monothiol glutaredoxin-S17-like [*Pyrus* x *bretschneideri*] |
| MD12G1231000 | PREDICTED: acyl carrier protein 1, chloroplastic-like [*Pyrus* x *bretschneideri*] |
| MD12G1232100 | PREDICTED: cysteine proteinase inhibitor 5-like [*Pyrus* x *bretschneideri*] |
| MD12G1235100 | PREDICTED: pre-rRNA-processing protein TSR2-like [*Malus* *domestica*] |
| MD12G1238500 | PREDICTED: uncharacterized protein LOC103451019 [*Malus* *domestica*] |
| MD12G1238900 | PREDICTED: protein ECERIFERUM 26-like [*Malus* *domestica*] |
| MD12G1239000 | PREDICTED: perakine reductase-like [*Malus* *domestica*] |
| MD12G1246500 | cell division cycle 5-like protein isoform X1 [*Prunus avium*] |
| MD12G1248800 | TIR-NBS-LRR disease resistance protein [*Malus* *domestica*] |
| MD12G1256400 | PREDICTED: receptor-like protein kinase FERONIA [*Malus* *domestica*] |
| MD12G1257000 | PREDICTED: protein LUTEIN DEFICIENT 5, chloroplastic isoform X1 [*Pyrus* x *bretschneideri*] |
| MD12G1258400 | PREDICTED: uncharacterized protein LOC103451132 [*Malus* *domestica*] |
| MD12G1258800 | PREDICTED: monothiol glutaredoxin-S7, chloroplastic-like [*Malus* *domestica*] |
| MD13G1016000 | PREDICTED: purple acid phosphatase 3-like [*Malus* *domestica*] |
| MD13G1016100 | purple acid phosphatase 8 [*Prunus persica*] |
| MD13G1016500 | PREDICTED: LOW QUALITY PROTEIN: probable xyloglucan endotransglucosylase/hydrolase protein 28 [*Malus* *domestica*] |
| MD13G1025200 | PREDICTED: homeobox-leucine zipper protein HOX3-like [*Malus* *domestica*] |
| MD13G1026800 | PREDICTED: heavy metal-associated isoprenylated plant protein 20-like [*Malus* *domestica*] |
| MD13G1027400 | PREDICTED: uncharacterized protein LOC103430629 [*Malus* *domestica*] |
| MD13G1027500 | PREDICTED: uncharacterized protein LOC103430628 [*Malus* *domestica*] |
| MD13G1027900 | -- |
| MD13G1028000 | PREDICTED: probable ribose-5-phosphate isomerase 2 [*Malus* *domestica*] |
| MD13G1028100 | PREDICTED: uncharacterized protein LOC103451599, partial [*Malus* *domestica*] |
| MD13G1028300 | PREDICTED: uncharacterized protein LOC103414227 [*Malus* *domestica*] |
| MD13G1030400 | PREDICTED: dynein light chain 1, cytoplasmic-like [*Malus* *domestica*] |
| MD13G1033800 | PREDICTED: uncharacterized protein LOC103414234 [*Malus* *domestica*] |
| MD13G1034500 | PREDICTED: uncharacterized protein LOC103402651 [*Malus* *domestica*] |
| MD13G1039200 | PREDICTED: zinc finger protein 6-like [*Malus* *domestica*] |
| MD13G1039900 | PREDICTED: myb-related protein 305 [*Malus* *domestica*] |
| MD13G1042100 | PREDICTED: uncharacterized protein LOC103451652 [*Malus* *domestica*] |
| MD13G1042400 | PREDICTED: 3-ketoacyl-CoA synthase 6-like [*Malus* *domestica*] |
| MD13G1050400 | PREDICTED: putative phospholipid-transporting ATPase 9 [*Malus* *domestica*] |
| MD13G1052700 | PREDICTED: LOW QUALITY PROTEIN: protein NSP-INTERACTING KINASE 2-like [*Malus* *domestica*] |
| MD13G1053600 | PREDICTED: ruBisCO large subunit-binding protein subunit beta, chloroplastic isoform X1 [*Malus* *domestica*] |
| MD13G1056800 | PREDICTED: ACT domain-containing protein ACR4-like [*Malus* *domestica*] |
| MD13G1057800 | PREDICTED: cytochrome P450 CYP749A22-like isoform X1 [*Malus* *domestica*] |
| MD13G1061300 | PREDICTED: ubiquinone biosynthesis protein COQ4 homolog, mitochondrial [*Malus* *domestica*] |
| MD13G1063100 | PREDICTED: erlin-2-B-like [*Malus* *domestica*] |
| MD13G1069200 | NAC transcription factor 29-like [*Malus* *domestica*] |
| MD13G1069400 | PREDICTED: uncharacterized endoplasmic reticulum membrane protein C16E8.02-like [*Malus* *domestica*] |
| MD13G1074100 | PREDICTED: F-box/kelch-repeat protein SKIP11 [*Malus* *domestica*] |
| MD13G1074200 | PREDICTED: peptidyl-prolyl cis-trans isomerase CYP23-like [*Malus* *domestica*] |
| MD13G1075900 | PREDICTED: uncharacterized protein LOC103423804 [*Malus* *domestica*] |
| MD13G1078700 | PREDICTED: mitochondrial outer membrane protein porin of 36 kDa-like [*Malus* *domestica*] |
| MD13G1082000 | PREDICTED: glutathione S-transferase U17-like [*Malus* *domestica*] |
| MD13G1084400 | PREDICTED: glycosyltransferase family 92 protein RCOM_0530710-like [*Malus* *domestica*] |
| MD13G1087400 | PREDICTED: uncharacterized protein LOC103451926 [*Malus* *domestica*] |
| MD13G1090300 | carotenoid cleavage dioxygenase 4 [*Malus* *domestica*] |
| MD13G1101200 | PREDICTED: non-functional NADPH-dependent codeinone reductase 2-like [*Pyrus* x *bretschneideri*] |
| MD13G1105300 | PREDICTED: uncharacterized WD repeat-containing protein alr3466 [*Malus* *domestica*] |
| MD13G1106800 | PREDICTED: uncharacterized protein LOC103423882, partial [*Malus* *domestica*] |
| MD13G1112000 | PREDICTED: 3-oxoacyl-[acyl-carrier-protein] reductase 4 [*Malus* *domestica*] |
| MD13G1122100 | PREDICTED: probable WRKY transcription factor 75 [*Malus* *domestica*] |
| MD13G1122500 | PREDICTED: uncharacterized protein LOC103452426 [*Malus* *domestica*] |
| MD13G1124900 | PREDICTED: NAC domain-containing protein 83-like [*Pyrus* x *bretschneideri*] |
| MD13G1128200 | hypothetical protein PRUPE_1G217800 [*Prunus persica*] |
| MD13G1130700 | ethylene-responsive transcription factor ERF113-like [*Malus* *domestica*] |
| MD13G1134300 | PREDICTED: uncharacterized protein LOC103944736 [*Pyrus* x *bretschneideri*] |
| MD13G1141200 | PREDICTED: pto-interacting protein 1 [*Malus* *domestica*] |
| MD13G1144600 | PREDICTED: solute carrier family 25 member 44-like isoform X1 [*Malus* *domestica*] |
| MD13G1147600 | PREDICTED: stress-response A/B barrel domain-containing protein HS1-like [*Malus* *domestica*] |
| MD13G1158300 | PREDICTED: uncharacterized protein LOC103405753 [*Malus* *domestica*] |
| MD13G1159600 | PREDICTED: COBRA-like protein 7 [*Malus* *domestica*] |
| MD13G1162000 | PREDICTED: probable polygalacturonase [*Malus* *domestica*] |
| MD13G1164300 | PREDICTED: probable acyl-CoA dehydrogenase IBR3 [*Malus* *domestica*] |
| MD13G1182400 | PREDICTED: uncharacterized protein At5g19025-like [*Malus* *domestica*] |
| MD13G1188500 | PREDICTED: probable membrane-associated kinase regulator 1 [*Pyrus* x *bretschneideri*] |
| MD13G1191400 | PREDICTED: uncharacterized protein LOC103935975 [*Pyrus* x *bretschneideri*] |
| MD13G1198400 | PREDICTED: uncharacterized protein LOC103453160 isoform X1 [*Malus* *domestica*] |
| MD13G1198700 | PREDICTED: BI1-like protein [*Malus* *domestica*] |
| MD13G1199600 | PREDICTED: 1,2-dihydroxy-3-keto-5-methylthiopentene dioxygenase 2 [*Malus* *domestica*] |
| MD13G1201300 | -- |
| MD13G1203100 | PREDICTED: 3-ketoacyl-CoA synthase 20-like isoform X1 [*Malus* *domestica*] |
| MD13G1205200 | PREDICTED: magnesium transporter MRS2-I-like [*Malus* *domestica*] |
| MD13G1205600 | PREDICTED: PRA1 family protein D-like [*Malus* *domestica*] |
| MD13G1208700 | hypothetical protein PRUPE_1G032700 [*Prunus persica*] |
| MD13G1210400 | PREDICTED: enoyl-CoA delta isomerase 2, peroxisomal-like [*Pyrus* x *bretschneideri*] |
| MD13G1211100 | PREDICTED: 1-aminocyclopropane-1-carboxylate oxidase homolog 3-like [*Malus* *domestica*] |
| MD13G1213100 | ethylene-responsive transcription factor 1B-like [*Malus* *domestica*] |
| MD13G1217400 | peroxisomal membrane protein PMP22 [Hevea brasiliensis] |
| MD13G1220400 | PREDICTED: uncharacterized protein LOC103927056 [*Pyrus* x *bretschneideri*] |
| MD13G1222900 | PREDICTED: 3-isopropylmalate dehydratase large subunit-like [*Pyrus* x *bretschneideri*] |
| MD13G1231400 | PREDICTED: protein SULFUR DEFICIENCY-INDUCED 2 [*Malus* *domestica*] |
| MD13G1237300 | probable xyloglucan endotransglucosylase/hydrolase protein 23 precursor [*Malus* *domestica*] |
| MD13G1240100 | PREDICTED: mannosyl-oligosaccharide 1,2-alpha-mannosidase MNS1-like isoform X2 [*Malus* *domestica*] |
| MD13G1241900 | PREDICTED: uncharacterized protein LOC103453776 [*Malus* *domestica*] |
| MD13G1246700 | PREDICTED: peroxidase 16-like [*Malus* *domestica*] |
| MD13G1249200 | PREDICTED: pentatricopeptide repeat-containing protein At2g18940, chloroplastic-like [*Malus* *domestica*] |
| MD13G1257600 | PREDICTED: uncharacterized protein LOC103454680 [*Malus* *domestica*] |
| MD13G1267500 | PREDICTED: flap endonuclease GEN-like 1 [*Pyrus* x *bretschneideri*] |
| MD13G1274100 | PREDICTED: cytochrome c oxidase subunit 6b-2 [*Pyrus* x *bretschneideri*] |
| MD13G1277900 | PREDICTED: protein fluG [*Malus* *domestica*] |
| MD14G1001000 | NAC transcription factor 29 [*Malus* *domestica*] |
| MD14G1002100 | PREDICTED: short-chain type dehydrogenase/reductase-like [*Malus* *domestica*] |
| MD14G1009500 | PREDICTED: uncharacterized protein LOC103935003 [*Pyrus* x *bretschneideri*] |
| MD14G1010100 | hypothetical protein PRUPE_7G016200 [*Prunus persica*] |
| MD14G1010200 | PREDICTED: uncharacterized protein LOC103944347 [*Pyrus* x *bretschneideri*] |
| MD14G1011700 | PREDICTED: BAHD acyltransferase At5g47980-like [*Pyrus* x *bretschneideri*] |
| MD14G1016200 | hypothetical protein PRUPE_7G116900 [*Prunus persica*] |
| MD14G1020100 | PREDICTED: solute carrier family 35 member F1-like [*Pyrus* x *bretschneideri*] |
| MD14G1021400 | PREDICTED: uncharacterized protein LOC103956408 [*Pyrus* x *bretschneideri*] |
| MD14G1022300 | PREDICTED: iron-sulfur assembly protein IscA-like 1, mitochondrial [*Malus* *domestica*] |
| MD14G1037500 | PREDICTED: protein YLS9-like [*Malus* *domestica*] |
| MD14G1040300 | PREDICTED: E3 ubiquitin-protein ligase PUB23-like [*Pyrus* x *bretschneideri*] |
| MD14G1041300 | PREDICTED: nuclear transcription factor Y subunit A-10-like [*Malus* *domestica*] |
| MD14G1049400 | PREDICTED: uncharacterized protein LOC103453763 [*Malus* *domestica*] |
| MD14G1052500 | PREDICTED: dirigent protein 22-like [*Pyrus* x *bretschneideri*] |
| MD14G1055500 | -- |
| MD14G1056200 | PREDICTED: homeobox-leucine zipper protein HOX11 [*Pyrus* x *bretschneideri*] |
| MD14G1057600 | PREDICTED: probable protein phosphatase 2C 68 [*Malus* *domestica*] |
| MD14G1058600 | PREDICTED: enoyl-[acyl-carrier-protein] reductase [NADH] 1, chloroplastic-like [*Malus* *domestica*] |
| MD14G1067300 | PREDICTED: 1-acyl-sn-glycerol-3-phosphate acyltransferase 2-like [*Pyrus* x *bretschneideri*] |
| MD14G1069300 | PREDICTED: guanylate kinase 2-like isoform X1 [*Pyrus* x *bretschneideri*] |
| MD14G1070300 | PREDICTED: probable galactinol--sucrose galactosyltransferase 2 [*Pyrus* x *bretschneideri*] |
| MD14G1071000 | PREDICTED: DNA-directed RNA polymerases II, IV and V subunit 12 [*Malus* *domestica*] |
| MD14G1087100 | -- |
| MD14G1090500 | PREDICTED: uncharacterized protein LOC103936977 [*Pyrus* x *bretschneideri*] |
| MD14G1101300 | PREDICTED: histone-lysine N-methyltransferase, H3 lysine-9 specific SUVH1-like [*Malus* *domestica*] |
| MD14G1105400 | PREDICTED: uncharacterized protein LOC103938466 [*Pyrus* x *bretschneideri*] |
| MD14G1111700 | PREDICTED: DET1- and DDB1-associated protein 1-like [*Pyrus* x *bretschneideri*] |
| MD14G1114500 | PREDICTED: late embryogenesis abundant protein At1g64065-like [*Malus* *domestica*] |
| MD14G1115400 | PREDICTED: uncharacterized protein LOC103424023 [*Malus* *domestica*] |
| MD14G1120000 | PREDICTED: ethylene-responsive transcription factor CRF2-like [*Pyrus* x *bretschneideri*] |
| MD14G1122100 | PREDICTED: uncharacterized protein LOC103415066 [*Malus* *domestica*] |
| MD14G1123300 | PREDICTED: KAT8 regulatory NSL complex subunit 3 [*Prunus mume*] |
| MD14G1128200 | PREDICTED: long chain acyl-CoA synthetase 4 [*Malus* *domestica*] |
| MD14G1129600 | PREDICTED: dynamin-related protein 5A [*Malus* *domestica*] |
| MD14G1134500 | PREDICTED: uncharacterized protein LOC108169620 [*Malus* *domestica*] |
| MD14G1137200 | PREDICTED: transcription factor MYC2-like [*Malus* *domestica*] |
| MD14G1137800 | PREDICTED: uncharacterized protein At3g28850-like [*Malus* *domestica*] |
| MD14G1138600 | PREDICTED: uncharacterized protein LOC108867497 [*Pyrus* x *bretschneideri*] |
| MD14G1141700 | PREDICTED: uncharacterized protein LOC103424423 [*Malus* *domestica*] |
| MD14G1143800 | PREDICTED: probable receptor-like protein kinase At1g33260 [*Malus* *domestica*] |
| MD14G1145500 | PREDICTED: uncharacterized protein LOC103962645 [*Pyrus* x *bretschneideri*] |
| MD14G1145800 | PREDICTED: succinate dehydrogenase assembly factor 2, mitochondrial-like [*Malus* *domestica*] |
| MD14G1149200 | PREDICTED: mitochondrial phosphate carrier protein 2, mitochondrial-like [*Malus* *domestica*] |
| MD14G1151300 | PREDICTED: bidirectional sugar transporter N3 [*Pyrus* x *bretschneideri*] |
| MD14G1152100 | PREDICTED: auxin-responsive protein SAUR71-like [*Pyrus* x *bretschneideri*] |
| MD14G1155400 | PREDICTED: gamma carbonic anhydrase-like 2, mitochondrial [*Malus* *domestica*] |
| MD14G1161000 | PREDICTED: mitochondrial pyruvate carrier 1 [*Malus* *domestica*] |
| MD14G1162500 | PREDICTED: uncharacterized protein LOC103455033 [*Malus* *domestica*] |
| MD14G1167000 | PREDICTED: BTB/POZ and TAZ domain-containing protein 1-like [*Pyrus* x *bretschneideri*] |
| MD14G1170100 | PREDICTED: quinolinate synthase, chloroplastic-like [*Malus* *domestica*] |
| MD14G1172100 | PREDICTED: uncharacterized protein LOC103455081 [*Malus* *domestica*] |
| MD14G1174200 | PREDICTED: disease resistance protein RPM1-like [*Malus* *domestica*] |
| MD14G1182200 | PREDICTED: CASP-like protein 4A3 [*Pyrus* x *bretschneideri*] |
| MD14G1183700 | PREDICTED: probable alpha,alpha-trehalose-phosphate synthase [UDP-forming] 9 [*Malus* *domestica*] |
| MD14G1187200 | PREDICTED: uncharacterized protein LOC103455261 [*Malus* *domestica*] |
| MD14G1188400 | PREDICTED: two-component response regulator ARR5-like [*Malus* *domestica*] |
| MD14G1197000 | PREDICTED: vacuolar protein sorting-associated protein 29 [*Malus* *domestica*] |
| MD14G1201000 | PREDICTED: malate dehydrogenase, chloroplastic-like [*Malus* *domestica*] |
| MD14G1201300 | PREDICTED: uncharacterized protein LOC103455356 [*Malus* *domestica*] |
| MD14G1202100 | PREDICTED: expansin-A1-like [*Malus* *domestica*] |
| MD14G1207600 | PREDICTED: protein DMR6-LIKE OXYGENASE 2-like [*Malus* *domestica*] |
| MD14G1209000 | PREDICTED: uncharacterized protein LOC103455400 [*Malus* *domestica*] |
| MD14G1214400 | PREDICTED: LOW QUALITY PROTEIN: cleavage stimulation factor subunit 50 [*Malus* *domestica*] |
| MD14G1221100 | PREDICTED: oleosin 1-like [*Malus* *domestica*] |
| MD14G1221600 | PREDICTED: transcription initiation factor TFIID subunit 2-like [*Malus* *domestica*] |
| MD14G1222200 | PREDICTED: transcription factor DIVARICATA-like [*Pyrus* x *bretschneideri*] |
| MD14G1222600 | PREDICTED: uncharacterized protein LOC103437980 [*Malus* *domestica*] |
| MD14G1227500 | PREDICTED: transcription factor MYB108-like [*Malus* *domestica*] |
| MD14G1228000 | PREDICTED: uncharacterized protein LOC103950681 [*Pyrus* x *bretschneideri*] |
| MD14G1228100 | PREDICTED: uncharacterized protein LOC103455509 [*Malus* *domestica*] |
| MD14G1230200 | PREDICTED: uncharacterized protein LOC103950620 [*Pyrus* x *bretschneideri*] |
| MD14G1232100 | PREDICTED: glutathione S-transferase U8-like [*Malus* *domestica*] |
| MD14G1234500 | PREDICTED: myb-related protein Zm38-like [*Malus* *domestica*] |
| MD14G1239300 | PREDICTED: dihydropyrimidine dehydrogenase (NADP(+)), chloroplastic-like [*Pyrus* x *bretschneideri*] |
| MD15G1005500 | PREDICTED: F-box protein SKIP23-like [*Malus* *domestica*] |
| MD15G1011300 | PREDICTED: uncharacterized protein LOC103430387 [*Malus* *domestica*] |
| MD15G1014200 | PREDICTED: auxin-induced protein 15A [*Malus* *domestica*] |
| MD15G1014300 | PREDICTED: auxin-responsive protein SAUR71-like [*Malus* *domestica*] |
| MD15G1020900 | PREDICTED: thioredoxin H1-like [*Malus* *domestica*] |
| MD15G1021000 | PREDICTED: thioredoxin H-type 2-like [*Malus* *domestica*] |
| MD15G1022100 | PREDICTED: GTPase HflX [*Malus* *domestica*] |
| MD15G1023800 | PREDICTED: 50S ribosomal protein L31, chloroplastic [*Malus* *domestica*] |
| MD15G1023900 | PREDICTED: glucose-1-phosphate adenylyltransferase large subunit, chloroplastic/amyloplastic-like [*Malus* *domestica*] |
| MD15G1028600 | PREDICTED: lysophospholipid acyltransferase LPEAT1-like [*Malus* *domestica*] |
| MD15G1032600 | PREDICTED: uncharacterized protein LOC103442641 [*Malus* *domestica*] |
| MD15G1033500 | PREDICTED: uncharacterized protein LOC103442641 [*Malus* *domestica*] |
| MD15G1033900 | PREDICTED: cytochrome P450 CYP82D47-like [*Malus* *domestica*] |
| MD15G1035900 | PREDICTED: mitogen-activated protein kinase kinase kinase YODA-like [*Malus* *domestica*] |
| MD15G1036300 | PREDICTED: aspartate aminotransferase P2, mitochondrial [*Malus* *domestica*] |
| MD15G1036500 | PREDICTED: ethylene-responsive transcription factor ERF003-like [*Malus* *domestica*] |
| MD15G1037200 | zinc finger protein 3-like [*Malus* *domestica*] |
| MD15G1041700 | -- |
| MD15G1043500 | PREDICTED: dynamin-related protein 3A-like [*Malus* *domestica*] |
| MD15G1044500 | hypothetical protein PRUPE_1G398400 [*Prunus persica*] |
| MD15G1057000 | PREDICTED: graves disease carrier protein homolog [*Malus* *domestica*] |
| MD15G1057900 | PREDICTED: probable ADP,ATP carrier protein At5g56450 [*Malus* *domestica*] |
| MD15G1058800 | PREDICTED: probable leucine-rich repeat receptor-like protein kinase At1g68400 [*Malus* *domestica*] |
| MD15G1060800 | PREDICTED: abscisic acid receptor PYL2-like [*Malus* *domestica*] |
| MD15G1063500 | PREDICTED: CSC1-like protein RXW8, partial [*Malus* *domestica*] |
| MD15G1064300 | PREDICTED: sodium-coupled neutral amino acid transporter 5-like [*Malus* *domestica*] |
| MD15G1066700 | PREDICTED: U-box domain-containing protein 30-like [*Malus* *domestica*] |
| MD15G1069200 | -- |
| MD15G1072300 | hypothetical protein PRUPE_1G424900 [*Prunus persica*] |
| MD15G1073900 | PREDICTED: peamaclein [*Malus* *domestica*] |
| MD15G1075400 | PREDICTED: cytochrome P450 81D11-like [*Malus* *domestica*] |
| MD15G1077000 | PREDICTED: probable isoaspartyl peptidase/L-asparaginase 2 [*Malus* *domestica*] |
| MD15G1077300 | PREDICTED: F-box protein At1g47056-like [*Malus* *domestica*] |
| MD15G1078200 | PREDICTED: probable WRKY transcription factor 7 [*Malus* *domestica*] |
| MD15G1078300 | PREDICTED: low-temperature-induced cysteine proteinase-like [*Malus* *domestica*] |
| MD15G1078600 | PREDICTED: uncharacterized protein LOC103455946 [*Malus* *domestica*] |
| MD15G1078900 | ethylene-responsive transcription factor ERF011-like [*Malus* *domestica*] |
| MD15G1079700 | PREDICTED: probable E3 ubiquitin-protein ligase RHG1A isoform X1 [*Malus* *domestica*] |
| MD15G1080900 | PREDICTED: uncharacterized protein LOC103455958 [*Malus* *domestica*] |
| MD15G1081100 | PREDICTED: transcription factor HEC2-like [*Malus* *domestica*] |
| MD15G1081300 | PREDICTED: uncharacterized protein LOC103431301 [*Malus* *domestica*] |
| MD15G1084900 | hypothetical protein PRUPE_1G438700 [*Prunus persica*] |
| MD15G1085100 | PREDICTED: succinate dehydrogenase subunit 5, mitochondrial-like [*Malus* *domestica*] |
| MD15G1086100 | glutamate dehydrogenase 1, mitochondrial-like isoform X3 [Dendrobium catenatum] |
| MD15G1096900 | PREDICTED: aspartate aminotransferase, cytoplasmic [*Malus* *domestica*] |
| MD15G1098400 | PREDICTED: 60S ribosomal protein L37-3-like [*Pyrus* x *bretschneideri*] |
| MD15G1098700 | PREDICTED: indole-3-pyruvate monooxygenase YUCCA6-like [*Malus* *domestica*] |
| MD15G1098800 | PREDICTED: BURP domain protein RD22 [*Pyrus* x *bretschneideri*] |
| MD15G1104100 | PREDICTED: putative glutathione-specific gamma-glutamylcyclotransferase 2 [*Malus* *domestica*] |
| MD15G1107300 | PREDICTED: uncharacterized protein LOC103456091 [*Malus* *domestica*] |
| MD15G1107900 | PREDICTED: E3 ubiquitin-protein ligase MBR2-like isoform X1 [*Malus* *domestica*] |
| MD15G1109100 | PREDICTED: uncharacterized aarF domain-containing protein kinase At4g31390, chloroplastic [*Malus* *domestica*] |
| MD15G1113000 | PREDICTED: glucan endo-1,3-beta-glucosidase 5 [*Malus* *domestica*] |
| MD15G1113900 | PREDICTED: E3 ubiquitin-protein ligase MIEL1-like [*Malus* *domestica*] |
| MD15G1116000 | PREDICTED: photosynthetic NDH subunit of subcomplex B 3, chloroplastic [*Malus* *domestica*] |
| MD15G1116900 | PREDICTED: probable 2-oxoglutarate-dependent dioxygenase AOP1 isoform X1 [*Malus* *domestica*] |
| MD15G1119900 | PREDICTED: cytochrome b-c1 complex subunit 7-2-like [*Malus* *domestica*] |
| MD15G1129300 | PREDICTED: uncharacterized protein LOC103456229 [*Malus* *domestica*] |
| MD15G1134100 | PREDICTED: signal recognition particle receptor subunit beta-like [*Pyrus* x *bretschneideri*] |
| MD15G1136300 | PREDICTED: lipase-like PAD4 isoform X1 [*Pyrus* x *bretschneideri*] |
| MD15G1136600 | PREDICTED: NAC domain-containing protein 2 [*Malus* *domestica*] |
| MD15G1137100 | PREDICTED: uncharacterized protein LOC103424870 [*Malus* *domestica*] |
| MD15G1137400 | PREDICTED: uncharacterized protein LOC103400280 [*Malus* *domestica*] |
| MD15G1139000 | PREDICTED: fatty acid amide hydrolase-like [*Malus* *domestica*] |
| MD15G1144400 | PREDICTED: 1-acyl-sn-glycerol-3-phosphate acyltransferase 1, chloroplastic-like [*Malus* *domestica*] |
| MD15G1153500 | CBL-interacting serine/threonine-protein kinase 6-like [*Malus* *domestica*] |
| MD15G1154600 | PREDICTED: transcription factor bHLH66-like [*Pyrus* x *bretschneideri*] |
| MD15G1157200 | PREDICTED: DCN1-like protein 5 isoform X1 [*Malus* *domestica*] |
| MD15G1158200 | PREDICTED: sugar transport protein 7 isoform X1 [*Malus* *domestica*] |
| MD15G1158600 | PREDICTED: uncharacterized protein LOC103400026 [*Malus* *domestica*] |
| MD15G1162400 | F-box/LRR-repeat protein 13-like [*Malus* *domestica*] |
| MD15G1169300 | PREDICTED: topless-related protein 4 [*Malus* *domestica*] |
| MD15G1170000 | PREDICTED: cucumber peeling cupredoxin-like [*Malus* *domestica*] |
| MD15G1170100 | PREDICTED: umecyanin-like [*Malus* *domestica*] |
| MD15G1170300 | PREDICTED: blue copper protein-like [*Malus* *domestica*] |
| MD15G1172200 | PREDICTED: uncharacterized protein LOC103431472 [*Malus* *domestica*] |
| MD15G1175900 | PREDICTED: zinc finger CCCH domain-containing protein 39-like [*Malus* *domestica*] |
| MD15G1177600 | PREDICTED: cytochrome P450 734A1-like [*Malus* *domestica*] |
| MD15G1178600 | PREDICTED: 3-ketoacyl-CoA synthase 11 [*Pyrus* x *bretschneideri*] |
| MD15G1185600 | kinesin-like protein KIN-13B [Hevea brasiliensis] |
| MD15G1186200 | PREDICTED: uncharacterized protein LOC100257802 isoform X1 [*Vitis vinifera*] |
| MD15G1187000 | hypothetical protein PRUPE_7G229500 [*Prunus persica*] |
| MD15G1187500 | PREDICTED: DNA mismatch repair protein MSH7-like [*Pyrus* x *bretschneideri*] |
| MD15G1188800 | PREDICTED: uncharacterized protein LOC103400574 [*Malus* *domestica*] |
| MD15G1188900 | PREDICTED: uncharacterized protein LOC103424953 [*Malus* *domestica*] |
| MD15G1189900 | -- |
| MD15G1191800 | PREDICTED: auxin-responsive protein IAA27-like [*Pyrus* x *bretschneideri*] |
| MD15G1194600 | PREDICTED: dentin sialophosphoprotein-like [*Malus* *domestica*] |
| MD15G1195000 | PREDICTED: uncharacterized protein LOC103400766 [*Malus* *domestica*] |
| MD15G1198900 | -- |
| MD15G1199000 | PREDICTED: pollen-specific protein SF21 isoform X2 [*Pyrus* x *bretschneideri*] |
| MD15G1203500 | 1-aminocyclopropane-1-carboxylic acid synthase 3a-2 [*Malus* *domestica*] |
| MD15G1203700 | PREDICTED: xaa-Pro dipeptidase [*Malus* *domestica*] |
| MD15G1204500 | PREDICTED: inactive protein kinase SELMODRAFT_444075-like isoform X1 [*Malus* *domestica*] |
| MD15G1206800 | RecName: Full=Beta-galactosidase; AltName: Full=Acid beta-galactosidase; Short=Lactase; AltName: Full=Exo-(1-- |
| MD15G1217100 | PREDICTED: uncharacterized protein LOC103400943 [*Malus* *domestica*] |
| MD15G1217900 | PREDICTED: thiol protease aleurain-like [*Malus* *domestica*] |
| MD15G1219200 | aldehyde dehydrogenase family 7 member A1 [*Malus* *domestica*] |
| MD15G1219900 | PREDICTED: inter-alpha-trypsin inhibitor heavy chain H3-like [*Malus* *domestica*] |
| MD15G1223500 | PREDICTED: sucrose synthase [*Malus* *domestica*] |
| MD15G1233700 | -- |
| MD15G1234100 | PREDICTED: probable acyl-activating enzyme 1, peroxisomal [*Malus* *domestica*] |
| MD15G1243800 | PREDICTED: thiosulfate sulfurtransferase 18-like [*Pyrus* x *bretschneideri*] |
| MD15G1249000 | PREDICTED: UPF0183 protein At3g51130 [*Pyrus* x *bretschneideri*] |
| MD15G1253900 | dehydrin 6 [*Malus* *domestica*] |
| MD15G1257200 | PREDICTED: uncharacterized protein LOC103401137 [*Malus* *domestica*] |
| MD15G1259200 | PREDICTED: ultraviolet-B receptor UVR8 [*Malus* *domestica*] |
| MD15G1263800 | PREDICTED: succinate dehydrogenase [ubiquinone] flavoprotein subunit, mitochondrial [*Malus* *domestica*] |
| MD15G1264700 | PREDICTED: F-box protein At5g07610-like [*Malus* *domestica*] |
| MD15G1277200 | PREDICTED: probable disease resistance protein At5g66900 [*Malus* *domestica*] |
| MD15G1283200 | PREDICTED: ethylene-responsive transcription factor ERF109-like [*Malus* *domestica*] |
| MD15G1283700 | PREDICTED: LOW QUALITY PROTEIN: heat stress transcription factor B-1 [*Malus* *domestica*] |
| MD15G1285300 | PREDICTED: subtilisin-like protease SBT1.9 [*Pyrus* x *bretschneideri*] |
| MD15G1286500 | PREDICTED: CBS domain-containing protein CBSX1, chloroplastic-like [*Malus* *domestica*] |
| MD15G1289500 | PREDICTED: probable receptor-like protein kinase At2g23200 [*Malus* *domestica*] |
| MD15G1291100 | -- |
| MD15G1292400 | PREDICTED: probable protein phosphatase 2C 78 [*Pyrus* x *bretschneideri*] |
| MD15G1296300 | PREDICTED: ruvB-like 2 [*Malus* *domestica*] |
| MD15G1297000 | PREDICTED: adenylyl-sulfate kinase 1, chloroplastic-like isoform X1 [*Malus* *domestica*] |
| MD15G1298700 | PREDICTED: heptahelical transmembrane protein 4-like [*Pyrus* x *bretschneideri*] |
| MD15G1302200 | 1-aminocyclopropane-1-carboxylate synthase [*Malus* *domestica*] |
| MD15G1303500 | PREDICTED: probable xyloglucan endotransglucosylase/hydrolase protein 6 [*Pyrus* x *bretschneideri*] |
| MD15G1304000 | PREDICTED: cytochrome c oxidase subunit 6a, mitochondrial-like [*Pyrus* x *bretschneideri*] |
| MD15G1304600 | PREDICTED: LRR receptor-like serine/threonine-protein kinase HSL2 [*Pyrus* x *bretschneideri*] |
| MD15G1308800 | PREDICTED: probable mannitol dehydrogenase [*Pyrus* x *bretschneideri*] |
| MD15G1309900 | PREDICTED: probable 1-deoxy-D-xylulose-5-phosphate synthase 2, chloroplastic isoform X1 [*Malus* *domestica*] |
| MD15G1315000 | PREDICTED: mitochondrial uncoupling protein 5-like [*Malus* *domestica*] |
| MD15G1332800 | PREDICTED: uncharacterized protein LOC103455056 [*Malus* *domestica*] |
| MD15G1343400 | PREDICTED: probable receptor-like protein kinase At1g11050 [*Malus* *domestica*] |
| MD15G1343900 | PREDICTED: amino acid permease 3-like [*Pyrus* x *bretschneideri*] |
| MD15G1344600 | PREDICTED: uncharacterized protein LOC103935886 [*Pyrus* x *bretschneideri*] |
| MD15G1348300 | PREDICTED: uncharacterized protein At4g08330, chloroplastic-like [*Malus* *domestica*] |
| MD15G1348800 | PREDICTED: esterase-like [*Pyrus* x *bretschneideri*] |
| MD15G1348900 | PREDICTED: alpha-L-fucosidase 3-like [*Malus* *domestica*] |
| MD15G1353900 | PREDICTED: 60S ribosomal protein L38-like [*Malus* *domestica*] |
| MD15G1357000 | PREDICTED: oxysterol-binding protein-related protein 1C-like isoform X2 [*Malus* *domestica*] |
| MD15G1358800 | PREDICTED: beta-1,3-galactosyltransferase 7-like, partial [*Malus* *domestica*] |
| MD15G1365300 | PREDICTED: uncharacterized WD repeat-containing protein C2A9.03-like [*Malus* *domestica*] |
| MD15G1373100 | -- |
| MD15G1374500 | homeobox-leucine zipper protein ATHB-6-like [*Malus* *domestica*] |
| MD15G1380500 | PREDICTED: dihydrofolate reductase-like [*Malus* *domestica*] |
| MD15G1383800 | PREDICTED: universal stress protein PHOS34-like [*Malus* *domestica*] |
| MD15G1384500 | dormancy-associated MADS box 1 protein [*Malus* *domestica*] |
| MD15G1386300 | PREDICTED: protein ECERIFERUM 1-like isoform X2 [*Malus* *domestica*] |
| MD15G1391900 | PREDICTED: uncharacterized protein At5g65660-like [*Malus* *domestica*] |
| MD15G1397600 | PREDICTED: 11-beta-hydroxysteroid dehydrogenase-like 5 [*Pyrus* x *bretschneideri*] |
| MD15G1403200 | PREDICTED: elongation factor 1-gamma-like [*Pyrus* x *bretschneideri*] |
| MD15G1411500 | PREDICTED: 3-hydroxyacyl-[acyl-carrier-protein] dehydratase FabZ-like [*Malus* *domestica*] |
| MD15G1411800 | PREDICTED: non-specific lipid-transfer protein-like protein At5g64080 [*Malus* *domestica*] |
| MD15G1412200 | PREDICTED: aminotransferase ALD1-like isoform X1 [*Malus* *domestica*] |
| MD15G1412400 | PREDICTED: aminotransferase ALD1-like isoform X2 [*Pyrus* x *bretschneideri*] |
| MD15G1413800 | -- |
| MD15G1415200 | PREDICTED: cellulose synthase-like protein G2 isoform X2 [*Malus* *domestica*] |
| MD15G1416400 | PREDICTED: actin-related protein 2/3 complex subunit 5A-like [*Malus* *domestica*] |
| MD15G1417600 | PREDICTED: uncharacterized protein LOC103402334 isoform X1 [*Malus* *domestica*] |
| MD15G1420800 | PREDICTED: 3-oxoacyl-[acyl-carrier-protein] reductase FabG-like [*Malus* *domestica*] |
| MD15G1420900 | PREDICTED: 3-oxoacyl-[acyl-carrier-protein] reductase FabG-like [*Malus* *domestica*] |
| MD15G1433600 | PREDICTED: nifU-like protein 2, chloroplastic [*Malus* *domestica*] |
| MD15G1438300 | PREDICTED: uncharacterized protein LOC103431793 [*Malus* *domestica*] |
| MD15G1439100 | PREDICTED: ras-related protein RABD1-like [*Malus* *domestica*] |
| MD15G1440200 | PREDICTED: uncharacterized protein At5g43822-like [*Pyrus* x *bretschneideri*] |
| MD15G1440400 | PREDICTED: non-lysosomal glucosylceramidase-like isoform X3 [*Pyrus* x *bretschneideri*] |
| MD15G1440700 | PREDICTED: uncharacterized protein At5g08430-like isoform X2 [*Malus* *domestica*] |
| MD15G1441700 | PREDICTED: probable polygalacturonase [*Malus* *domestica*] |
| MD16G1000300 | PREDICTED: tricalbin-3-like [*Malus* *domestica*] |
| MD16G1000500 | PREDICTED: anamorsin homolog [*Malus* *domestica*] |
| MD16G1007100 | PREDICTED: inactive leucine-rich repeat receptor-like serine/threonine-protein kinase At1g60630 [*Malus* *domestica*] |
| MD16G1013600 | PREDICTED: purple acid phosphatase 3 [*Pyrus* x *bretschneideri*] |
| MD16G1014100 | PREDICTED: uncharacterized protein LOC103402673 [*Malus* *domestica*] |
| MD16G1019200 | PREDICTED: probable calcium-binding protein CML25 [*Malus* *domestica*] |
| MD16G1019500 | PREDICTED: nudix hydrolase 17, mitochondrial [*Malus* *domestica*] |
| MD16G1019900 | PREDICTED: LOW QUALITY PROTEIN: 1-aminocyclopropane-1-carboxylate oxidase homolog 4-like [*Malus* *domestica*] |
| MD16G1020000 | PREDICTED: 1-aminocyclopropane-1-carboxylate oxidase homolog 4-like [*Malus* *domestica*] |
| MD16G1021100 | PREDICTED: dynein light chain 1, cytoplasmic [*Pyrus* x *bretschneideri*] |
| MD16G1029200 | PREDICTED: uncharacterized protein At1g66480 [*Malus* *domestica*] |
| MD16G1030600 | PREDICTED: uncharacterized protein LOC103403218 [*Malus* *domestica*] |
| MD16G1033800 | PREDICTED: protein DETOXIFICATION 16-like [*Malus* *domestica*] |
| MD16G1035000 | PREDICTED: CDP-diacylglycerol--serine O-phosphatidyltransferase 1 [*Malus* *domestica*] |
| MD16G1035500 | PREDICTED: E3 ubiquitin-protein ligase RHA2A-like [*Malus* *domestica*] |
| MD16G1036500 | PREDICTED: lipid phosphate phosphatase 1 isoform X1 [*Malus* *domestica*] |
| MD16G1037100 | PREDICTED: probable inactive leucine-rich repeat receptor-like protein kinase At1g66830 [*Malus* *domestica*] |
| MD16G1037500 | PREDICTED: uncharacterized protein LOC103402658 [*Malus* *domestica*] |
| MD16G1040800 | transcription factor MYB21 [*Malus* *domestica*] |
| MD16G1041000 | PREDICTED: acyl-acyl carrier protein thioesterase ATL3, chloroplastic-like [*Malus* *domestica*] |
| MD16G1043200 | PREDICTED: homocysteine S-methyltransferase 1 [*Malus* *domestica*] |
| MD16G1045500 | -- |
| MD16G1050300 | PREDICTED: phosphoenolpyruvate carboxylase 4-like [*Malus* *domestica*] |
| MD16G1051100 | PREDICTED: putative phospholipid-transporting ATPase 9 [*Pyrus* x *bretschneideri*] |
| MD16G1051600 | PREDICTED: E3 ubiquitin-protein ligase RNF181-like [*Pyrus* x *bretschneideri*] |
| MD16G1053000 | -- |
| MD16G1055900 | D-aminoacyl-tRNA deacylase [*Prunus avium*] |
| MD16G1056600 | PREDICTED: cytochrome P450 CYP749A22-like [*Malus* *domestica*] |
| MD16G1056700 | cytochrome P450 CYP749A22-like [*Prunus persica*] |
| MD16G1057800 | PREDICTED: uncharacterized protein LOC103431834 [*Malus* *domestica*] |
| MD16G1059500 | PREDICTED: probable 6-phosphogluconolactonase 1 [*Malus* *domestica*] |
| MD16G1062000 | PREDICTED: uncharacterized protein LOC103402829 isoform X1 [*Malus* *domestica*] |
| MD16G1065200 | PREDICTED: uncharacterized protein LOC103416500 [*Malus* *domestica*] |
| MD16G1069800 | PREDICTED: uncharacterized endoplasmic reticulum membrane protein C16E8.02-like [*Malus* *domestica*] |
| MD16G1072700 | PREDICTED: uncharacterized protein LOC103402896 isoform X2 [*Malus* *domestica*] |
| MD16G1072800 | PREDICTED: zinc-finger homeodomain protein 11-like [*Malus* *domestica*] |
| MD16G1072900 | PREDICTED: uncharacterized protein LOC103402899 [*Malus* *domestica*] |
| MD16G1074400 | PREDICTED: haloacid dehalogenase-like hydrolase domain-containing protein 3 [*Malus* *domestica*] |
| MD16G1075600 | PREDICTED: peptidyl-prolyl cis-trans isomerase CYP23-like [*Malus* *domestica*] |
| MD16G1081100 | PREDICTED: glutathione S-transferase U17-like [*Malus* *domestica*] |
| MD16G1081700 | PREDICTED: glycerol-3-phosphate 2-O-acyltransferase 6-like [*Malus* *domestica*] |
| MD16G1083600 | PREDICTED: microtubule-associated protein TORTIFOLIA1-like [*Malus* *domestica*] |
| MD16G1088900 | PREDICTED: WAT1-related protein At4g15540-like isoform X2 [*Malus* *domestica*] |
| MD16G1089900 | PREDICTED: potassium transporter 6-like [*Malus* *domestica*] |
| MD16G1090000 | PREDICTED: potassium transporter 6-like [*Malus* *domestica*] |
| MD16G1095200 | PREDICTED: phosphatidylinositol 3-kinase, root isoform-like [*Malus* *domestica*] |
| MD16G1096500 | PREDICTED: F-box/kelch-repeat protein At1g23390-like [*Malus* *domestica*] |
| MD16G1096600 | PREDICTED: cysteine-rich receptor-like protein kinase 10 [*Malus* *domestica*] |
| MD16G1109100 | PREDICTED: salutaridinol 7-O-acetyltransferase-like [*Malus* *domestica*] |
| MD16G1111600 | PREDICTED: 3-oxoacyl-[acyl-carrier-protein] reductase 4-like [*Pyrus* x *bretschneideri*] |
| MD16G1113200 | lipoxygenase [*Malus* *domestica*] |
| MD16G1117200 | PREDICTED: uncharacterized protein LOC103403148 isoform X1 [*Malus* *domestica*] |
| MD16G1117300 | PREDICTED: mannosyl-oligosaccharide glucosidase GCS1-like [*Malus* *domestica*] |
| MD16G1123700 | PREDICTED: ACT domain-containing protein ACR4-like [*Malus* *domestica*] |
| MD16G1124300 | PREDICTED: auxin-responsive protein SAUR32-like [*Malus* *domestica*] |
| MD16G1125300 | PREDICTED: bidirectional sugar transporter N3-like [*Malus* *domestica*] |
| MD16G1125800 | NAC transcription factor 25-like [*Malus* *domestica*] |
| MD16G1128500 | PREDICTED: uncharacterized protein LOC103433185 [*Malus* *domestica*] |
| MD16G1129900 | PREDICTED: light-regulated protein [*Malus* *domestica*] |
| MD16G1130800 | PREDICTED: eukaryotic peptide chain release factor subunit 1-3-like [*Malus* *domestica*] |
| MD16G1131300 | PREDICTED: uncharacterized protein LOC103433115 [*Malus* *domestica*] |
| MD16G1134300 | PREDICTED: uncharacterized protein LOC103441518 [*Malus* *domestica*] |
| MD16G1140700 | PREDICTED: TMV resistance protein N-like [*Malus* *domestica*] |
| MD16G1143100 | PREDICTED: nudix hydrolase 13, mitochondrial-like isoform X1 [*Malus* *domestica*] |
| MD16G1144900 | PREDICTED: uncharacterized protein LOC103433137 [*Malus* *domestica*] |
| MD16G1145200 | PREDICTED: probable xyloglucan endotransglucosylase/hydrolase protein B isoform X1 [*Malus* *domestica*] |
| MD16G1146100 | ras-related protein Rab7 isoform X1 [*Prunus persica*] |
| MD16G1146300 | PREDICTED: uncharacterized protein LOC108170056 [*Malus* *domestica*] |
| MD16G1149300 | PREDICTED: desiccation-related protein PCC13-62-like [*Malus* *domestica*] |
| MD16G1149400 | PREDICTED: desiccation-related protein PCC13-62-like [*Malus* *domestica*] |
| MD16G1150200 | PREDICTED: cell wall / vacuolar inhibitor of fructosidase 1 [*Pyrus* x *bretschneideri*] |
| MD16G1151000 | PREDICTED: probable WRKY transcription factor 48 [*Malus* *domestica*] |
| MD16G1158200 | PREDICTED: acetate/butyrate--CoA ligase AAE7, peroxisomal [*Malus* *domestica*] |
| MD16G1158300 | putative beta-D-xylosidase precursor [*Malus* *domestica*] |
| MD16G1160700 | major allergen Mal d 1 [*Malus* *domestica*] |
| MD16G1162800 | ethylene-responsive transcription factor RAP2-3 [*Malus* *domestica*] |
| MD16G1163400 | -- |
| MD16G1164100 | PREDICTED: probable acyl-CoA dehydrogenase IBR3 [*Malus* *domestica*] |
| MD16G1164900 | PREDICTED: bifunctional epoxide hydrolase 2-like [*Malus* *domestica*] |
| MD16G1170100 | PREDICTED: putative F-box protein At1g67623 [*Malus* *domestica*] |
| MD16G1170500 | PREDICTED: CAP-Gly domain-containing linker protein 1 [*Malus* *domestica*] |
| MD16G1176100 | PREDICTED: acyl carrier protein 1, chloroplastic-like isoform X2 [*Malus* *domestica*] |
| MD16G1177400 | PREDICTED: NADH dehydrogenase [ubiquinone] 1 alpha subcomplex subunit 8-B-like [*Pyrus* x *bretschneideri*] |
| MD16G1178700 | -- |
| MD16G1180300 | PREDICTED: F-box/LRR-repeat protein 4-like isoform X2 [*Malus* *domestica*] |
| MD16G1182200 | PREDICTED: endo-1,3;1,4-beta-D-glucanase-like [*Malus* *domestica*] |
| MD16G1185400 | PREDICTED: basic 7S globulin [*Malus* *domestica*] |
| MD16G1189900 | PREDICTED: sugar transport protein 13-like [*Malus* *domestica*] |
| MD16G1198500 | PREDICTED: uncharacterized protein LOC103414674 isoform X1 [*Malus* *domestica*] |
| MD16G1201400 | PREDICTED: ATP sulfurylase 1, chloroplastic [*Malus* *domestica*] |
| MD16G1206500 | ARF domain class transcription factor [*Malus* *domestica*] |
| MD16G1217200 | PREDICTED: serine/threonine-protein phosphatase PP1 isozyme 2-like [*Malus* *domestica*] |
| MD16G1217400 | PREDICTED: probable inorganic phosphate transporter 1-7 [*Malus* *domestica*] |
| MD16G1217500 | PREDICTED: phosphoenolpyruvate carboxylase kinase 1-like [*Malus* *domestica*] |
| MD16G1225600 | PREDICTED: uncharacterized protein LOC103403586 [*Malus* *domestica*] |
| MD16G1231800 | PREDICTED: L10-interacting MYB domain-containing protein-like [*Malus* *domestica*] |
| MD16G1249800 | PREDICTED: uncharacterized protein LOC103426676 isoform X1 [*Malus* *domestica*] |
| MD16G1251700 | PREDICTED: uncharacterized protein LOC103450571 isoform X6 [*Malus* *domestica*] |
| MD16G1257700 | PREDICTED: probable small nuclear ribonucleoprotein F [*Malus* *domestica*] |
| MD16G1262900 | PREDICTED: probable E3 ubiquitin-protein ligase XBOS32 [*Pyrus* x *bretschneideri*] |
| MD16G1267200 | xyloglucan endotransglucosylase/hydrolase 8 [*Malus* *domestica*] |
| MD16G1269100 | PREDICTED: iron-sulfur assembly protein IscA-like 2, mitochondrial isoform X2 [*Pyrus* x *bretschneideri*] |
| MD16G1269600 | PREDICTED: probable methyltransferase PMT14 [*Malus* *domestica*] |
| MD16G1275100 | PREDICTED: uncharacterized protein LOC103440243 [*Malus* *domestica*] |
| MD16G1286700 | PREDICTED: uncharacterized protein LOC103454334 [*Malus* *domestica*] |
| MD17G1001600 | PREDICTED: 3-oxoacyl-[acyl-carrier-protein] reductase FabG-like [*Malus* *domestica*] |
| MD17G1001700 | PREDICTED: ABC transporter I family member 11, chloroplastic-like isoform X1 [*Malus* *domestica*] |
| MD17G1013800 | PREDICTED: triacylglycerol lipase 2-like [*Malus* *domestica*] |
| MD17G1014700 | PREDICTED: protoporphyrinogen oxidase, mitochondrial-like [*Malus* *domestica*] |
| MD17G1019200 | PREDICTED: cystinosin homolog [*Malus* *domestica*] |
| MD17G1021800 | PREDICTED: uncharacterized protein LOC103404255 isoform X1 [*Malus* *domestica*] |
| MD17G1022300 | PREDICTED: glucuronokinase 1 isoform X1 [*Malus* *domestica*] |
| MD17G1027800 | PREDICTED: serine/threonine-protein phosphatase 7 long form homolog [*Pyrus* x *bretschneideri*] |
| MD17G1030400 | PREDICTED: uncharacterized protein LOC103404310 isoform X2 [*Malus* *domestica*] |
| MD17G1031300 | PREDICTED: isocitrate dehydrogenase [NADP]-like isoform X1 [*Pyrus* x *bretschneideri*] |
| MD17G1031500 | PREDICTED: tRNA (adenine(58)-N(1))-methyltransferase catalytic subunit TRMT61A-like [*Pyrus* x *bretschneideri*] |
| MD17G1040300 | PREDICTED: zinc finger AN1 domain-containing stress-associated protein 12-like [*Malus* *domestica*] |
| MD17G1048300 | PREDICTED: plant cysteine oxidase 2-like [*Malus* *domestica*] |
| MD17G1049200 | PREDICTED: binding partner of ACD11 1-like [*Malus* *domestica*] |
| MD17G1049300 | PREDICTED: transcription factor PRE6-like [*Malus* *domestica*] |
| MD17G1052800 | PREDICTED: uncharacterized protein At5g39570-like [*Malus* *domestica*] |
| MD17G1054300 | PREDICTED: lamin-like protein [*Malus* *domestica*] |
| MD17G1058900 | PREDICTED: uncharacterized protein LOC103429153 [*Malus* *domestica*] |
| MD17G1059000 | PREDICTED: uncharacterized protein Mb2734 [*Malus* *domestica*] |
| MD17G1059300 | PREDICTED: uncharacterized protein LOC103429153 [*Malus* *domestica*] |
| MD17G1059400 | PREDICTED: exocyst complex component EXO70B1-like isoform X1 [*Pyrus* x *bretschneideri*] |
| MD17G1060100 | PREDICTED: LRR receptor-like serine/threonine-protein kinase RPK2 [*Malus* *domestica*] |
| MD17G1061600 | PREDICTED: putative methylesterase 11, chloroplastic [*Malus* *domestica*] |
| MD17G1063000 | MRLK4, partial [*Malus* *domestica*] |
| MD17G1064800 | PREDICTED: cytochrome P450 85A [*Pyrus* x *bretschneideri*] |
| MD17G1068100 | PREDICTED: E3 ubiquitin-protein ligase CIP8-like [*Malus* *domestica*] |
| MD17G1069200 | PREDICTED: protein GLUTAMINE DUMPER 1-like [*Pyrus* x *bretschneideri*] |
| MD17G1069700 | PREDICTED: F-box/FBD/LRR-repeat protein At1g13570-like [*Malus* *domestica*] |
| MD17G1070400 | putative S-adenosylmethionine decarboxylase, partial [Prunus dulcis] |
| MD17G1078600 | PREDICTED: LOB domain-containing protein 41-like [*Pyrus* x *bretschneideri*] |
| MD17G1080400 | PREDICTED: S-linalool synthase-like isoform X1 [*Malus* *domestica*] |
| MD17G1085500 | PREDICTED: protein THYLAKOID FORMATION1, chloroplastic [*Malus* *domestica*] |
| MD17G1095200 | PREDICTED: uncharacterized protein LOC103404804 [*Malus* *domestica*] |
| MD17G1097100 | -- |
| MD17G1097800 | PREDICTED: autophagy-related protein 8C-like [*Malus* *domestica*] |
| MD17G1099600 | PREDICTED: cellulose synthase-like protein H1 [*Malus* *domestica*] |
| MD17G1101700 | -- |
| MD17G1102000 | PREDICTED: ribonuclease 3-like protein 3 [*Pyrus* x *bretschneideri*] |
| MD17G1112700 | scarecrow-like transcription factor PAT1 [*Malus* *domestica*] |
| MD17G1117800 | PREDICTED: LOW QUALITY PROTEIN: acetyl-CoA acetyltransferase, cytosolic 1-like [*Malus* *domestica*] |
| MD17G1120800 | -- |
| MD17G1123100 | PREDICTED: vacuolar cation/proton exchanger 3-like [*Malus* *domestica*] |
| MD17G1124900 | PREDICTED: 7-deoxyloganetin glucosyltransferase-like [*Malus* *domestica*] |
| MD17G1125000 | PREDICTED: 7-deoxyloganetin glucosyltransferase-like [*Pyrus* x *bretschneideri*] |
| MD17G1125900 | PREDICTED: 7-deoxyloganetin glucosyltransferase-like [*Malus* *domestica*] |
| MD17G1127900 | PREDICTED: cysteine-rich and transmembrane domain-containing protein A-like isoform X1 [*Malus* *domestica*] |
| MD17G1134800 | PREDICTED: 4-hydroxyphenylpyruvate dioxygenase-like [*Malus* *domestica*] |
| MD17G1135600 | PREDICTED: aspartyl protease family protein 2-like [*Pyrus* x *bretschneideri*] |
| MD17G1136100 | hypothetical protein PRUPE_3G176700 [*Prunus persica*] |
| MD17G1138200 | PREDICTED: uncharacterized protein LOC103405173 [*Malus* *domestica*] |
| MD17G1140000 | brassinosteroid-regulated protein BRU1-like precursor [*Malus* *domestica*] |
| MD17G1143900 | PREDICTED: D-3-phosphoglycerate dehydrogenase 2, chloroplastic-like [*Malus* *domestica*] |
| MD17G1154800 | PREDICTED: RING-H2 finger protein ATL3-like [*Malus* *domestica*] |
| MD17G1167100 | lipoxygenase [*Malus* *domestica*] |
| MD17G1178700 | PREDICTED: IAA-amino acid hydrolase ILR1-like 4 [*Pyrus* x *bretschneideri*] |
| MD17G1178800 | PREDICTED: uncharacterized protein LOC103453537 [*Malus* *domestica*] |
| MD17G1187100 | -- |
| MD17G1197100 | PREDICTED: uncharacterized acetyltransferase At3g50280-like [*Malus* *domestica*] |
| MD17G1202000 | PREDICTED: uncharacterized protein LOC103440692 [*Malus* *domestica*] |
| MD17G1204600 | PREDICTED: uncharacterized protein LOC103940691 [*Pyrus* x *bretschneideri*] |
| MD17G1209300 | PREDICTED: uncharacterized protein LOC103437261 [*Malus* *domestica*] |
| MD17G1215800 | PREDICTED: arogenate dehydrogenase 2, chloroplastic-like [*Malus* *domestica*] |
| MD17G1216000 | PREDICTED: NADH dehydrogenase [ubiquinone] flavoprotein 2, mitochondrial-like [*Malus* *domestica*] |
| MD17G1218600 | PREDICTED: uncharacterized protein LOC103436057 [*Malus* *domestica*] |
| MD17G1222400 | PREDICTED: cinnamoyl-CoA reductase 1-like [*Malus* *domestica*] |
| MD17G1222500 | PREDICTED: metal transporter Nramp6-like [*Malus* *domestica*] |
| MD17G1229400 | p-coumarate:CoA ligase 1 [Sorbus aucuparia] |
| MD17G1231900 | PREDICTED: ketol-acid reductoisomerase, chloroplastic [*Malus* *domestica*] |
| MD17G1238200 | PREDICTED: O-acyltransferase WSD1-like [*Pyrus* x *bretschneideri*] |
| MD17G1240600 | PREDICTED: phosphomevalonate kinase-like [*Pyrus* x *bretschneideri*] |
| MD17G1241600 | PREDICTED: uncharacterized protein LOC103405667 [*Malus* *domestica*] |
| MD17G1241900 | PREDICTED: probable polyamine transporter At1g31830 isoform X1 [*Malus* *domestica*] |
| MD17G1242500 | PREDICTED: tetraspanin-19 [*Pyrus* x *bretschneideri*] |
| MD17G1248100 | PREDICTED: G-type lectin S-receptor-like serine/threonine-protein kinase At1g61500 isoform X1 [*Malus* *domestica*] |
| MD17G1249500 | PREDICTED: G-type lectin S-receptor-like serine/threonine-protein kinase At1g61500 isoform X1 [*Malus* *domestica*] |
| MD17G1249900 | PREDICTED: lisH domain and HEAT repeat-containing protein KIAA1468 homolog [*Pyrus* x *bretschneideri*] |
| MD17G1262000 | PREDICTED: uncharacterized protein LOC103411304 [*Malus* *domestica*] |
| MD17G1264900 | PREDICTED: aspartate aminotransferase, mitochondrial [*Malus* *domestica*] |
| MD17G1267400 | PREDICTED: microsomal glutathione S-transferase 3-like [*Malus* *domestica*] |
| MD17G1267600 | PREDICTED: microsomal glutathione S-transferase 3-like [*Malus* *domestica*] |
| MD17G1268300 | PREDICTED: COX assembly mitochondrial protein 2 homolog [*Malus* *domestica*] |
| MD17G1272100 | glutathione S-transferase F12-like [*Malus* *domestica*] |
| MD17G1275600 | PREDICTED: uncharacterized protein LOC103417503 [*Malus* *domestica*] |
| MD17G1279900 | PREDICTED: boron transporter 1-like [*Malus* *domestica*] |
| MD17G1284100 | PREDICTED: uncharacterized protein LOC103426243 isoform X2 [*Malus* *domestica*] |
| MD17G1286100 | PREDICTED: uncharacterized protein LOC103405980 [*Malus* *domestica*] |
| *Malus*_*domestica*_newGene_10085 | PREDICTED: uncharacterized protein LOC101257369 [Solanum lycopersicum] |
| *Malus*_*domestica*_newGene_10510 | PREDICTED: uncharacterized protein LOC103454795 [*Malus* *domestica*] |
| *Malus*_*domestica*_newGene_10597 | PREDICTED: ethylene-responsive transcription factor ERF113-like isoform X1 [*Pyrus* x *bretschneideri*] |
| *Malus*_*domestica*_newGene_10723 | PREDICTED: ubiquitin-conjugating enzyme E2 4-like [*Malus* *domestica*] |
| *Malus*_*domestica*_newGene_110 | PREDICTED: ribosomal L1 domain-containing protein 1-like [*Malus* *domestica*] |
| *Malus*_*domestica*_newGene_11371 | PREDICTED: uncharacterized protein LOC103429153 [*Malus* *domestica*] |
| *Malus*_*domestica*_newGene_11428 | hypothetical protein MTR_7g007390 [Medicago truncatula] |
| *Malus*_*domestica*_newGene_11710 | PREDICTED: syntaxin-52-like isoform X3 [*Malus* *domestica*] |
| *Malus*_*domestica*_newGene_11847 | PREDICTED: uncharacterized protein LOC103449032 isoform X4 [*Malus* *domestica*] |
| *Malus*_*domestica*_newGene_11851 | PREDICTED: high mobility group B protein 3-like [*Pyrus* x *bretschneideri*] |
| *Malus*_*domestica*_newGene_11879 | -- |
| *Malus*_*domestica*_newGene_12281 | PREDICTED: uncharacterized protein LOC103961899 [*Pyrus* x *bretschneideri*] |
| *Malus*_*domestica*_newGene_12482 | PREDICTED: uncharacterized protein LOC103438477 [*Malus* *domestica*] |
| *Malus*_*domestica*_newGene_12611 | -- |
| *Malus*_*domestica*_newGene_1310 | -- |
| *Malus*_*domestica*_newGene_1451 | PREDICTED: signal recognition particle 43 kDa protein, chloroplastic [*Pyrus* x *bretschneideri*] |
| *Malus*_*domestica*_newGene_1502 | PREDICTED: GPI ethanolamine phosphate transferase 1-like [*Pyrus* x *bretschneideri*] |
| *Malus*_*domestica*_newGene_1675 | -- |
| *Malus*_*domestica*_newGene_1737 | PREDICTED: LOW QUALITY PROTEIN: ABC transporter G family member 32-like, partial [*Malus* *domestica*] |
| *Malus*_*domestica*_newGene_2302 | PREDICTED: uncharacterized protein LOC103935787 [*Pyrus* x *bretschneideri*] |
| *Malus*_*domestica*_newGene_2431 | PREDICTED: uncharacterized protein LOC103437447 [*Malus* *domestica*] |
| *Malus*_*domestica*_newGene_2671 | -- |
| *Malus*_*domestica*_newGene_2871 | -- |
| *Malus*_*domestica*_newGene_3433 | -- |
| *Malus*_*domestica*_newGene_361 | -- |
| *Malus*_*domestica*_newGene_3655 | PREDICTED: uncharacterized protein LOC102601005 [Solanum tuberosum] |
| *Malus*_*domestica*_newGene_3812 | PREDICTED: basic leucine zipper 9-like [*Malus* *domestica*] |
| *Malus*_*domestica*_newGene_4256 | -- |
| *Malus*_*domestica*_newGene_4308 | PREDICTED: uncharacterized protein LOC103952605 [*Pyrus* x *bretschneideri*] |
| *Malus*_*domestica*_newGene_4637 | PREDICTED: uncharacterized protein LOC103419223 [*Malus* *domestica*] |
| *Malus*_*domestica*_newGene_4731 | PREDICTED: triacylglycerol lipase 2-like [*Pyrus* x *bretschneideri*] |
| *Malus*_*domestica*_newGene_4734 | PREDICTED: uncharacterized protein LOC103405083 [*Malus* *domestica*] |
| *Malus*_*domestica*_newGene_4750 | -- |
| *Malus*_*domestica*_newGene_4922 | PREDICTED: uncharacterized protein LOC103446305 [*Malus* *domestica*] |
| *Malus*_*domestica*_newGene_5434 | PREDICTED: GDP-L-galactose phosphorylase 2-like [*Malus* *domestica*] |
| *Malus*_*domestica*_newGene_5482 | hypothetical protein CISIN_1g030092mg [Citrus sinensis] |
| *Malus*_*domestica*_newGene_5604 | PREDICTED: 3-hydroxyisobutyryl-CoA hydrolase-like protein 4, mitochondrial isoform X2 [*Malus* *domestica*] |
| *Malus*_*domestica*_newGene_5613 | -- |
| *Malus*_*domestica*_newGene_7217 | PREDICTED: uncharacterized protein LOC101302821 [Fragaria vesca subsp. vesca] |
| *Malus*_*domestica*_newGene_7350 | PREDICTED: serine/threonine-protein kinase PRP4 homolog isoform X1 [*Malus* *domestica*] |
| *Malus*_*domestica*_newGene_7503 | -- |
| *Malus*_*domestica*_newGene_7591 | hypothetical protein CICLE_v10007195mg [Citrus clementina] |
| *Malus*_*domestica*_newGene_772 | PREDICTED: uncharacterized protein LOC103956517 isoform X2 [*Pyrus* x *bretschneideri*] |
| *Malus*_*domestica*_newGene_791 | -- |
| *Malus*_*domestica*_newGene_8201 | -- |
| *Malus*_*domestica*_newGene_8221 | hypothetical protein PRUPE_ppa023588mg [*Prunus persica*] |
| *Malus*_*domestica*_newGene_8476 | PREDICTED: uncharacterized protein LOC103453021, partial [*Malus* *domestica*] |
| *Malus*_*domestica*_newGene_8493 | -- |
| *Malus*_*domestica*_newGene_8578 | PREDICTED: zinc finger MYM-type protein 1-like [*Malus* *domestica*] |
| *Malus*_*domestica*_newGene_8769 | PREDICTED: uncharacterized protein LOC103965021 [*Pyrus* x *bretschneideri*] |
| *Malus*_*domestica*_newGene_8818 | PREDICTED: uncharacterized protein LOC103442161 [*Malus* *domestica*] |
| *Malus*_*domestica*_newGene_8880 | PREDICTED: uncharacterized protein LOC103402228 [*Malus* *domestica*] |
| *Malus*_*domestica*_newGene_9136 | -- |
| *Malus*_*domestica*_newGene_9152 | PREDICTED: phosphatidylinositol 4-kinase gamma 4-like isoform X1 [*Pyrus* x *bretschneideri*] |
| *Malus*_*domestica*_newGene_9337 | PREDICTED: uncharacterized protein LOC103967730 [*Pyrus* x *bretschneideri*] |
| *Malus*_*domestica*_newGene_9472 | PREDICTED: nucleolin-like [*Pyrus* x *bretschneideri*] |
| *Malus*_*domestica*_newGene_9675 | PREDICTED: LOW QUALITY PROTEIN: probable threonine--tRNA ligase, cytoplasmic [*Malus* *domestica*] |
| *Malus*_*domestica*_newGene_9991 | PREDICTED: putative transferase CAF17 homolog, mitochondrial isoform X1 [*Malus* *domestica*] |

| **Table S3.** DNA primers used in this study. | |  |
| --- | --- | --- |
| **Primer name** | **Primer Sequence(5'-3')** | **Used for** |
| 18S RNA-F | GTCACTACCTCCCCGTGTCA | RT-qPCR analysis |
| 18S RNA-R | GAGCCTGAGAAACGGCTACC | RT-qPCR analysis |
| qPAL-F | ACCCTGGACAGATTGAGGCAGCT | RT-qPCR analysis |
| qPAL-R | GCGTAGCGATCCTGCTTTGGCT | RT-qPCR analysis |
| qCHS-F | GTGACTGTCCAGGAAGTTCGC | RT-qPCR analysis |
| qCHS-R | GCACACACTTGGATTCTCCTTTAG | RT-qPCR analysis |
| qCHI-F | GAAGGGTAAGACCGCCGAG | RT-qPCR analysis |
| qCHI-R | CACAATTCTCCGAAACTTTCTCAG | RT-qPCR analysis |
| qF3H-F | CGGGATGATGGGAAAACG | RT-qPCR analysis |
| qF3H-R | CGCTGGGTTCTGGAATGTG | RT-qPCR analysis |
| qF3'H-F | ACGATGGCGGATGTTACGG | RT-qPCR analysis |
| qF3'H-R | GCTTTGACCCTGCACTTGCT | RT-qPCR analysis |
| qDFR-F | GGACCCCGAGAATGAAGTG | RT-qPCR analysis |
| qDFR-R | CTCCACATTCACGGTTCCTG | RT-qPCR analysis |
| qANS-F | GAGAAGTATGCCAATGACCAGG | RT-qPCR analysis |
| qANS-R | GGCGGTTGCCTCAATGTAAT | RT-qPCR analysis |
| qUFGT-F | GCTGACGAGTTGGGAGTGC | RT-qPCR analysis |
| qUFGT-R | CCTTCCGCTAAGTCTTTGATTC | RT-qPCR analysis |
| qFLS-F | ACGAGCAACCGGGAATCACAACTG | RT-qPCR analysis |
| qFLS-R | CCCAGTTGGAGCTGGCCTCAGTA | RT-qPCR analysis |
| qLAR1-F | TTTATCAAAGGATGCCAGGTT | RT-qPCR analysis |
| qLAR1-R | CATCCAAGGTCCTGAAAGAAT | RT-qPCR analysis |
| qANR1-F | AACCACAAGAAGGTCTCCCAC | RT-qPCR analysis |
| qANR1-R | CCCTTGGATTGCTGGTTTGAT | RT-qPCR analysis |
| qANR2-F | ACCCCTGTCAACTTTGCCTCA | RT-qPCR analysis |
| q-MD15G1078200-F | ATGGCCCTGGAACTAATGATGGGAT | RT-qPCR analysis |
| q-MD15G1078200-R | TCAAGACGATTCGAGGATAAGGGTG | RT-qPCR analysis |
| q-MD14G1227500-F | ATGTCGACTAAGACTAAAACCCTAA | RT-qPCR analysis |
| q-MD14G1227500-R | CTACATTTCGTCCGTGTTCCATATG | RT-qPCR analysis |
| q-MD13G1213100-F | ATGAATTACTCTACTTTTGATTCCC | RT-qPCR analysis |
| q-MD13G1213100-R | TCACCAATTAGGAGTGGCATTAGTA | RT-qPCR analysis |
| q-MD01G1226600-F | ATGGAAAGGAGGAAAGAGGAGTGCT | RT-qPCR analysis |
| q-MD01G1226600-R | TCAAGCCCAGAAATTTAACCAGTGT | RT-qPCR analysis |
| q-MD06G1217200-F | ATGTCCACTAATACTAAAACCCTAA | RT-qPCR analysis |
| q-MD06G1217200-R | CTACATGAAAAAATCACTGTCAAAC | RT-qPCR analysis |
| q-MD14G1120000-F | ATGAATCATCCAGCTGTCAAGTACA | RT-qPCR analysis |
| q-MD14G1120000-R | TCACAGGGCAACAAGAGGATCGGAA | RT-qPCR analysis |
| q-MD05G1349800-F | ATGGACAAAGGATGGGGGCTCACTC | RT-qPCR analysis |
| q-MD05G1349800-R | TTAATTTCCCGGGAAGCTGCTAATG | RT-qPCR analysis |
| q-MD13G1122100-F | ATGGAAAATTACCCAACATTCTTTT | RT-qPCR analysis |
| q-MD13G1122100-R | TTAAAATGGAGTGTAGATTTTCATC | RT-qPCR analysis |
| q-MD01G1177000-F | ATGGAAGAACAGGCTCAGCACTACA | RT-qPCR analysis |
| q-MD01G1177000-R | TTAGCTAACCAATAGTTGCTCGCCA | RT-qPCR analysis |
| q-MD13G1130700-F | ATGTCAGCCATGGTTTCTGCTCTCA | RT-qPCR analysis |
| q-MD13G1130700-R | CTACTCCCTTGGATTACTCGAATTC | RT-qPCR analysis |
| q-MD17G1049300-F | ATGTCTAGCAGAGGGTCAAGGCAGT | RT-qPCR analysis |
| q-MD17G1049300-R | CTACTGCGTAATCAAGCTCCTAATT | RT-qPCR analysis |
| PGADT7-MD15G1078200-F | ATACGACGTACCAGATTACGCTCAT ATGGCCCTGGAACTAATGATGGGAT | the vectors contruction of Y1H |
| PGADT7-MD15G1078200-R | CATCTGCAGCTCGAGCTCGATGGAT TCAAGACGATTCGAGGATAAGGGTG | the vectors contruction of Y1H |
| PGADT7-MD14G1227500-F | ATACGACGTACCAGATTACGCTCAT ATGTCGACTAAGACTAAAACCCTAA | the vectors contruction of Y1H |
| PGADT7-MD14G1227500-R | CATCTGCAGCTCGAGCTCGATGGAT CTACATTTCGTCCGTGTTCCATATG | the vectors contruction of Y1H |
| PGADT7-MD13G1213100-F | ATACGACGTACCAGATTACGCTCAT ATGAATTACTCTACTTTTGATTCCC | the vectors contruction of Y1H |
| PGADT7-MD13G1213100-R | CATCTGCAGCTCGAGCTCGATGGAT TCACCAATTAGGAGTGGCATTAGTA | the vectors contruction of Y1H |
| PGADT7-MD01G1226600-F | ATACGACGTACCAGATTACGCTCAT ATGGAAAGGAGGAAAGAGGAGTGCT | the vectors contruction of Y1H |
| PGADT7-MD01G1226600-R | CATCTGCAGCTCGAGCTCGATGGAT TCAAGCCCAGAAATTTAACCAGTGT | the vectors contruction of Y1H |
| PGADT7-MD06G1217200-F | ATACGACGTACCAGATTACGCTCAT ATGTCCACTAATACTAAAACCCTAA | the vectors contruction of Y1H |
| PGADT7-MD06G1217200-R | CATCTGCAGCTCGAGCTCGATGGAT CTACATGAAAAAATCACTGTCAAAC | the vectors contruction of Y1H |
| PGADT7-MD14G1120000-F | ATACGACGTACCAGATTACGCTCAT ATGAATCATCCAGCTGTCAAGTACA | the vectors contruction of Y1H |
| PGADT7-MD14G1120000-R | CATCTGCAGCTCGAGCTCGATGGAT TCACAGGGCAACAAGAGGATCGGAA | the vectors contruction of Y1H |
| PGADT7-MD05G1349800-F | ATACGACGTACCAGATTACGCTCAT ATGGACAAAGGATGGGGGCTCACTC | the vectors contruction of Y1H |
| PGADT7-MD05G1349800-R | CATCTGCAGCTCGAGCTCGATGGAT TTAATTTCCCGGGAAGCTGCTAATG | the vectors contruction of Y1H |
| PGADT7-MD13G1122100-F | ATACGACGTACCAGATTACGCTCAT ATGGAAAATTACCCAACATTCTTTT | the vectors contruction of Y1H |
| PGADT7-MD13G1122100-R | CATCTGCAGCTCGAGCTCGATGGAT TTAAAATGGAGTGTAGATTTTCATC | the vectors contruction of Y1H |
| PGADT7-MD01G1177000-F | ATACGACGTACCAGATTACGCTCAT ATGGAAGAACAGGCTCAGCACTACA | the vectors contruction of Y1H |
| PGADT7-MD01G1177000-R | CATCTGCAGCTCGAGCTCGATGGAT TTAGCTAACCAATAGTTGCTCGCCA | the vectors contruction of Y1H |
| PGADT7-MD13G1130700-F | ATACGACGTACCAGATTACGCTCAT ATGTCAGCCATGGTTTCTGCTCTCA | the vectors contruction of Y1H |
| PGADT7-MD13G1130700-R | CATCTGCAGCTCGAGCTCGATGGAT CTACTCCCTTGGATTACTCGAATTC | the vectors contruction of Y1H |
| PGADT7-MD17G1049300-F | ATACGACGTACCAGATTACGCTCAT ATGTCTAGCAGAGGGTCAAGGCAGT | the vectors contruction of Y1H |
| PGADT7-MD17G1049300-R | CATCTGCAGCTCGAGCTCGATGGAT CTACTGCGTAATCAAGCTCCTAATT | the vectors contruction of Y1H |
| MD06G1217200-pro-NdeI-F | ATACGACGTACCAGATTACGCTCAT CTACATGAAAAAATCACTGTCAAAC | the vectors contruction of overexpression |
| MD06G12172000-pro-BamHI-R | CATCTGCAGCTCGAGCTCGATGGAT ATGAATCATCCAGCTGTCAAGTACA | the vectors contruction of overexpression |
| MD06G1217200-RNAi-Xba1 F | GAGAGAACACGGGGGACTGTTGAAGCAGAAGATAGGAC | the vectors contruction of interference |
| MD06G1217200-RNAi-Sal1 R | ATTGGGGTTCCCCCGGGGATGTCGTAGCCATTGTTGTC | the vectors contruction of interference |
| MD06G1217200-RNAi-Kpn1 F (Reverse) | CTGGGTTCGAAATCGATGGTACATGTCGTAGCCATTGTTGTC | the vectors contruction of interference |
| MD06G1217200-RNAi-EcoR1 R (Reverse) | GCCAGGAGAGTTGTTGATTCAGGTTGAAGCAGAAGATAGGAC | the vectors contruction of interference |
